# Oral Bacteria, Virus and Fungi in Saliva and Tissue Samples from Adult Subjects with Oral Squamous Cell Carcinoma: An Umbrella Review

**DOI:** 10.3390/cancers15235540

**Published:** 2023-11-22

**Authors:** Federica Di Spirito, Maria Pia Di Palo, Veronica Folliero, Davide Cannatà, Gianluigi Franci, Stefano Martina, Massimo Amato

**Affiliations:** Department of Medicine, Surgery and Dentistry, University of Salerno, 84084 Salerno, Italy; mariapia140497@gmail.com (M.P.D.P.); vfolliero@unisa.it (V.F.); davide2897@icloud.com (D.C.); gfranci@unisa.it (G.F.); mamato@unisa.it (M.A.)

**Keywords:** oral squamous carcinoma of head and neck, mouth, tissues, saliva, microbiota, viruses, bacteria, fungi, dysbiosis, carcinogenesis

## Abstract

**Simple Summary:**

Given the putative or recognized role of oral microorganisms and oral dysbiosis in oral carcinogenesis and the technological advances in microbial research, enabling to obtain a more comprehensive and exhaustive microbiological profile of the oral cavity under healthy and diseased conditions, this umbrella review aimed to comprehensively describe the oral microorganisms (bacteria, viruses, and fungi) found in adults with oral squamous cell carcinoma by examining the microbiological content of tissue and saliva samples. Knowledge of the microbial picture of individuals with oral carcinomas is essential to fully understand the possible or recognized carcinogenic role of oral microorganisms in developing oral squamous cell carcinomas.

**Abstract:**

Oral squamous cell carcinoma (OSCC) is the most common oral cavity malignancy associated with multiple risk factors. In the last 14 years, oral dysbiosis has attracted the scientific community’s attention as a potential oncogenic factor, in parallel with the development of omics technologies that have revolutionized microbiological research. The present umbrella review aimed to investigate the oral microbiological content (bacilli, viruses, and fungi) of tissue and saliva samples from adult (>18 years) patients with OSCC. The secondary objective was to compare the oral microbiome of OSCC subjects with non-OSCC subjects. The study protocol was under the PRISMA statement and registered on PROSPERO (CRD42023448153). Data from 32 systematic reviews were extracted, qualitatively summarized, and analyzed using AMSTAR-2. An increase in oral bacteria of the phylum Fusobacteria, Proteobacteria, and Bacteroidetes and a decrease in Firmicutes and Actinobacteria were observed in OSCC patients. The increased bacterial genera were periodontopathogens. The most common viruses were EBV and HPV, especially the high-risk genotypes. Candida was the most studied oral fungus and was always increased in OSCC subjects. Further studies should investigate the possible carcinogenic mechanisms of oral microorganisms found increased in tissue samples and saliva from adult subjects with OSCC.

## 1. Introduction

Oral squamous cell carcinoma (OSCC) is the most common oral cavity malignancy [1,2,3]. It accounts for approximately 90% of all oral cancers and ranks 16th worldwide in incidence and mortality [2,4,5].

The International Agency for Research on Cancer (IARC) reported that there were approximately 400,000 new cases of lip/oral cavity cancer in both sexes and all ages worldwide in 2020, with a mortality rate of 178,000 (data freely available online on https://gco.iarc.fr/today/fact-sheets-cancers (accessed on 24 July 2023)). 

OSCC is associated with high mortality due to its invasiveness, early metastasis, rapid progression, and poor prognosis [3], with a 5-year survival rate of 80–90% for early-stage OSCC and 30–50% for advanced-stage OSCC [4], and may negatively affect oral cavity functions such as speech, chewing, and facial appearance [1]. Early diagnosis is associated with a better prognosis and the possibility of intervening with promising new alternative treatments that are less invasive for the patient, such as photodynamic and sonodynamic therapy, compared to the more common surgical resection, chemotherapy, radiotherapy, such as photodynamic therapy [6,7].

Several risk factors have been associated with OSCC, such as tobacco (smoked or smokeless), alcohol consumption, poor oral hygiene, chronic irritability, infections, genetic disorders, and sun exposure (for lip carcinomas) [3,4,5]. Differences in exposure to those carcinogenic risk factors are thought to be related to differences in worldwide OSCC prevalence [2]. 

In addition, dysbiosis of the oral microbiome as a potential oncogenic factor of OSCC has attracted the scientific community’s attention in recent years [3]. According to current knowledge, some oral microorganisms are involved in oral carcinogenesis by either establishing a chronic inflammatory environment, synthesizing carcinogens, or altering the integrity of the oral epithelial barrier [2].

The oral bacteriome is the major component of the oral microbiome and includes more than 600 different bacterial species [8]. The role of bacteria in cancer pathogenesis was already known in the past two decades for gastric cancer and lymphoma of mucosa-associated lymphoid tissue associated with *Helicobacter pylori*, cervical cancer with *Chlamydia trachomatis*, gallbladder cancer with *Salmonella typhi*, and colon cancer with *Fusobacterium nucleatum (F. nucleatum)* and *Bacteroides fragilis* [3,8]. 

More recently, oral dysbiosis and related local and systemic inflammation associated with periodontitis have been associated with various forms of extraoral malignancies [9,10], such as lung [11], prostate [12], pancreatic [9], colorectal [13], breast [14] cancers, as well as head and neck cancer [15] and oral squamous cell carcinoma (OSCC) [16]. Oral and periodontal bacteria are thought to be promoters in oral and extraoral carcinogenesis [10]. In detail, *F. nucleatum* and *Porphyromonas gingivalis* are the most studied periodontal pathogens in OSCC carcinogenesis [17,18]. However, other microorganisms found augmented in oral potentially malignant disorders (OPMDs) or OSCC compared to healthy individuals may be involved in oral carcinogenesis [8,17,18,19].

The carcinogenic role of viruses was first identified at least 100 years ago by Peyton Rous, who demonstrated that chicken sarcoma could be caused by Rous Sarcoma Virus [20,21]. Subsequent studies on cancer-related viruses in humans led the IARC to classify the following viruses as Group 1 carcinogens in humans: Epstein–Barr Virus (EBV), Human Papilloma Virus (HPV), Human T cell Lymphotropic Virus type-1 (HTLV-1), and Kaposi’s Sarcoma Herpes Virus (KSHV), because they are direct carcinogens; Hepatitis B Virus (EBV) and Hepatitis C Virus (HCV) as indirect carcinogens that can induce a chronic inflammatory process; Human Immunodeficiency Virus type-1 (HIV-1) as an indirect carcinogen that can cause immunodepression [22]. Some viruses can cause more than one different form of cancer, and the same can be caused by more than one virus [20,21,22]. 

Although the association between oral viruses and OSCC is still controversial, several studies have suggested the involvement of oral viruses in the development of OSCC, focusing on HPV, EBV, HCV, and Herpes Simplex Virus Type 1 (HSV-1) [20,21,23,24,25].

To a lesser extent than bacteria or viruses, the oral microbiome includes commensal microorganisms from the fungal kingdom, such as yeast of the genus *Candida*, *Cladosporium*, *Aspergillus*, *Acremonium*, *Aureobasidium*, *Mallasezia*, *Morchella*, and others [19,26]. *Candida albicans* is the most common genus among oral yeasts and is hosted as a commensal fungus in the oral cavity in about 40–65% of healthy adults [8,26]. Nonetheless, it can be responsible for opportunistic infections, especially in immunocompromised conditions [26,27], such as HIV/Acquired Immunodeficiency Syndrome, infancy senility, or malignancies [28], determining acute or chronic oral candidiasis [8,26]. Moreover, through cross-kingdom interactions with various oral microorganisms, *Candida albicans* has been involved in periodontitis, peri-implantitis, dental caries, and endodontic infections [29]. Furthermore, *Candida albicans* has been implied in genesis OPMD, and OSCC is generally hosted as a commensal fungus in the oral cavity [8,19,27,28,29].

The development of omics technologies (metagenomics, metatranscriptomics, metaproteomics, and metabolomics) has revolutionized microbiology research [8,30]. New culture-independent laboratory techniques for identifying microorganisms, such as next-generation sequencing (NGS) that identifies the sequence of nucleotides in genomes or target DNA or RNA regions such as the hypervariable regions of 16S ribosomal subunits, have enabled more comprehensive and accurate profiling of the oral microbiome under healthy and diseased conditions in recent years and have expanded the relatively small number of microorganisms that could be studied with culture-dependent techniques [8,30,31].

Given the putative or recognized role of oral microorganisms in oral carcinogenesis and the development of new technologies, the present umbrella review aimed to evaluate the oral microbial (bacteria, viruses, and fungi) content of neoplastic tissue samples and saliva from adult (>18 years old) OSCC subjects. This review also aimed to compare the microbial content in OSCC and non-OSCC subjects.

## 2. Materials and Methods

### 2.1. Study Protocol

The study protocol—compliant with the Preferred Reporting Items for Systematic Reviews and Meta-analyses (PRISMA) statement [32] and recorded on the International Prospective Register of Systematic Review PROSPERO register (CRD42023448153)—was defined prior to beginning the literature search, data extraction, and analysis.

The research focused on the following question: “What are the oral microorganisms (bacteria, viruses, and fungi) found in tissue samples and/or saliva in adult OSCC subjects?”.

The definition of the question, search strategies, and study selection criteria were based on the PICO model [33] as follows:

(P): Population: adult subjects (≥18 years of age) with OSCC;
(I): Intervention: histopathologic analysis of OSCC lesions and/or saliva testing in OSCC patients;
(C): Comparison: no histopathologic analysis or saliva testing; histopathologic analysis of non-OSCC tissue or saliva testing in non-OSCC subjects;
(O): Outcome(s): microbial content and composition of saliva or OSCC samples in adult subjects.

### 2.2. Search Strategy

An electronic search of systematic reviews with or without meta-analysis was performed till 29 June 2023, by two independent reviewers (F.D.S. and M.P.D.P.). The following databases and registers were consulted, and filters were used according to availability:

PubMed/MEDLINE: Article type “Systematic Review” and “Meta-analysis”; Language “English”.
Scopus: Document type “Review”; Language “English”.
Web of Science: Document types “Review Article”; Languages “English”.
BioMed Central: no filter.
PROSPERO register: Status of the review “Published”.
No date restrictions were applied.

The following keywords were used and combined using Boolean operators: (“oral squamous cell carcinoma” OR “oral cancer” OR “oral carcinoma” OR “oral carcinogenesis”) AND (bacteria OR bacterium OR virus OR viruses OR fungi OR fungus OR mycete OR “bacterial infection” OR “viral infection” OR “fungal infection” OR “oral microbiome” OR “oral microbioma” OR “oral microbiota” OR “oral microorganism” OR “oral dysbiosis”).

### 2.3. Study Selection and Eligibility Criteria

After establishing the eligibility criteria, two independent reviewers performed the study selection (F.D.S., M.P.D.P.). Issues of disagreement were resolved by discussion with a third reviewer (D.C.).

Titles and abstracts obtained from the electronic search were screened to remove duplicates or records not relevant to OSCC or our purpose. The full text was obtained for unclear titles and abstracts before eventual exclusion. If the full text was not available, the authors were contacted. An additional manual search was performed by consulting the references of included articles to find other eligible records.

All references of included studies were tabulated using Mendeley Reference Manager.

Inclusion criteria were systematic reviews with or without meta-analysis published in English, without limitation of date, sample size, and gender, concerning studies in humans that assessed using the stated methods the microbiological content of saliva or OSCC sample in adult subjects (≥18 years of age).

Exclusion criteria were systematic reviews with or without meta-analysis regarding microbiological analysis of saliva or oral mucosal samples in subjects without OSCC, studies in animals or in vitro models, and studies in humans younger than 18 years of age.

### 2.4. Data Extraction and Collection

The data were extracted by two independent reviewers (F.D.S. and M.P.D.P.) and collected using a standardized form for data extraction, created in accordance with the models proposed for intervention reviews of RCTs and non-RCTs [34]. The disagreement between the two independent reviewers was resolved by a discussion with a third reviewer (D.C.).

The data extracted and collected from each systematic review, with or without meta-analysis included in this study, were as follows:

Study characteristics: first author, year, journal, design and number of studies reported, meta-analysis, study quality, funding;
Population characteristics: sample size, mean age, gender ratio, country of origin of the sample, risk factors for OSCC, history of OPMD or malignancies, other comorbidities and ongoing treatments;
OSCC characteristics: macroscopic features, location, staging, grading, microscopic features, first diagnosis (primary site/metastatic lesion), time to onset, chemotherapy (yes/no), radiotherapy (yes/no);
Intervention: number of samples, method(s) of sample collection, microorganisms identification technique and target;
Outcome(s):
Bacteria: type(s) of phylum, genus and species of bacterium detected, number or percentage of positive OSCC cases;
Viruses: type(s) and genotype(s) of virus detected, number or percentage of positive OSCC cases;
Fungi: type(s) and species of fungus detected, number or percentage of positive OSCC cases.

Data registered in the record included in the present umbrella review regarding other non-OSCC cancers were not extracted and collected.

### 2.5. Data Synthesis

A narrative synthesis of data on population characteristics and methods of investigating the microbiological content of saliva or OSCC samples in adult subjects was conducted.

The Microsoft Excel software 2019 (Microsoft Corporation, Redmond, WA, USA) was used for the qualitative synthesis through a descriptive statistical analysis of the data extracted from the included studies:

To characterize the macroscopic and microscopic features of OSCC samples in adult subjects (>18 years old) in relation to the microbiological content of the saliva or OSCC-tissue sample;

To characterize the microbiological content (bacterial, viral, and fungal) found in saliva or histopathologic analysis of samples in adult OSCC subjects;

To compare the microbiological content found in saliva or histopathologic analysis of OSCC samples with healthy controls or with OPMD;

To compare the microbiological content (bacterial, viral, and fungal) found in the saliva of OSCC subjects with that of OSCC sample tissues;

To provide an overall picture of the predominant or decreased microorganisms found in saliva or histopathologic analysis of samples in adult OSCC subjects.

### 2.6. Quality Assessment

The studies included in the present study were qualitatively assessed by two independent authors (F.D.S. and M.P.D.P.) on 29 June 2023, using the tool for quality valuation of systematic reviews of randomized and non-randomized studies: the Assessing the Methodological Quality of Systematic Reviews 2 (AMSTAR) accessible online (https://amstar.ca/ (accessed on 29 June 2023)). In case of disagreement in the assessment of included studies, a third reviewer was consulted for discussion (D.C.).

## 3. Results

### 3.1. Study Selection

A total of 914 records were retrieved by the electronic searches: 82 from MEDLINE/PubMed, 477 from Scopus, 318 from Web of Science, 35 from BioMed Central databases, and 2 from the PROSPERO register. Then, 235 duplicate records were removed before screening. The remaining 679 titles/abstracts were screened, and 385 were considered not relevant to the topic of the present study. The remaining 294 records were assessed for their eligibility, and the full-texts were screened, where 264 were excluded because 214 were narrative or scoping reviews; 26 were original articles; 12 did not evaluate the microbiological content; 5 did not evaluate the microbiological content in OSCC-subjects; 3 did not make it possible to extract data on the saliva or OSCC-tissue samples microorganisms content; 2 were not possible to extract data on the OSCC-subjects; 2 were in vitro or animals studies.

A total of 30 records [35,36,37,38,39,40,41,42,43,44,45,46,47,48,49,50,51,52,53,54,55,56,57,58,59,60,61,62,63,64], compliant with the eligibility criteria, were included in this umbrella review before the electronic search.

The reference list of the 30 studies included was screened to identify other relevant articles through the manual search that retrieved a total of 2142 records. We removed 94 duplicates, and of the 2048 remaining titles/abstracts, 2008 were considered not eligible. Of the 40 records assessed for eligibility, we screened the full texts, and an additional 38 articles were excluded because 23 were original articles, 7 were narrative or scoping reviews, 4 did not make it possible to extract data on the saliva or OSCC-tissue samples microorganisms content, 2 did not evaluate the microbiological content in OSCC-subjects, and 2 did not make it possible to extract data on the OSCC-subjects.

A total of two records [65,66], compliant with the eligibility criteria, were included in this umbrella review before the manual search.

Finally, the present study included 32 articles [35,36,37,38,39,40,41,42,43,44,45,46,47,48,49,50,51,52,53,54,55,56,57,58,59,60,61,62,63,64,65,66] on the microbiological content of saliva or OSCC-tissue samples in adult subjects (>18 years of age) with OSCC.

**Figure 1:**
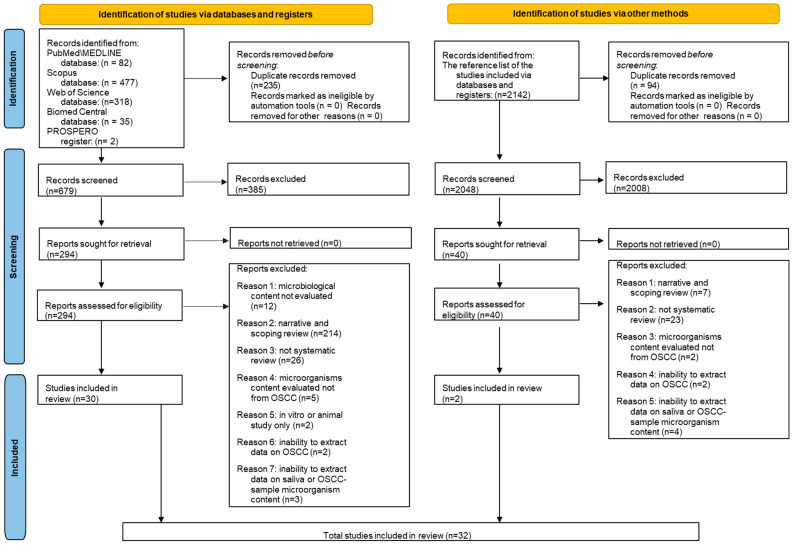
PRISMA 2020 flowchart for systematic reviews, which included searches of databases, registers, and using other methods.

Figure 1 shows the PRISMA 2020 flowchart of study selection following the electronic and manual search.

Data from 32 studies [35,36,37,38,39,40,41,42,43,44,45,46,47,48,49,50,51,52,53,54,55,56,57,58,59,60,61,62,63,64,65,66] on the microbiological content (bacteria, viruses, or fungi) of saliva or OSCC samples in adult subjects (>18 years of age) with OSCC diagnosed through clinical examination and confirmed based on histopathologic analysis were extracted and qualitatively synthesized in two tables: Table 1 reports data from studies that evaluated the microbial content of OSCC samples and Table 2 reports data from studies that evaluated the microbial content of saliva in subjects with OSCC. Data from included studies [35,42,43,45,47,52,54,55,58,64] that evaluated both types of samples were divided and reported separately in the corresponding tables. Only data compliant with the eligibility criteria were extracted, so data from other types of non-OSCC cancers, from districts different from the oral cavity, or from pediatric subjects were not considered.

Of the 32 studies included [35,36,37,38,39,40,41,42,43,44,45,46,47,48,49,50,51,52,53,54,55,56,57,58,59,60,61,62,63,64,65,66], 20 were systematic reviews with meta-analysis [36,37,38,41,44,46,49,50,53,54,56,57,59,60,61,62,63,64,65,66] and 12 without meta-analysis [35,39,40,42,43,45,47,48,51,52,55,58]. The 32 systematic reviews included 642 studies that met the eligibility criteria of this umbrella review, in particular, 248 case-control studies, 29 cohort studies, 20 cross-sectional studies, 10 non-randomized studies, 2 retrospective cross-sectional studies, 1 cross-sectional cohort study, 1 prospective study, and 331 articles where the study design had not been defined.

### 3.2. Study Characteristics and Qualitative Synthesis

Table 1, Table 2 and Table 3 summarize the studies included and data from included systematic reviews describing the microbiological content (bacteria, viruses, and fungi) of the OSCC-tissue samples of adult subjects (>18 years of age). Supplementary File S1, containing Supplementary Table S1 describing all outcomes together, has been added.

Table 4, Table 5 and Table 6 summarize the studies included and data from included systematic reviews describing the microbiological content (bacteria, viruses, and fungi) of the OSCC-tissue samples of adult subjects (>18 years of age). Supplementary File S2, containing Supplementary Table S2 separately describing all outcomes together, has been added.

### 3.3. Oral Bacterial Content in Adult Subjects with OSCC: Study Characteristics and Qualitative Synthesis

Eleven studies [36,42,43,45,47,48,50,52,55,58,62] described the oral bacterial content in adult subjects with OSCC, in particular: eight studies [42,43,45,47,52,55,58,62] of the OSCC-tissue samples and saliva, one study [48] only in the OSCC-tissue samples, and two studies [36,50] only of the saliva.

#### 3.3.1. Oral Bacterial Content in OSCC-Tissue Samples of Adult Subjects

Ten studies [36,42,43,45,47,50,52,55,58,62] described the bacterial content of the OSCC-tissue samples of adult subjects (Table 1).

Nine studies [36,42,43,45,50,52,55,58,62] reported a sample size of the OSCC group amounting to 2.562, eight studies [42,43,45,50,52,55,58,62] involved a healthy control group evaluating 2.321 cases, two studies [50,55] a group with OPMD of 90, and one study [55] a group with oral fibroepithelial polyps of 27 subjects.

The mean age was recorded by three studies [42,55,62], which was stated to be 56.47 years old, while the gender ratio (males/females) by one study [43] (1:1.13).

The reported country of origin of the samples was China (n = 323) [36,42,43,58], the United States of America (n = 232) [36,42,55,58], Germany (n = 224) [36,43], Sri Lanka (n = 130) [36,42,43,58], India (n = 100) [36,43], Iran (n = 83) [43], Japan (n = 79) [36,43], Yemen (n > 40) [36,42,58], Hungary (n = 31) [36], Wales (n = 30) [36], United Kingdom (n = 10) [42], Malaysia (n = 9) [55], and Saudi Arabia (number not defined) [36].

As risk factors for OSCC, two studies [52,58] specified the use of tobacco, alcohol, and betel [43,58], and one study [58] of smokeless tobacco (Shammah).

Three studies [36,42,52] pointed to the tongue as the location of OSCC in more than 78 cases.

The method of sample collection most used was biopsy (n > 1.993) [36,42,43,45,47,50,52,58,62], followed by swabs (n > 364) [36,42,43,47,52,55], brush (n > 91) [47,50], blood analysis (n = 50) [50], both biopsy and swab (n = 40) [36], biofilm sampling (n = 21) [50], and sterile paper point (n = 3) [36].

The microorganisms identification technique included: PCR (n = 330) [36,43,47,50]; culture (n = 294) [36,43,47,50]; IHC (n > 274) [43,47]; ELISA (n = 253) [43,50]; nested PCR (n = 120) [50]; MiSeq (n = 85) [52]; QIAamp DNA Mini kit for DNA extraction (n = 80) [42]; GIEMSA (n = 68) [43]; QIAamp FastDNA Stool Mini kit (n = 61) [58]; RT-PCR (n = 58) [43]; TIANamp swab DNA kit (n = 50) [58]; Gentra Puregene tissue kit (n = 50) [58]; QIAamp DNA Stool Mini kit for DNA extraction (n = 39) [42]; ion torrent (n = 39) [52]; DNA isolation kit [42,58]; not defined next-generation sequencing technology (n = 30) [62]; incubation in proteinase K and DNA easy kit (n = 15) [42]; DNA extraction kit (n = 9) [55].

The sourced target through the microorganisms identification technique was: 16s rRNA (n = 192) [36,42,50,55]; 16s rDNA (n = 42) [36]; V1-V3 region (n = 155) [42,52,58,62]; V3-V4 region (n = 135) [58]; V4-V5 region (n = 130) [42,52]; V4 region (n = 119) [42,52,55,62]; RFLP gene (n = 9) [55].

The phyla of bacteria detected and whose variations have been investigated in the systematic reviews included in this study were *Fusobacteria* [42,45,52,55]; *Firmicutes* [42,52,55,62]; *Proteobacteria* [42,55,62]; *Bacteroidetes* [42,45,52,55,62]; *Actinobacteria* [42,55,62]; *Spirochaetes* [42].

**Figure 2:**
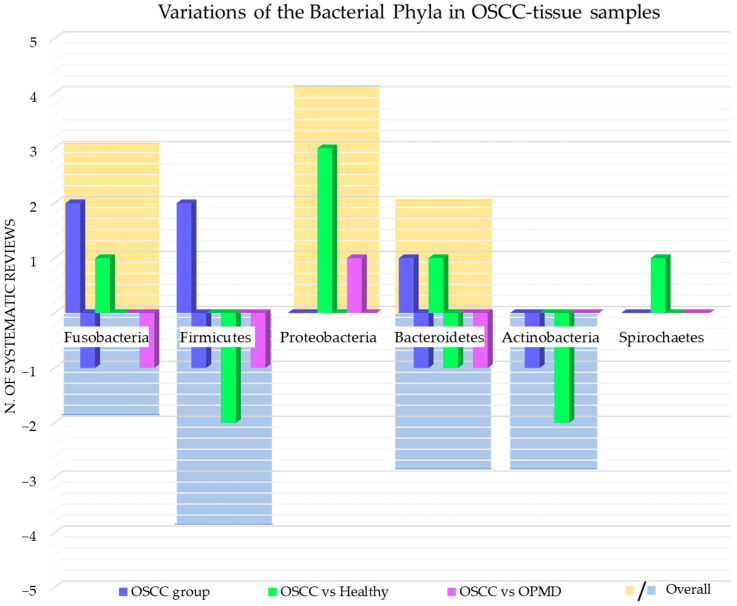
Systematic reviews that reported any variations in the bacterial phyla content in the OSCC-tissue samples of adult subjects, the comparison with the healthy and the OPMD control group, and the final number of systematic reviews that reported an increase (positive number) or decrease (negative number) for each bacterial phyla. Notes: studies investigating variations in different groups were counted separately for each group.

Figure 2 shows the number of systematic reviews that reported any variations in the bacterial phyla content in the OSCC-tissue samples of adult subjects, the comparison with the healthy and OPMD control group, and the final number of systematic reviews that reported an increase or decrease for each bacterial phylum.

The genera of bacteria detected and whose variations have been investigated were: *Fusobacterium* [36,42,45,50,52,55,58,62]; *Staphylococcus* [58]; *Streptococcus* [42,50,55,58,62]; *Peptostreptococcus* [42,45,52,58]; *Peptococcus* [52]; *Enterococcus* [42]; *Dialister* [45,52]; *Parvimonas* [45,52,58]; *Gemella* [42,55]; *Catonella* [52]; *Veillonella* [55]; *Johnsonella* [42]; *Granulicatella* [55]; *Paenibacillus* [58]; *Filifactor* [52]; *Corynebacterium* [42]; *Propionibacterium* [58]; *Atopobium* [42,52,58]; *Micrococcus* [58]; *Mobilinicus* [58]; *Clavibacter michiganensis* [42]; *Actinomyces* [42,58]; *Microbacterium* [58]; *Arthrobacter* [58]; *Rothia* [42,55,58,62]; *Campylobacter* [42,52,58]; *Pseudomonas* [42,58]; *Aeromonas* [58]; *Brevendimonas* [58]; *Ralstonia* [42]; *Sphingomonas* [58]; *Neisseria* [50,55]; *Frauteria* [58]; *Haemophilus* [62]; *Caulobacter* [58]; *Lautropia* [58]; *Capnocytophaga* [42,45,52,58]; *Prevotella* [50,58,62]; and *Porphytomonas* [45,58].

**Figure 3:**
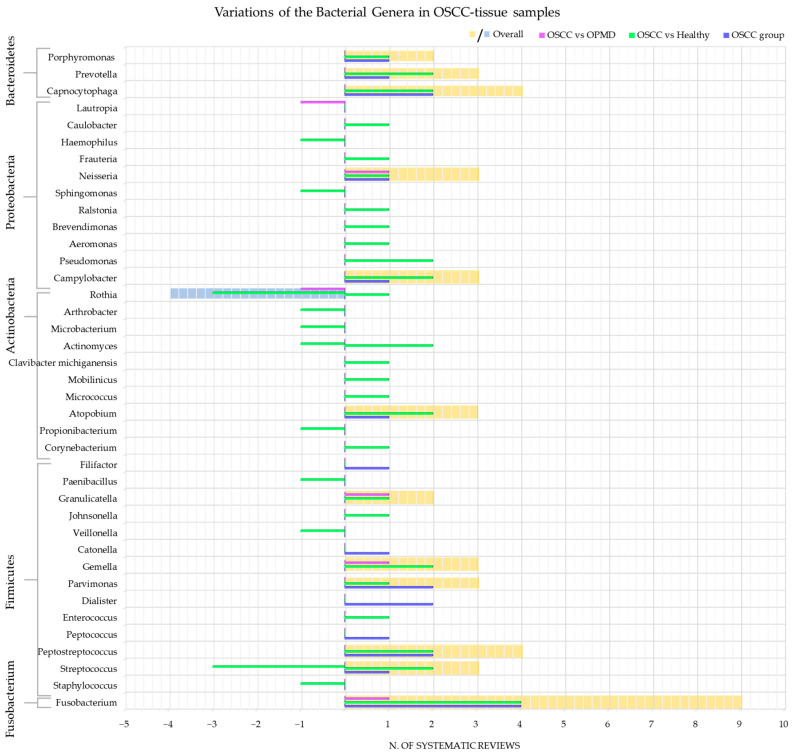
Systematic reviews that reported any variations in the bacterial genera content in the OSCC-tissue samples of adult subjects, the comparison with the healthy and the OPMD control group, and the final number of systematic reviews that reported an increase (positive number) or decrease (negative number) for each bacterial genus. Notes: studies investigating variations in different groups were counted separately for each group.

Figure 3 shows the number of systematic reviews that reported any variations in the bacterial genera content in OSCC-tissue samples of adult subjects, the comparison with the healthy and OPMD control group, and the final number of systematic reviews that reported an increase or decrease for each bacterial genus.

The species and subspecies of bacteria detected were: *F. nucleatum* [36,58] (subspecies *polymoprhum* [42,52,58], *nucleatum* [36], *vicentii*, *animalis* [36]), *F. naviforme* [36,42], *F. periodonticum* [36], *F. canifelinum* [36], *F. oralis* (subspecies *oral taxon A71, 203, 370* [36,58]), *F. necrophorum* [36], *F. gonidiaformans* [36], *F. simiae* [36], *F. parvimonas* [58]; *Leptotrichia oral taxon 225* [58]; *Staphylococcus epidermidis* [58]; *Streptococcus (S.) dysgalactiae* [58], *S. parasanguinis* [42,58], *S.mitis* [58], *S. gordonii* [42], *S. salivarius* [42], *S. agalactiae* [58], *S. oralis* (subspecies *oral taxon 423* [58], *070* [58], *431* [58], *058* [42]) [58]; *Peptostreptococcus stomatis* [42,58]; *Gemella morbillorum* [42,58], *Gemella haemolysans* [42]; *Johnsonella ignava* [42]; *Granulicatella adicens* [58], *Granulucatella elegans* [58]; *Corynebacterium matruchotii* [58]; *Clavibacter michiganensis tellarius* [42]; *Rothia dentocariosa* [58], *Rothia mucilaginosa* [58]; *Campylobacter (C.) oralis* (subspecies *taxon 44*) [42], *C. concisius* [58], *C. rectus* [58]; *Pseudomonas aeruginosa* [42,52,58]; *Ralstonia insidosa* [42]; *Lautropia mirabilis* [58]; *Helicobacter pylori* [43,47]; *Citrobacter koseri* [58]; *Aggregatibacter segnis* [58]; *Capnocytophaga leadbetteri* [58]; *Prevotella salivae* [58], *Prevotella loeschii* [58], *Prevotella intermedia* [58]; *Porphyromonas cationiae* [58]; *Porphyromonas gingivalis* [58].

None of the remaining extracted records were reported.

**Figure 4:**
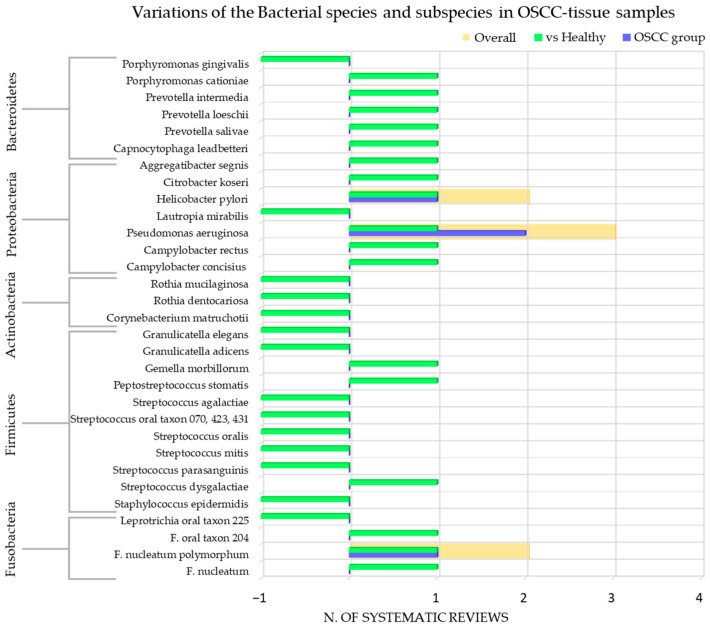
Systematic reviews that reported any variations in the bacterial species/subspecies content in the OSCC-tissue samples of adult subjects, the comparison with the healthy control group, and the final number of systematic reviews that reported an increase (positive number) or decrease (negative number) for each bacterial species/subspecies. Notes: studies investigating variations in different groups were counted separately for each group.

Figure 4 shows the number of systematic reviews that reported any variations in the bacterial species/subspecies content in the OSCC-tissue samples of adult subjects, the comparison with the healthy control group, and the final number of systematic reviews that reported an increase or decrease for each bacterial species/subspecies.

#### 3.3.2. Oral Bacterial Content in Saliva of Adult Subjects with OSCC

Nine studies [42,43,45,47,48,52,55,58,62] described the bacterial content in the saliva of adult subjects with OSCC (Table 4).

Eight studies [42,43,45,48,52,55,58,62] reported a sample size of the OSCC group amounting to 3.654 and a healthy control group of 3.667 cases; three studies [48,52,55] also involved an OPMD control group evaluating 482 cases; one study [48] a periodontitis control group of 15 cases; and in another study [58] a control group with 12 cases of benign or malignant thyroid nodules.

The mean age was recorded by three studies [42,48,62], which was stated to be 53.77 years of age, while the age range was reported by two studies [48,62] (32–87 years of age).

The gender ratio (males/females) was specified by one study [48] (1.58:1).

The reported country of origin of the samples was Taiwan (n > 1.032) [42,48,55,58]; the United States of America (n = 395) [42,48,55,58]; China (n > 206) [48,55,58]; Japan (n = 132) [55,58]; India (n = 107) [43,55,58]; Australia (n = 63) [58]; Sudan (n = 59) [48]; New Zealand (n = 23) [58]; Malaysia (n = 9) [58].

As a risk factor for OSCC, one study [52] specified the use of tobacco, alcohol, or betel in the OSCC group.

The staging of the tumor was reported by one study [52] as follows: stage I (n = 41), stage II (n = 66), and stage IV (n = 90).

The method of sample collection most used was the saliva test (n > 1.773) [42,43,45,47,52,55,58,62], followed by oral rinse (n = 1.025) [48,52,55,58,62], sputum (n = 448) [48], oral rinse (n > 352) [47,48,58], both swab and saliva test (n = 29) [55], and oral brush (n = 27) [58].

The microorganisms identification technique included: QIAamp DNA Blood Mini kit (n = 596) for DNA extraction [48,55,58]; not defined next-generation sequencing technologies (n = 413) [62]; MiSeq (n = 333) [52]; QIAamp DNA Microbiome kit (n = 197) [58]; Power Soil DNA Isolation kit (n = 129) [58]; QIAamp Mini Elute Virus Spin kit (n = 138) [58]; QIAamp DNA Blood kit (n = 127) [42]; QIAsymphony virus/bacteria Midi kit (n = 121) [58]; gene Prep Star PI-80X device (n = 120) [48,58]; QIAamp DNA Mini kit (n = 101) [55]; Maxwell 16 LEV Blood DNA kit (n = 63) [58]; Fast DNA (n = 59) [48]; modified QIAGEN DNA extraction (n = 62) [48,55,58]; culture (n > 50) [43,47]; DNA easy blood and tissue kit (n = 50) [58]; E.Z.N.A. soil DNA kit (n = 47) [48]; HiPure tissue and blood DNA kit (n = 32) [55]; gene Fix saliva Prep 2 isolation kit (n = 31) [55]; QIAGEN DNeasy Blood and Tissue kit DNA (n = 26) [58]; purification kit (n = 26) [58]; phenol-chloroform based DNA extraction (n = 23) [58]; QIAamp Fast DNA Stool Mini kit (n = 10) [48]; EUX commercial kit with modifications (n = 9) [58]; 454/GS Junior (n = 6) [52]; DNA purification kit (n = 3) [58]; incubation in Proteinase K and DNA purification kit (n = 3) [42]; PCR, IHC, spectrophotometer, and gas chromatography [47] (number not defined).

The sourced target through the microorganisms identification technique was the V4 region (n = 2.122) [41,48,52,55,58,62]; the V3–V4 region (n = 1.091) [48,52,55,58,62]; the V3-V5 region (n = 292) [48,52,58,62]; the V4-V5 region (n = 231) [42,48,52,58,62]; 16s RNA (n = 177) [55]; the V6-V8 region (n = 73) [58,62]; ITS1 gene (n = 32) [55]; the V6-V9 region (n = 9) [58].

The phyla of bacteria detected and whose variations have been investigated in the systematic reviews included in this study were: *Fusobacteria* [45,48,58,62]; *Firmicutes* [45,48,55,58,62]; *Actinobacteria* [48,58,62]; *Proteobacteria* [48,52,62]; *Bacteroidetes* [45,48,55,58,62]; *Spirochaetes* [48,58]; *Saccharibacteria* [55].

**Figure 5:**
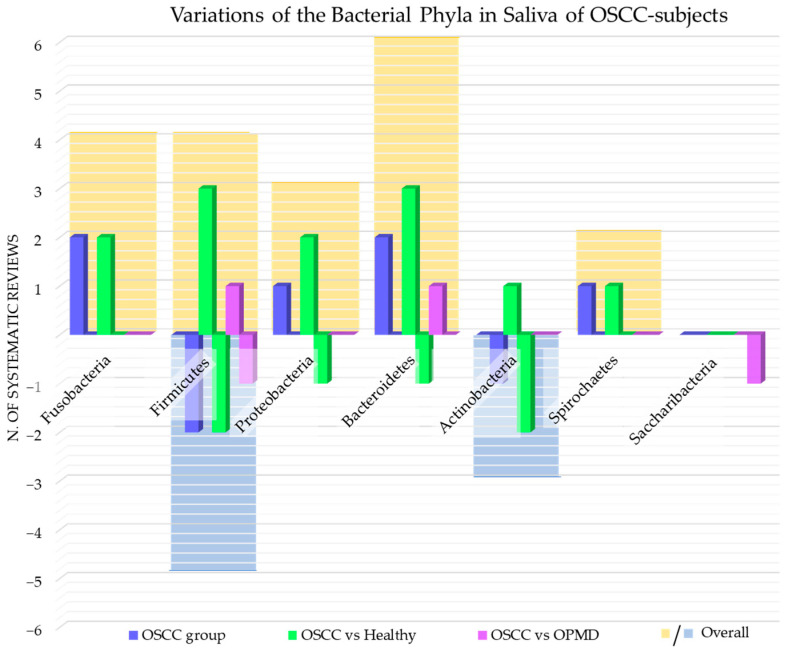
Systematic reviews that reported any variations in the bacterial phyla content in the saliva of adult subjects with OSCC, the comparison with the healthy and the OPMD control group, and the final number of systematic reviews that reported an increase (positive number) or decrease (negative number) for each bacterial phyla. Notes: studies investigating variations in different groups were counted separately for each group.

Figure 5 shows the number of systematic reviews that reported any variations in the bacterial phyla content of saliva of adult subjects with OSCC, the comparison with the healthy and OPMD control group, and the final number of systematic reviews that reported an increase or decrease for each bacterial phylum.

The genera of bacteria detected and whose variations have been investigated were: *Fusobacterium* [45,48,55,58,62]; *Leptotrichia* [58]; *Streptococcus* [42,45,48,52,55,58,62]; *Peptostreptococcus* [42,45,48,58]; *Peptococcus* [48,58]; *Enterococcus* [52,55]; *Dialister* [45,48,55,58]; *Parvimonas* [45,48,52,58]; *Gemella* [42,58]; *Catonella* [48,58]; *Veillonella* [48,52,55,58,62]; *Granulicatella* [48,58]; *Filifactor* [48,58]; *Oscillospira* [42,55,58]; *Oribacterium* [58]; *Centipeda* [48,58]; *Bulleidia* [42]; *Phascolarctobacterium* [62]; *Bacillus* [52]; *Faecalibacterium* [62]; *Megasphaera* [48,55,58]; *Megamonas* [62]; *Roseburia* [42,55,58]; *Stomatobaculum* [48,52,58]; *Dorea* [58,62]; *Selenomonas* [48,58]; *Clostridium* [62]; *Lactobacillus* [42,52,58,62]; *Blautia* [62]; *Gemmiger* [42,55,58]; *Actinomyces* [45,48,58]; *Rothia* [42,48,52,58,62]; *Scardovia* [48,58]; *Slackia* [52]; *Campylobacter* [48,58]; *Neisseria* [55,58,62]; *Haemophilus* [48,52,58,62]; *Lautropia* [48,58]; *Kingella* [58]; *Eiknella* [48,58]; *Actinobacillus* [58]; *Escherichia* [42,55,58,62]; *Aggregatibacter* [55]; *Salmonella* [55]; *Capnocytophaga* [45,48,55,58]; *Prevotella* [48,52,55,58,62]; *Porphyromonas* [42,55,58]; *Bacteroides* [42,58,62]; *Alloprevotella* [48,55,58,62]; *Paludibacter* [58]; *Tannerella* [58]; *Alistipes* [62]; *Treponema* [48,58]; *Spirochaeteles* [52]; *Mycoplasma* [48,58]; *Mollicutes* [52]; *Cloacibacillus* [42,55,58].

**Figure 6:**
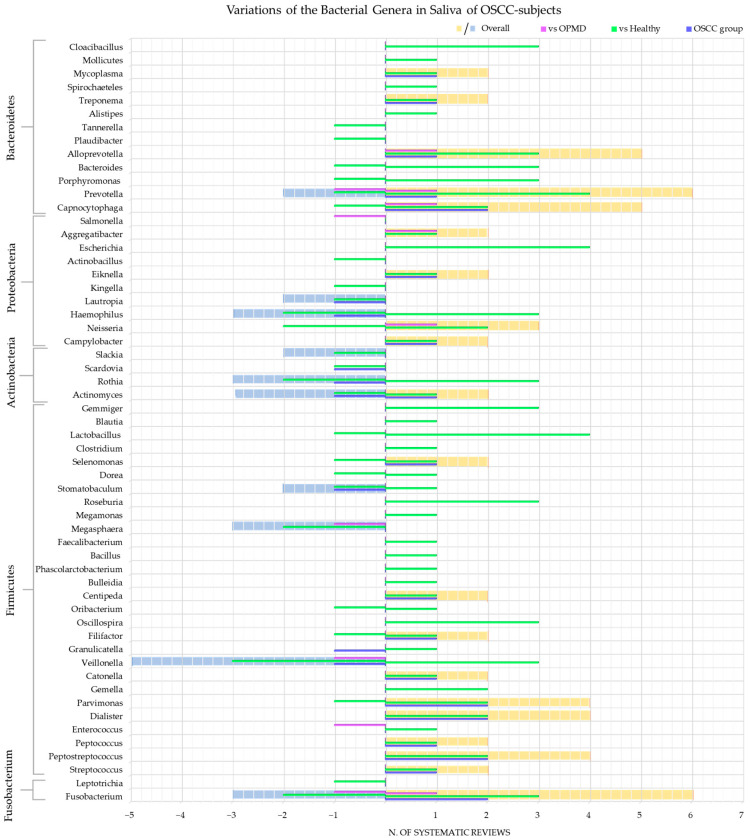
Systematic reviews that reported any variations in the bacterial genera content in the OSCC-tissue samples of adult subjects, the comparison with the healthy and OPMD control group, and the final number of systematic reviews that reported an increase (positive number) or decrease (negative number) for each bacterial genus. Notes: studies investigating variations in different groups were counted separately for each group.

Figure 6 shows the number of systematic reviews that reported any variations in the bacterial genera content in the saliva of adult subjects with OSCC, the comparison with the healthy and OPMD control group, and the final number of systematic reviews that reported an increase or decrease for each bacterial genus.

The species and subspecies of bacteria detected were: *F. nucleatum* [58], *F. periodonticum* [58]; *S. pneumoniae* [48], *S. mitis* [52], *S. costellatus* [58], *S. anginosus* [58]; *Peptostreptococcus anaerobius* [58]; *Parvimonas micra* [58]; *Catonella morbi* [55]; *Veillonella parvula* [58], *Veillonella dispar* [58]; *Filifactor alocis* [58]; *Selenomonas noxia* [58]; *Lactobacillus plantarum* [58]; *Rominococcus gnavus* [58]; *Aeroglobus geminatus* [55]; *Actinomyces odontolyticus* [58], *Actinomyces oral taxon_170* [58]; *Rothia mugilaginosa* [58], *Rothia aeria* [58], *Rothia dentocariosa* [58]; *C. ureolyticus* [58]; *Neisseria subflava* [58], *Neisseria bacilliformis* [58]; *Haemophilus influenzae* [58], *Haemophilus parainfluenzae* [58]; *Aggregatibacter segnis* [58]; *Helicobacter pylori* [43,47]; *Ralstonia insidiosa* [42]; *Stenotrophomonas ruminococcus* [58]; *Capnocytophaga ochracea* [58], *Capnocytophaga sputigena* [55]; *Prevotella (P.) intermedia* [58], *P. tannerae* [58], *P. olorum* [55], *P. pallens* [58], *P. melaninogenica* [58], *P. nigrescens* [58], *P. nanceiensis* [58], *P. corpi* [58]; *Porphyromonas gingivalis* [55,58], *Porphyromonas pasteri* [58], *Porphyromonas endodontalis* [58]; *Bacteroides ovatus* [58]; *Parabacteroides distasonis* [58]; *Tannarella forsythia* [58].

None of the remaining extracted records were reported.

**Figure 7:**
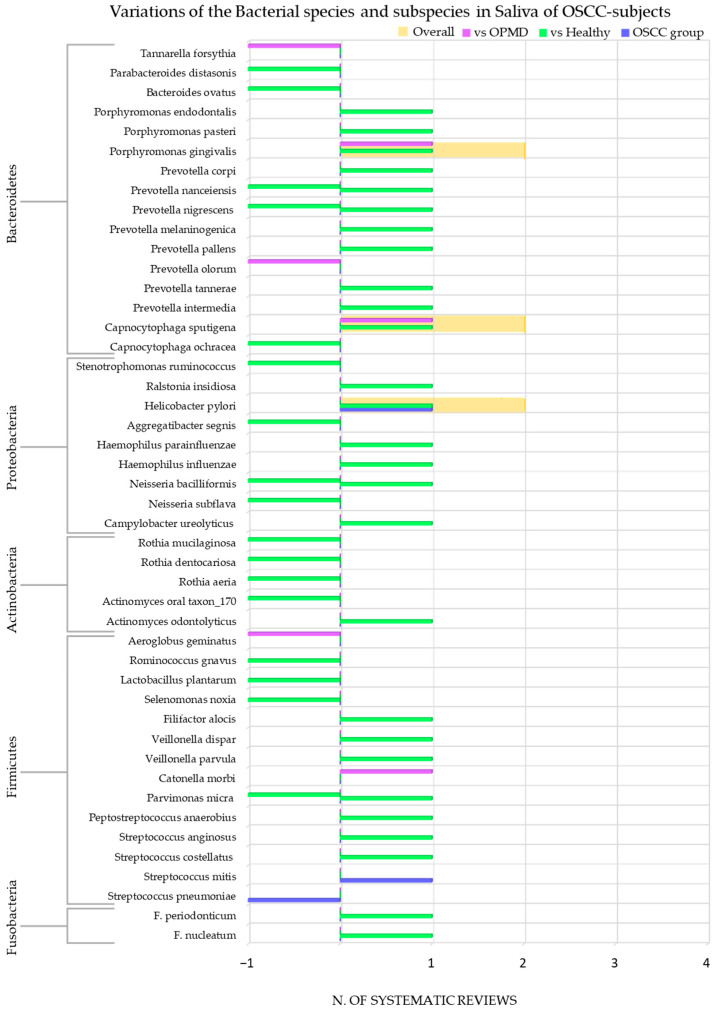
Systematic reviews that reported any variations in the bacterial species/subspecies content in the OSCC-tissue samples of adult subjects, the comparison with the healthy and OPMD control group, and the final number of systematic reviews that reported an increase (positive number) or decrease (negative number) for each bacterial genus. Notes: studies investigating variations in different groups were counted separately for each group.

Figure 7 shows the number of systematic reviews that reported any variations in the bacterial species/subspecies content in the saliva of adult subjects with OSCC, the comparison with the healthy and OPMD control group, and the final number of systematic reviews that reported an increase or decrease for each bacterial genus.

### 3.4. Oral Viral Content in Adult Subjects with OSCC: Study Characteristics and Qualitative Synthesis

Twenty-two studies [37,38,39,40,41,44,46,47,49,50,51,53,54,56,57,59,60,61,63,64,65,66] described the oral viral content in adult subjects with OSCC, in particular, two studies [47,54] of the OSCC-tissue samples and saliva, twenty studies [37,38,39,40,41,44,46,49,50,51,56,57,59,60,61,63,65,66] only in the OSCC-tissue samples, and two studies [53,64] only of the saliva.

#### 3.4.1. Oral Viral Content in OSCC-Tissue Samples of Adult Subjects

Twenty studies [37,38,39,40,41,44,46,47,49,50,51,54,56,57,59,60,61,63,65,66] described the viral content of the OSCC-tissue samples of adult subjects (Table 2).

Sixteen studies [38,39,41,44,46,50,51,54,56,57,59,60,61,63,65,66] reported a sample size of the OSCC group amounting to 24.847, ten studies [37,41,44,50,51,56,57,59,63,66] involved a healthy control group evaluating 9.573 cases, and two studies [41,50] a group with OPMD of 223 subjects.

The gender ratio was reported by three studies [38,39,51], amounting to 3.328 males and 1.265 females (2.6:1), while the mean age was reported by one study [54] (56.80 years old) and the age range by two studies [39,54] (from 19 to 94 years).

The reported country of origin of the samples was India (n = 3.741) [40,41,46,51,54,56,60], China (n > 3.242) [39,40,46,54,56,57,60,63,65], the United States of America (n > 2.109) [39,40,41,46,56,60], Japan (n = 1.941) [40,46,54,56,60], Australia (n > 1522) [46,54], Taiwan (n > 667) [40,46,54,60], South Africa (n > 409) [40,56,60], Korea (n = 285) [46,54,60], Sweden (n > 180) [41,46,56,60], the Netherlands (n = 178) [40,46,56,60], Venezuela (n > 166) [40,46,60], Greece (n = 153) [39,60], Hungary (n > 144) [40,56,60], Finland (n > 119) [40,46,60], Slovenia (n = 117) [46,60], Sri Lanka (n = 116) [41,54], Malaysia (n = 109) [54], Sudan (n > 108) [40,41,46,60], Italy (n > 104) [40,46,60], Chile (n = 80) [39], Brazil (n > 69) [40,60], United Kingdom (n = 59) [41,46], Poland (n > 53) [41,46], Canada (n = 53) [46], Germany (n > 53) [40,46], Spain (n > 49) [40,46,56,60], Pakistan (n = 48) [54], Bangladesh (n = 34) [54], Thailand (n = 32) [54], Hong Kong (n = 31) [54], Egypt (n = 22) [56], Norway (n > 20) [46], Switzerland (n = 15) [46], Yemen (n = 18) [41], France (n = 12) [46], Argentina [40], Czech Republic [40], North Ireland [46], Serbia [37], and Cuba [46] (number not defined).

As risk factors for OSCC, two studies [39,54] specified the use of smoked tobacco or alcohol, one study [54] reported the use of smokeless tobacco, and another study [40] reported the concomitant use of tobacco and alcohol.

The OSCC was localized to the palatine tonsil in more than 1.820 cases [41,54,66], the tongue in more than 930 [37,39,40,41,51,54], buccal mucosa in more than 460 [37,41,54], gingiva in 196 [54], hard or soft palate in 186 [41,54], lip in 125 [41,54], the oral floor in more than 126 [37,41,54], and dentoalveolar complex in more than 1 [37,39]. The location of the OSCC was not specified in the remaining cases.

Three studies [37,40,61] reported the staging of the OSCC as follows: in situ OSCC (n = 7) [40]; stage I (n = 134) [37]; stage II (n = 185) [37,40]; stage III (n = 172) [37]; stage IV (n = 551) [37]; stage III–IV (n = 333) [61]; stage I–IV (n = 327) [61].

The method of sample collection most used was a biopsy (n > 16.820) [37,39,41,44,46,47,49,50,54,56,57,59,60,61,65,66], followed by serum analysis (n > 1.551) [37,66], brush (n > 788) [37,47,50,59], blood analysis (n = 50) [50], biofilm sampling (n = 21) [50], and swab (number not defined) [47].

The microorganism identification technique included: PCR (n > 20.503) [38,40,41,46,47,49,50,51,54,56,57,59,60,61,63,65]; nested PCR (n = 471) [40,50,54,56]; southern blot PCR (n = 224) [54]; in situ PCR (n = 220) [40]; PCR dot blot (n = 198) [40]; differential PCR (n = 60) [40]; RT-PCR (n = 59) [40]; RT-qPCR (n = 21) [56]; slot blot PCR (n = 15) [54]; ISH (n = 1.819) [38,40,41,47,51,54,56,57,59,60,61,65]; radioactive ISH (n = 117) [40]; dot blot hybridization (n = 66) [63]; in situ PCR and ISH (n = 20) [54]; PCR and ISH (n = 244) [54]; PCR or ISH (n = 232) [51,60]; ISH or immunofluorescence or immunoperoxidase (n = 608) [49]; IHC (n > 1.538) [38,40,47,51,56,57,65]; PCR or p16-IHC (n = 216) [51]; RT-PCR and IHC (n = 52) [54]; ELISA (n = 132) [50]; southern blot or dot blot or filter blot hybridization (n = 321) [49]; DNA sequencing (n = 64) [40]; single strand conformation polymorphism (n = 60) [40]; PAP technique (n = 50) [50].

The sourced target through the microorganisms identification technique was: HPV DNA (n > 2.723) [37,40,49,54]; p53 (n = 496) [40]; p16 (n = 193) [40]; p21 (n = 33) [40]; pRb (n = 112) [40]; E6 and E7 mRNA (number not defined) [39].

HPV was found in the OSCC-tissue samples of adult subjects in thirteen studies [38,39,40,41,47,49,51,54,59,60,61,65,66], and in eleven of them [38,40,47,49,51,54,59,60,61,65,66], the association between HPV and OSCC were significant, while in two studies [39,41], they were not significant. Nine studies [38,39,51,54,59,60,61,65,66] reported the total number of OSCC groups and the number of positive cases for HPV, amounting to 5.151 of 19.009 (27.10%). Three other studies [40,41,49] registered the number of positive HPV-OSCC-tissue samples (n = 1.213), totaling 6.364 positive HPV-OSCC tissue samples. One study [47] did not record the total and the positive numbers.

Three studies [37,44,63] investigated the prevalence of HPV in the OSCC-tissue samples in comparison with a healthy control group, and in all three studies [37,44,63], the prevalence was higher in the OSCC group. Two studies [44,63] reported the total number of OSCC groups and the number of positive cases for HPV, amounting to 374 of 679 (55.08%).

One study [46] investigated the prevalence of HPV in the OSCC-tissue samples in comparison with an oropharyngeal or laryngeal control group, and the prevalence was lower in the OSCC group.

The HPV genotypes were specified in eleven studies [38,39,40,44,46,47,49,54,59,60,63], and were: HPV-16 in 1.200 of the 3.119 positive cases (38.47%) [38,39,44,46,49,59,63] investigated for that HPV genotype; HPV-18 in 313 of 1.45 (21.45%) [39,44,46,49]; HPV-2 in 2 of 627 (0.48%) [49]; HPV-3 in 1 of 627 (0.16%) [49]; HPV-4 in 2 of 627 (0.32%); HPV-6 in 111 of 1.423 (7.80%) [46,49]; HPV-10 in 16 of 627 (2.55%) [49]; HPV-11 in 68 of 1.423 (4.78%) [46,49]; HPV-13 in 1 of 627 (0.16%) [49]; HPV-31 in 6 of 1.423 (0.42%) [46,49]; HPV-32 in 1 of 796 (0.13%) [46]; HPV-33 in 23 of 1.423 (1.62%) [46,49]; HPV-35 in 1 of 796 (0.13%) [46]; HPV-39 in 0 of 796 (0.0%) [46]; HPV-44 in 1 of 796 (0.13%) [46]; HPV-45 in 0 of 796 (0.0%) [46]; HPV-51 in 0 of 796 (0.0%) [46]; HPV-52 in 0 of 796 (0.0%) [46]; HPV-53 in 1 of 796 (0.13%) [46]; HPV-56 in 2 of 796 (0.25%) [46]; HPV-57 in 2 of 1423 (0.14%) [46,49]; HPV-58 in 1 of 796 (0.13%) [46]; HPV-59 in 0 of 796 (0.0%) [46]; HPV-68 in 1 of 796 (0.13%) [46]; HPV-73 in 0 of 796 (0.0%) [46]; HPV-81 in 1 of 796 (0.13%) [46]; HPV-82 in 0 of 796 (0.0%) [46]; HPV-16 and -18 co-infection in 93 of 1.423 (6.54%) [46,49]; HPV-6 and -11 co-infection in 27 of 627 (4.31%) [49]; HPV-6, -11 and -16 co-infection in 3 of 627 (0.48%) [49]; HPV-31, -33 and -35 co-infection in 6 of 627 (0.96%) [49]. Five other studies [38,40,47,54,60] reported the HPV genotypes detected, but the number of positive cases was not specified.

**Figure 8:**
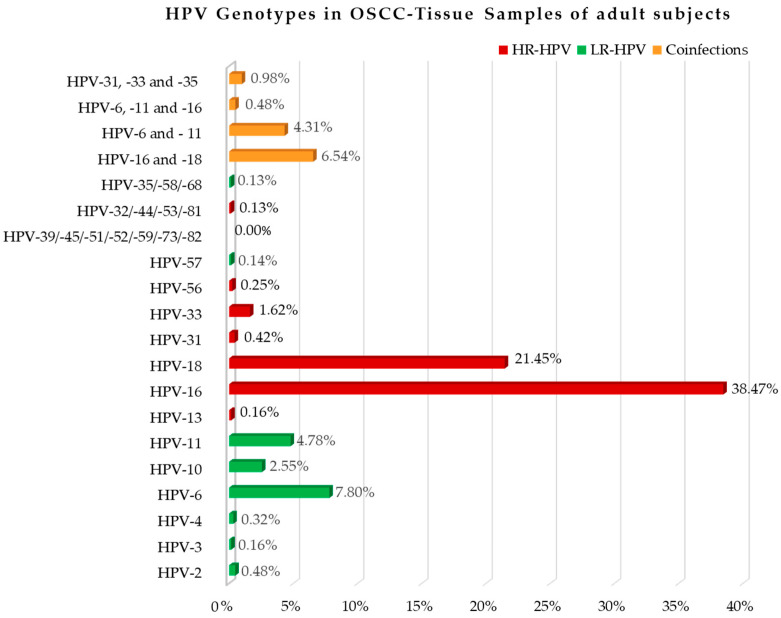
Percentage frequency of HPV genotypes detected in the OSCC tissue samples of adult subjects.

Figure 8 shows the percentage frequency of the HPV genotypes found in the OSCC tissue samples of adult subjects.

Six studies [40,47,50,56,57,64] evaluated the presence of EBV in the OSCC tissue samples. Four of them [40,50,56,64] recorded an increase in the virus in the OSCC group, one study [57] had an increase in the OSCC group compared to the healthy control group, and for one study [47], the association was not significant. Three studies [56,57,64] reported that the total number of cases in the OSCC group amounted to 3.700 cases, of which 1.700 (45.95%) were positive for EBV. Another study [40] registered the number of positive EBV-OSCC tissue samples (n = 236), totaling 1.936 OSCC tissue samples positive for EBV recorded in the present umbrella review.

One study [41] evaluated the presence of EBV and HPV co-infection in 1.109 OSCC-tissue samples, and in 95 cases (5.86%), the co-infection was present, and the association was statistically significant.

One study [47] investigated the presence of Human Herpes Virus type 6 (HHV-6) in the OSCC tissue samples, and the association was not significant.

Another study [50] reported a nonsignificant association between the Herpes Simplex Virus type 1 (HSV-1) and the OSCC.

None of the remaining extracted records were reported.

**Figure 9:**
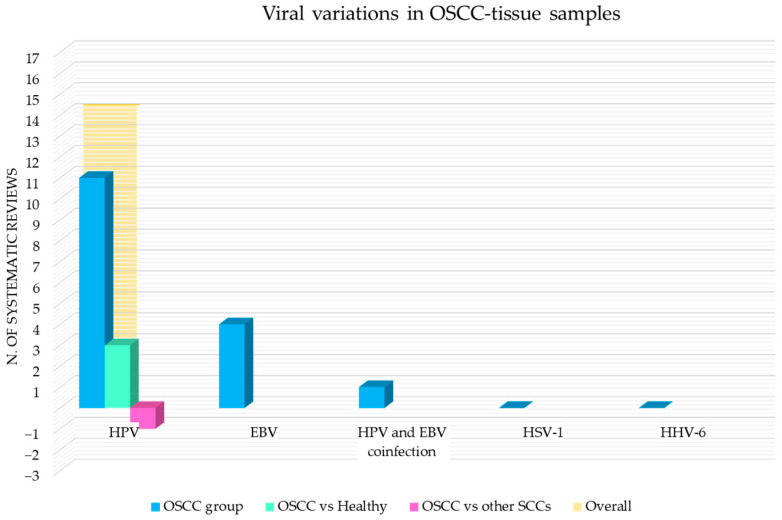
Systematic reviews that reported any variations in the viral content in the OSCC tissue samples of adult subjects, the comparison with the healthy and the control group with other squamous cell carcinomas, and the final number of systematic reviews that reported an increase (positive number) or decrease (negative number) for each viral species. Notes: studies investigating variations in different groups were counted separately for each group.

Figure 9 shows the number of systematic reviews that reported any variations in the viral content in OSCC tissue samples of adult subjects, the comparison with the healthy and the control group with other squamous cell carcinomas (oropharyngeal and laryngeal squamous cell carcinoma), and the final number of systematic reviews that reported an increase or decrease for each viral species.

#### 3.4.2. Oral Viral Content in Saliva of Adult Subjects with OSCC

Four studies [47,53,54,64] described the viral content of the saliva in adult subjects with OSCC (Table 5).

Three studies [53,54,64] reported an OSCC group sample size of 704 and 2653 for the healthy control group. One study [64] involved a group with 12 cases of OPMD.

The reported country of origin of the samples was India for 347 OSCC cases [53,54], the United States of America for 109 [53], Sweden for 85 [53], Canada for 72 [53], Pakistan for 35 [53], Iran for 22 [53], and France for 22 [53].

The method of sample collection most used was a saliva test (n > 418) [47,53,54,64], followed by an oral rinse (n = 286) [53] and a swab (number not defined) [47].

The microorganisms identification technique included PCR (n > 419) [47,53,54]; nested PCR (n = 56) [53,64]; quantitative PCR (n = 144) [53]; nested PCR and DNA sequencing (n = 85) [53]; culture, immunohistochemistry, gas chromatography, spectrophotometer, VIDAS EBV kit or QIAamp Mini Elute Virus Spin kit Digene HPV genotyping RH test (number not defined) [47].

The sourced target through the microorganisms identification technique was reported by three studies [53,64], and it was the EBV genome (n = 12) [64], HPV genome (n = 1440) [53,54], and in particular, the HPV genome of any HR-HPV (n = 538) [53], of any LR-HPV (n = 107) [53], of HPV-16 (n = 507) [53], of HPV-18 (n = 254), and [53] and it was not specified in 34 cases [54].

HPV was found in the saliva of adult subjects with OSCC in three studies [47,53,54] totaling more than 295 OSCC cases. In particular, one study [53] reported the HPV positivity for 54 cases of HPV-16 genotypes (statistically significant), 24 cases of HPV-18 (not significant), 164 cases of other HR-HPV (statistically significant), and 8 cases of LR-HPV (not significant). The association between HPV and OSCC was reported as statistically significant in two studies [53,54]. One study [47] registered fewer HPV-16 and -18 positive OSCC cases compared to the healthy control group, and the association between OSCC and HPV-18 was not significant.

EBV was found in the saliva of adult subjects with OSCC in two studies [47,64], totaling more than 7 OSCC cases. Both studies [47,64] reported that the number of EBV positives was higher in the case group than in the healthy control group. One study [64] established the same augmentation in comparison with the OPMD group. The association between EBV and OSCC was not statistically significant for either study [47,64].

None of the remaining extracted records were reported.

**Figure 10:**
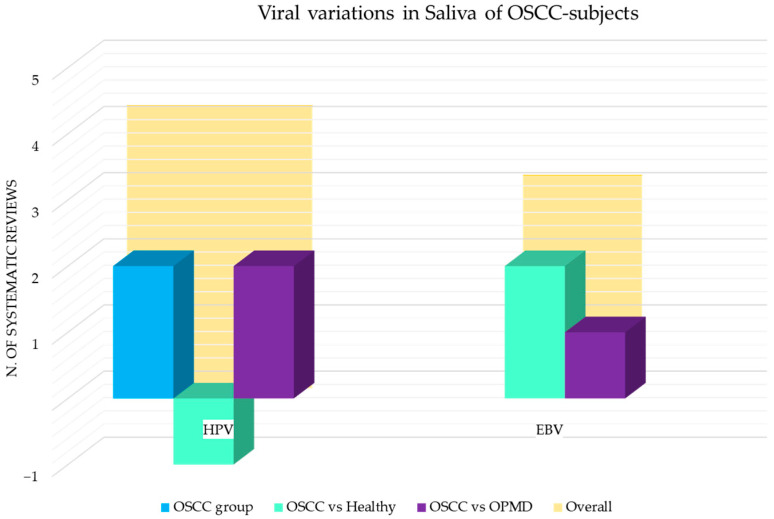
Systematic reviews that reported any variations in the viral content in the saliva of adult subjects with OSCC, the comparison with the healthy and the OPMD control group, and the final number of systematic reviews that reported an increase (positive number) or decrease (negative number) for each viral species. Notes: studies investigating variations in different groups were counted separately for each group.

Figure 10 shows the number of systematic reviews that reported any variations in the viral content in the saliva of adult subjects with OSCC, the comparison with the healthy and the OPMD control group, and the final number of systematic reviews that reported an increase or decrease for each viral species.

### 3.5. Oral Fungal Content in Adult Subjects with OSCC: Study Characteristics and Qualitative Synthesis

Three studies [35,47,55] described the oral fungal content in adult subjects with OSCC, in particular, two studies [35,47] of the OSCC tissue samples and saliva, and one study [55] only of saliva.

#### 3.5.1. Oral Fungal Content in the OSCC Tissue Samples of Adult Subjects

Two studies [35,47] described the fungal composition in the OSCC tissue samples of adult subjects (Table 3).

The sample size of the OSCC group was reported in one study [35], totaling 136 and 107 for the OPMD group, while the number of the healthy control group was not recorded.

One study [35] registered the country of origin of the sample: India (n = 80), Egypt (n = 31), Argentina (n = 25), and Taiwan (number not defined).

The method of sample collection most used was biopsy (n > 61) [35,47], followed by the swab (n > 50) [35,47], both swab and biopsy (n > 25) [35], and brush (number not defined) [47].

The microorganisms identification technique included culture (n > 136) [35,47], immunohistochemistry [35,47], PCR [47], or in situ hybridization [47] (number not defined).

One study [47] reported an increase in oral yeast in the OSCC tissue samples, and in particular of *Candida* in the adult subjects who had undergone chemotherapy or radiotherapy. The other study [35] registered an increase in *Candida,* comparing the healthy and the OPMD control groups.

**Figure 11:**
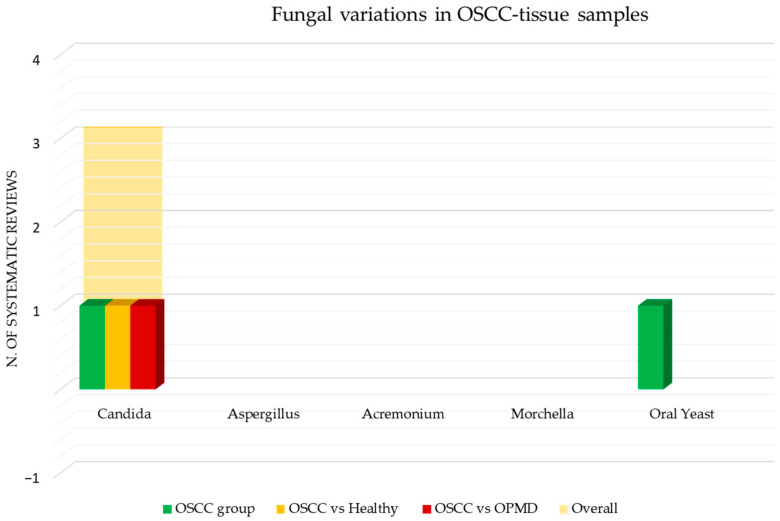
Systematic reviews that reported any variations in the fungal content in the OSCC tissue samples of adult subjects with OSCC, the comparison with the healthy and the OPMD control group, and the final number of systematic reviews that reported an increase (positive number) or decrease (negative number) for each fungal species. Notes: studies investigating variations in different groups were counted separately for each group.

None of the remaining extracted records were reported in the two studies [35,47]. Figure 11 shows the number of the systematic reviews that reported any variations in the fungal content in OSCC-tissue samples of adult subjects with OSCC, the comparison with the healthy and the OPMD control group, and the final number of systematic reviews that reported an increase or decrease for each fungal species.

#### 3.5.2. Oral Fungal Content in Saliva in Adult Subjects with OSCC

Three studies [35,47,55] described the fungal content of the saliva of adult subjects with OSCC (Table 6).

Two studies [47,55] reported a sample size of the OSCC group amounting to 531. There were 627 in the healthy control group and 405 in the OPMD group. One study [35] involved a group with other malignancies, evaluating 6 cases.

The gender ratio was specified for 27 OSCC cases, and they were all males [55].

The reported country of origin of the samples was Australia for 104 OSCC cases [35], India for 97 [55], Finland for 100 [35], India for 31 [35], the United States of America for 18 [55], China for more than 29 [55], and Taiwan but the number was not specified [55].

The method of sample collection most used was a saliva test (n > 349) [35,47,55], followed by an oral rinse (n = 154) [35,55], oral, plaque swab, and saliva test (n = 29) [55], and oral swab (number not defined) [47].

The microorganisms identification technique was culture (n > 301) [35,47]; reverse transcription PCR (n = 52) [35]; QIAamp DNA blood mini kit (n = 124) for DNA extraction [55]; HiPure tissue and blood DNA kit (n = 32) [55]; QIAamp DNA mini kit (n = 21) [55]; modified QUAGEN DNA (n = 18); Gene Fix Saliva Prep 2 isolation kit (n = 31) [55]; VIDAS EBV kit, QIAamp Mini Elute Virus Spin kit Digene HPV genotyping RH test, spectrophotometer, gas chromotherapy, immunohistochemistry or PCR (number not defined) [47].

*Candida* was increased in the saliva in two studies [35,47] compared to the healthy control group and the OPMD group [35,55].

*Aspergillus* and *Acremonium* were increased in one study [55] compared to the OPMD group.

*Morchella* was decreased in one study [55] compared to the OPMD group.

None of the remaining extracted records were reported in the three studies [35,47,55].

**Figure 12:**
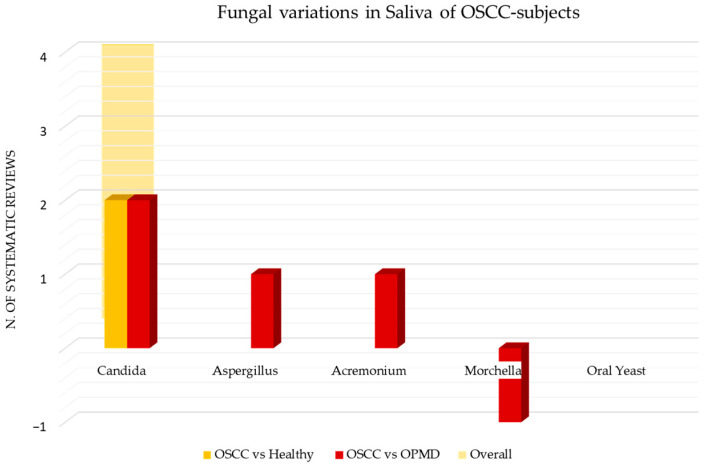
Systematic reviews that reported any variations in the fungal content in the saliva of adult subjects with OSCC, the comparison with the healthy and the OPMD control group, and the final number of systematic reviews that reported an increase (positive number) or decrease (negative number) for each fungal species. Notes: studies investigating variations in different groups were counted separately for each group.

Figure 12 shows the number of the systematic reviews that reported any variations in the fungal content in the saliva of adult subjects with OSCC, the comparison with the healthy and the OPMD control group, and the final number of systematic reviews that reported an increase or decrease for each fungal species.

### 3.6. Quality Assessment

Three studies [53,55,58] were judged as high quality, five [36,47,56,61,62] as moderate, six [39,42,43,45,54,57] as low, and eighteen [35,37,38,40,41,44,46,48,49,50,51,52,59,60,63,64,65,66] as critically low quality through the use of the AMSTAR-2 tool, as reported in Table 1, Table 2, Table 3, Table 4, Table 5 and Table 6 and illustrated in Supplementary File S3.

## 4. Discussion

The present umbrella review aimed primarily to evaluate the content of oral microorganisms (bacteria, viruses, and fungi) in OSCC tissue and saliva samples from adult subjects (>18 years) with OSCC and secondarily to compare the findings with those of non-OSCC patients.

Thirty-two systematic reviews with or without meta-analysis were included in the present study, eleven of which described oral bacteria content, twenty-two recorded viral content, and only three reported fungal content in the OSCC tissue samples or saliva from adult OSCC patients.

### 4.1. Bacterial Content of OSCC-Tissue Samples or Saliva in Adult Subjects with OSCC

Eleven studies examined bacterial levels in the saliva or neoplastic tissue samples from 6216 OSCC patients.

The sample collection and bacterial identification methods most commonly used were biopsies and polymerase chain reaction (PCR) in tissue samples and saliva testing and next-generation sequencing (NGS)-based diagnostic approaches that allow detection of any nucleic acid significantly represented in a sample (e.g., QIAamp DNA Blood Mini Kit, QIAamp DNA Microbiome Kit, MiSeq) for DNA extraction [67].

Despite technological advances, only about 57% of oral bacterial species have been identified, 13% have been cultured but have yet to be named, and 30% were not yet isolatable or replicable in biological cultures, as reported by the Human Oral Microbiome Database [48].

#### 4.1.1. Fusobacteria Phylum in Tissue and Saliva Samples of Adult Subjects with OSCC

Although two studies reported a decreased occurrence of *Fusobacteria* phyla in three OSCC tissue samples, an increase in three *Fusobacteria* phyla was described in seven studies, reporting positivity in nine neoplastic tissue samples, and four studies, registering positivity for six saliva samples from adult OSCC patients. However, in agreement with the results of Li et al. [18], *Fusobacterium* was the oral bacterium that changed the most in OSCC patients.

Specifically, *Fusobacterium* was particularly elevated in stage IV OSCC patients [68,69]. However, the results of this umbrella review do not allow us to correlate the increase in *Fusobacterium* with a specific tumor stage, as staging was not reported in almost any of the included systematic reviews. Nevertheless, the present results show that Fusobacterium generally increased in both saliva and tissue samples from the OSCC adult subjects, first compared with the healthy group (7-fold increase) and then compared with the group with OPMD (2-fold increase).

Indeed, the content of *Fusobacteria*, especially *F. nucleatum*, in the oral cavity of healthy subjects is low and is presumed to increase in OPMD and even more in OSCC [18,36]. This finding could be supported by the “drive passages” hypothesis, which states that oral bacteria have carcinogenic potential, damaging DNA and thus promoting the development of oral cancer [18]. Based on these properties, drive passengers should be more common during dysplasia or in the early stages of OSCC [18]. Accordingly, *F. nucleatum* has been identified as a drive-passenger capable of promoting tumor cell proliferation and metastasis [18]. Specifically, *Fusobacterium* may play a dominant role over other oral bacteria due to its ability to coaggregate with other species and form an egocentric network in patients with OSCC [18,70].

#### 4.1.2. Firmicutes Phylum in Tissue and Saliva Samples of Adult Subjects with OSCC

Six studies in the present umbrella review reported an increased content of Firmicutes phylum. Conversely, *Firmicutes* phylum was reduced in nine studies, similar to previous studies reporting a decrease in OSCC compared to healthy and OPMD subjects [48,62].

In particular, *Peptostreptococcus*, *Parvimonas*, *Dialister*, *Veillonella*, *Oscillospira*, *Roseburia*, *Lactobacillus*, and *Gemmiger* are the genera of the *Firmicutes* phylum that were most elevated in the saliva of adult patients with OSCC, contrary to the genus *Veillonella*, which was most reduced compared to the healthy group. *Peptrostreptococcus* and *Parvimonas* were also elevated in the OSCC tissue samples, as were *Gemella* and *Streptococcus.* However, the reported results for *Streptococcus* were contradictory, as the bacterium was also frequently found at lower levels than in the healthy control group.

Similar to the present results, the depletion of *Streptococcus* in neoplastic tissue samples from adult OSCC patients was described by Li et al. [18] and probably, when accompanied by enrichment of *Fusobacterium*, should be considered a predictive signal for the development of OSCC [62]. This hypothesis is based on the evidence that the genus *Streptococcus* genus has been identified as one of the predominant bacterial genera in the oral cavity of healthy subjects [62] and that several *Streptococcus* species (e.g., *S. salivarius* and *S. cistatus*) can downregulate inflammatory processes by inhibiting the synthesis of proinflammatory interleukins [71], and impair the proinflammatory effects of *F. nucleatum* [36,62]. This supports the hypothesis that the *Fusobacterium* abundance observed in the present study may be correlated with streptococcal deficiency in patients with OSCC, who were found to have a chronic inflammatory response that was also supported by oral dysbiosis.

#### 4.1.3. Actinobacteria Phylum in Tissue and Saliva Samples of Adult Subjects with OSCC

Six studies (three describing findings from neoplastic tissue and three from saliva samples) reported a decreased content of the *Actinobacteria* phylum, while only one study reported an increase in *Actinobacteria* in saliva.

Notably, the genus *Rothia* was the one that decreased the most [72]. However, in the study by Yang et al., *Rothia* increased in patients with OSCC of the lining mucosa, gingiva, and tongue [72]. Secretion of peptidases in tumor tissue by *Rothia* leads to degradation of the extracellular matrix and modulation of the host immune response, accompanied by destruction of the physical barriers. Therefore, the oral cavity region affected by OSCC may also affect the existing bacterial profile, which also affects the bacterial microenvironment present under healthy oral conditions or in the presence of other oral diseases [73].

In contrast, opposite results were found for the *Actinomyces* content, which increased in four studies and decreased in three, consistent with several studies that reported a decrease in *Actinobacteria* [18,62,74].

Seven studies (four on OSCC tissue samples and three on saliva) under consideration indicated an overall increase in the *Proteobacteria* phylum content. In contrast, only one study reported a decrease in *Proteobacteria* in saliva.

Specifically, *Neisseria* and *Campylobacter* were most frequently elevated (six and five times, respectively), followed by *Escherichia* and *Helicobacter pylori* (four times each), and *Haemophilus* and *Aggregatibacter* (three and two times, respectively).

Muthusamy et al. [50] showed that *Neisseria* increased the risk of OSCC by 0.75 times. Most of the role of *Neisseria* in oral carcinogenesis is related to its strong ability to metabolize alcohol into acetaldehyde, a known carcinogen [50,71].

Poor oral hygiene was associated with an increased risk of esophageal cancer, in whose tissue samples Poosari et al. [75] found a statistically significant increase in *Campylobacter* prevalence. In particular, the combination of poor oral hygiene practices and co-infection with *C. rectus* and *C. concisus* were significantly associated with esophageal cancer [75]. In fact, *Campylobacter*, with *Fusobacterium* and *Porphyromonas*, is part of the so-called “oral mobile microbiome” because, despite starting in the oral cavity, it is associated with many inflammatory-infectious manifestations in other distant regions [76].

The role of *Escherichia* in oral carcinogenesis is not known. Instead, it is known for colorectal cancer. *Escherichia coli* is able to induce the production of cytokines, chemokines, and free radicals that create a pro-inflammatory environment [77]. In addition, some pathogenic *Escherichia coli* can produce various toxins: cyclomedelins, such as cytotoxic necrotizing factor, cycle inhibiting factor, colibactin, and cytolethal distending toxins [78]. Cytomedelins affect differentiation, proliferation, and cell death by interfering with the cell cycle and DNA repair mechanisms [78].

*Helicobacter pylori* is the main risk factor for gastric cancer. A link between *Helicobacter pylori* and the upper airway or oral cavity diseases was hypothesized on the basis of anatomical continuity, but the possible role of the bacterium in oral carcinogenesis is still unclear [79]. Grimm et al. [80] showed an increased expression of Toll-Like Receptor (TLR) 5 in OSCC correlated with an increased presence of *Helicobacter pylori*. TLR-5 was associated with an increased presence of bacterial flagella that would be responsible for the migration and proliferation of cancer cells in cervical cancer and non-small cell lung cancer [80]. However, the study by Gupta et al. [43] recorded no association between TLR-5 and OSCC progression.

Fernando et al. [81] showed that in betel chewers, there was an increased prevalence of *Helicobacter Pylori* compared to non-chewers, both in the OSCC and in the healthy group. The authors hypothesized that areca nut extracts, a recognized risk factor for OSCC, were able to affect the periodontal bacterial microenvironment regardless of the presence or absence of OSCC [81].

#### 4.1.4. Bacteroidetes Phylum in Tissue and Saliva Samples of Adult Subjects with OSCC

The *Bacteroidetes* phylum was reported to be increased in eight studies (two in neoplastic tissue and six in saliva samples from OSCC patients); in comparison, it decreased in the other four studies considered. The greatest increase was reported in saliva samples and, to a lesser extent, in neoplastic tissue ones from OSCC subjects. The genera of *Capnocytophaga* and *Prevotella* were the most frequently increased (nine times for both bacteria), followed by *Porphyromonas* and *Alloprevotella* (five times for both bacteria), and *Bacteroides* and *Treponema* (three and two times respectively).

According to the study by La Rosa et al. [82], *Capnocytophaga* is the most increased genus of the phylum *Bacteroidetes*. However, the same study identified *Capnocytophaga gingivalis* as the species most associated with cancer [82], whereas the results of the present study identified *Capnocytophaga sputigena* as the most increased in saliva. None of the systematic reviews included in the present study investigated the presence of *Capnocytophaga gingivalis,* and the study by Yost et al. [83] showed that this bacterium was the most represented in non-tumor sites.

Esophageal, gastric, and pancreatic cancer was associated with *Prevotella intermedia* [3]. In relation to the oral cavity, the species *Prevotella pallens* was significantly correlated with the risk of distant metastases [3]. Furthermore, the study by Arthur et al. [84] observed increased *Prevotella* abundance, especially in more advanced OSCC, and the increased presence of certain bacteria species, such as *Prevotella tannerae* and *Prevotella intermedia* in saliva, was associated with a two-fold increased risk of developing OSCC [3]. *Prevotella intermedia*, similar to Porphyromonas gingivalis, have been observed to release peptides, augmented at tumor sites, and specifically proteases, that may function as signaling molecules, engaging proteinase-activated receptors (PARs). Activation of these receptors has been associated with diverse cellular processes such as proliferation, apoptosis, autoimmunity (Lisi et al., 2014), cytokine generation, local tissue inflammation, pain perception, and modulation of epithelial barrier functionality (Amadesi and Bunnett, 2004). In addition, these proteases have the potential to degrade the host’s extracellular matrix, compromise physical barriers, and influence the host’s immune response, ultimately contributing to cancer genesis and progression.

Also, *Porphyromonas gingivalis* was associated with esophageal cancer, and *Tannerella forsythia* with esophageal adenocarcinoma [85]. In particular, *Porphyromonas gingivalis* was linked to lymph node metastases and a worsened prognosis in subjects with esophageal cancer [85]. The study of Li et al. [18] hypothesized that *Porphyromonas gingivalis* might be a drive-passenger, like *F. nucleatum*. Indeed, the abundance of *Porphyromonas gingivalis* in subjects with OSCC was recorded mainly in the early stages of oral cancer [69]. The results of the present study showed an increase of *Porphyromonas,* principally in the saliva of subjects with OSCC, compared to the healthy control group and the OPMD group.

#### 4.1.5. Periodontal Pathogens and Oral Carcinogenesis

The present study showed that periodontal pathogens are among the bacteria that undergo the most remarkable changes in oral dysbiosis associated with OSCC. Three mechanisms were hypothesized to explain the possible involvement of periodontal pathogenic bacteria in oral carcinogenesis: indirect induction of chronic inflammation, production of carcinogenic metabolites, and direct anti-apoptotic effect [48,86,87].

Periodontal bacteria, such as *Fusobacterium* and *Prevotella*, produce various cytokines, interleukins, growth factors, and metalloproteinases [48,68,88,89,90], creating a pro-inflammatory microenvironment that promotes bone resorption, angiogenesis, and carcinogenesis [48,68].

In addition, another pro-inflammatory molecule found enriched in OSCC samples is lipopolysaccharide (LPS) [91]. Accordingly, bacteria from the *Fusobacteria*, *Proteobacteria*, and *Bacteroidetes* phyla are Gram-negative and, therefore, have LPS in their cell wall. The results of the present study showed that the abovementioned Gram-negative phyla were the ones that increased in the OSCC group, while the Gram-positive phyla *Firmicutes* and *Actinobacteria* decreased in most of the studies. As a result, based on this evidence, LPS may play an essential role in oral carcinogenesis. Indeed, in both innate and adaptive immunity, LPS is recognized by LPS binding protein and Toll-like receptor (TLR)-4, stimulating cytokine transcription and eliciting LPS-induced inflammation. In T cells, the TLR4-ligand LPS activates TLR, steering cells toward type 1 polarization and prompting the expression of suppressor of cytokine signaling (SOCS) 1, thereby suppressing IL-10 expression. IL-10 is recognized as pivotal in shifting from inflammation that fosters tumors to antitumor immunity, and insufficient IL-10 signaling has been linked to the spontaneous development of tumors at a heightened frequency. LPS demonstrates the ability to activate TLR4 signaling in tumor cells, aiding these cells in evading attacks from cytotoxic lymphocytes and natural killer cells. This suggests a complex involvement of LPS in cancer development, particularly in mediating immune responses and influencing the tumor microenvironment [91].

Carcinogenic metabolites of oral bacteria, such as free radicals, support the chronic inflammatory process. Several species of the genus *Streptococcus* and *Neisseria* possess the enzyme alcohol dehydrogenase, which converts alcohol to acetaldehyde, a known carcinogen [50,71]. *Streptococci* also produce hydrogen peroxide, nitric oxide, and lactic acid, which create a hypoxic microenvironment by lowering tissue pH, thus increasing the risk of distant metastasis [48,92]. *F. nucleatum*, *Porphyromonas gingivalis*, and *Prevotella intermedia* produce sulfuric acids that are implicated in promoting and accumulating cellular DNA mutations [48,93]. *Prevotella intermedia* synthesizes methyl mercaptan, a carcinogenic metabolite involved in the development of angiogenesis and metastasis [94].

*F. nucleatum* and *Porphyromonas gingivalis* possess direct anti-apoptotic activity, stimulating the production of anti-apoptotic factors such as Bcl-2 and reducing the expression of the oncosuppressor gene p53 [88,95]. *F. nucleatum* is also a risk factor for the development of distant metastases due to the activation of p38 and subsequent expression of metalloproteases 9 and 13 [96].

### 4.2. Viral Content of Tissue and Saliva Samples in Adult Subjects with OSCC

The viral content of adult subjects with OSCC has been the most investigated both in terms of the number of cases and systematic reviews included in the present study, with particular attention to HPV and EBV. Indeed, a total of twenty-two studies assessed the viral content of neoplastic tissue or saliva samples in 25.551 adult OSCC subjects.

The most commonly used sampling methods and microorganism identification techniques were biopsy and PCR for the OSCC tissue samples and the salivary test and PCR for the saliva, respectively. It is important to consider that in many cases, HPV DNA was found using PCR. However, the finding of the viral genome does not imply that HPV is transcriptionally active and thus capable of activating its carcinogenic patterns [97].

#### 4.2.1. HPV in Tissue and Saliva Samples of Adult Subjects with OSCC

Eleven studies included in the present umbrella review reported increased HPV content in tissue samples from the OSCC group, three other studies reported increased HPV content in the OSCC group compared with the healthy control group, and one study reported a lower HPV content in the OSCC group than in other squamous cell carcinomas. The salivary HPV content in the OSCC subjects was increased in three systematic reviews (in one case compared with samples from OPMD subjects), whereas it was decreased in one study compared with saliva samples from patients with other (non-oral) SCCs. Eighteen studies recorded an increased incidence of HPV in the OSCC tissue samples and saliva of adult OSCC patients, while one study recorded a decrease in HPV.

HPV is the causative agent of benign and malignant skin lesions as well as genital and oral mucosa [98]. Specifically, the oncogenic role of HPV in squamous cell carcinoma of the cervix and oropharynx (OPSCC) has been established, but it remains controversial for oral carcinogenesis [38,98,99,100,101].

The carcinogenic mechanisms of HPV include genomic instability, impairment of DNA repair mechanisms, cellular immortalization, and anti-apoptotic activity [100]. Notably, the viral protein E7 binds to p53, which is cleaved by E6, resulting in a loss of cell cycle control [100,102]. E7 also degrades retinoblastoma protein (pRb) and inhibits p27 and p21 by affecting cyclin-dependent kinase activity [103]. E7 mediates the overexpression of p16, which has been implicated in the mechanisms of oral carcinogenicity [99]. E6 inhibits p73 activity [27]. Both E6 and E7 induce DNA methylation, influencing epigenetic mechanisms [104]. Thus, both HPV proteins E6 and E7 are able to promote genetic mutations in the cells of the basal layer, which are then inherited by daughter cells [99].

HPV seems to be implicated in approximately 3–4% of OSCCs [99,100]. However, HPV infection rates are higher in OSCCs (6% to 58% worldwide) [41], particularly for high-risk HPV (HR-HPV) genotypes 16 and 18 (24% to 56% of HPV genotypes found in OSCCs worldwide [105]. Consistent with this, the data from the present study show that 55.08% of OSCC tissue samples in which the presence of HPV was investigated were positive and that HR-HPV 16 and 18 genotypes were the most common (38.47% and 21.45%, respectively). Other HR-HPV genotypes (HPV-13, -31, -33, -35, -56, 58, -68) were found in a low percentage of cases, probably because some systematic reviews only investigated the presence of HPV-16 and/or -18. Low-risk HPV genotypes (LR-HPV) -6 and -11 ranked second in the frequency of infections in the OSCC tissue samples, accounting for 7.80% and 4.78% of cases, respectively.

Among coinfections, HPV-16 and -18 and HPV-6 and -11 stood out in frequency. Several previous studies have reported that HPV coinfections play a synergistic role in squamous cell lesions (SIL) of the female genital mucosa [106,107,108]. A recent meta-analysis showed that high-grade SILs (HSILs) were significantly associated with HPV coinfections compared with single infections and that low-grade SILs (LSILs) were more common in coinfections. However, the association was not statistically significant [106]. In addition, multiple HPV infection was associated with longer duration than single infection (mean 68 months and 27 months, respectively) [106]. Thus, a longer duration of infection may be partially responsible for developing high-grade intraepithelial lesions, including carcinoma. Regarding oral HPV infection, to the best of our current knowledge, there are no studies investigating the duration of infection in the presence of multiple HPV or the possible increased risk of developing OSCC.

The three currently available HPV vaccines protect against infection, with the two HR-HPVs most commonly found in the OSCC tissue samples in the present study (HPV-16 and -18). Two of the three vaccines protect against the four most commonly found viral genotypes (HPV-16, -18, -6, and -11). Indeed, Cervarix (Bivalent, GSK, Brentford, United Kingdom) protects against infection with HPV-16 and -18; Gardasil (Quadrivalent, Merck & Co, Kenilworth, New Jersey, United States of America) protects against HPV-6, -11, -16, -18; Gardasil9 (Nonavalent, Merck & Co, Kenilworth, New Jersey, United States of America) protects against HPV-6, -11, -16, -18, -31, -33, -45, -52, and -58 [24]. Although the Food and Drug Administration has not officially approved HPV vaccination for the prevention of oropharyngeal cancer, and knowledge of its effectiveness in reducing the incidence of OSCC is minimal, the risk of oral HPV-16 and -18 infection decreases significantly in the ten years following vaccination [109]. More data are available on reducing cervical cancer incidence in vaccinated women aged 16–19 years, for whom a 68–86% reduction in incidence has been reported [110]. In light of this, oral healthcare providers, who often intercept benign and malignant HPV-related oral lesions [24], play a key role in encouraging children aged 9–12 years, who benefit from the highest vaccine efficacy [111,112], and their caregivers to receive HPV vaccination [112].

Data from the Centers for Disease Control and Prevention show that women in the United States are more likely to develop HPV-related cancers yearly. The trend shows that women are at higher risk for cancers (cervical, vulvar, vaginal, and anal). At the same time, men appear to be most at risk for penile cancers and also for “posterior pharyngeal” cancers, for which the male-to-female ratio is 5.44:1 (data accessed 29 August 2023, and freely available online at https://www.cdc.gov/hpv/parents/cancer.html). The results of the present study also recorded an increased risk of HPV-related OSCC in men, with the sex ratio decreasing to 2.6:1.

Only one study reported a decrease in HPV in OSCC tissue samples in the OSCC group compared with other SCCs, such as OPSCC and squamous cell carcinoma of the larynx, for which HPV was identified as a carcinogenic risk factor [38]. In 2021, 33% of OPSCCs were HPV-positive worldwide, with a higher incidence in high-income countries such as Italy (46.1%), the United Kingdom (52%), and the United States of America (71%) [113]. The present study showed a higher incidence of HPV in Asian countries such as India, China, Japan, and Taiwan, followed by the United States of America. A low incidence rate was recorded in European countries, especially in the Nordic countries and in the African continent, probably due to the lower availability of primary care and epidemiological data.

The results of the present study revealed that OSCCs in which viral content was examined were most common on the palatine tonsils (n = 1820) and tongue (n = 930). The higher incidence at these two sites may be related to the geographic proximity of the palatine tonsils and tongue to the oropharynx [54]. Some tongue OSCC may originate from the base of the tongue, which is classified as part of the oropharynx, while the mobile tongue is considered part of the oral cavity. Indeed, Orrù et al. [114] reported that most HPV-related OSCCs were localized at the base of the tongue and on the lingual or palatine tonsils; in contrast, HPV-related benign lesions mainly were found at oral sites exposed to microtrauma (e.g., labial mucosa, commissures, hard palate, and vermilion). Considering that the American Joint Committee on Cancer has identified HPV-positive OPSCC as distinct entities from HPV-negative OPSCC, while this subdivision does not exist for OSCC [38], it is essential to distinguish between OSCC and OPSCC and avoid misclassification accurately. Indeed, HPV-positive OPSCCs require different treatments [115], are more common in young nonsmokers or alcohol users, and are associated with better outcomes, locoregional control, and higher survival [116,117]. Despite the paucity of evidence, a recent study showed that HPV-positive OSCC patients had worse overall survival and distant control than HPV-negative OSCC patients [38]. These results may suggest a different prognostic role of HPV in OSCC compared with OPSCC, although the mechanisms underlying these differences are not yet known, and other previous studies are inconsistent with these recent findings [118,119].

#### 4.2.2. EBV in Tissue and Saliva Samples of Adult Subjects with OSCC

Four studies included in the present umbrella review reported an increased content of EBV in neoplastic tissue samples and two in the saliva samples from OSCC subjects compared with healthy ones and patients with OPMD (one study). Overall, seven studies registered an increased incidence of EBV in the neoplastic tissue samples and saliva of adult OSCC patients, and none showed a decrease.

Although approximately 90% of the human population is infected with EBV, which is transmitted via saliva, and the virus is commonly detected in human saliva [105], an increase in the saliva of adult subjects with OSCC was presently highlighted. EBV was found in 25.9–82.5% of OSCC tissue samples. Moreover, regarding EBV in carcinogenic processes, the IARC declared EBV as the causative agent of nasopharyngeal carcinoma in 1997, being also involved in the genesis of Hodgkin’s lymphoma [120], natural killer/lymphocyte T-cell lymphoma [121], lymphoproliferative disorders [122], and gastric cancer [123]. However, the role of EBV in oral carcinogenesis remains to be elucidated since there is still no clear evidence of a reproducible etiopathogenetic link between EBV infection and the development of OSCC [41,101].

The controversial results of the studies currently published in the literature may be partly related to the sensitivity and specificity of the different viral identification methods (e.g., PCR, Nested PCR, IHC, and ISH) and viral targets (e.g., viral DNA, RNA, and proteins) [56]. In addition, due to the weakness of the EBV transformation gene, multiple methods must be used in a single experiment [56,124].

The study by She et al. [56], which was included in this review, showed that EBV DNA, mRNA, and proteins were expressed in most cells of the OSCC tissue samples, and the EBV DNA regions of BamHIW and EBNA2 were found, demonstrating a different capacity in detecting the EBV genome. The EBV genome could be derived from the oropharynx and found in the saliva of individuals with cancer [85]. Also, in the study by Shimakage et al. [125], BamHIW, a probable oncogene found in 275 cases, was considered a promising biomarker for detecting EBV. In contrast, in the study by Kikuchi et al. [126], EBNA1 (50.2%) was found to be more strongly expressed than LMP-1 (10.7%) in the same OSCC tissue samples.

LMP-1 was found in 777 cases in the present study and is another oncogenic EBV protein likely associated with OSCC [105]. LMP-1 may contribute to epithelial cell transformation by activating transcription factors [127].

In addition, intra-kingdom microbial interactions established by EBV must be considered. In particular, several studies [128,129,130,131] confirmed an increased representation of *Porphyromonas gingivalis* in EBV-positive individuals with chronic periodontitis or aggressive periodontitis. Kato et al. [128] found the coexistence of *Porphyromonas gingivalis* and EBV in 80% of probing pocket depths greater than 5 mm in a Japanese sample with chronic periodontitis. These results suggest a microbiological interaction between two microorganisms capable of activating carcinogenic pathways.

A systematic review presently considered [41] reported an increased prevalence of HPV-EBV coinfections in the OSCC tissue samples. Several authors have considered coinfections, especially of HR-HPV and EBV, a risk factor for the initiation and development of carcinoma [56,132,133,134,135]. The higher prevalence of HPV-EBV coinfections in the OSCC group is not evidence of viral cooperation. The few studies available have shown that co-infected epithelial cells have a higher risk of transformation and invasion than proliferation [134]. Al Moustafa et al. [132] hypothesized that HPV oncoproteins (E6 and E7) co-occur with those of EBV (LMP-1, LMP-2, EBNA-1, BARF -1) during cell transformation. Fathallah et al. [98] demonstrated that LMP-1 can potentially inhibit Toll-like receptor 9, creating a favorable environment for secondary infections.

#### 4.2.3. HSV in Tissue and Saliva Samples of Adult Subjects with OSCC

HSV-1 is another virus responsible for benign oral manifestations in children and adults, but it has not been associated with the development of malignant oral lesions, such as OSCC [101]. Only one systematic review included in the present study investigated the presence of HSV-1 in OSCC-tissue samples and found no significant variation among subjects with OSCC.

#### 4.2.4. HCV in Tissue and Saliva Samples of Adult Subjects with OSCC

Although none of the systematic reviews included in the present study investigated HCV content in neoplastic tissue or saliva samples from adults with OSCC, it should be noted that the association between HCV and OSCC was first described by Nagao et al. in 1955 [136]. In recent years, several studies have been conducted to investigate the possible role of HCV in the development of OPMD and OSCC [137,138,139,140].

Campisi et al. [137] found an association between HCV infection and the occurrence of oral lichen planus (OLP) based on a statistically significant geographic association with HCV infection endemic areas such as Mediterranean countries, the United States and Japan, as also shown by the study of Lodi et al. [140]. It has been hypothesized that HCV is capable of eliciting a local immune-mediated response to HCV epitopes that promotes the development of OLP [140].

In the retrospective study by Gandolfo et al. involving 402 individuals with OLP [138], 44% of those who later developed OSCC were infected with HCV. Yoshida et al. [139] reported the presence of HCV infection in 16.7–24% of Japanese patients with OSCC. Takata et al. [141] also registered an increased incidence of HCV antibodies in Japanese patients with OSCC. However, the authors hypothesized that the association might be due to the older age of patients with OSCC, reflected in an increased risk of HCV infection. Therefore, the association would be causal and not etiologically significant.

In synthesis, recent studies in the literature show discrepancies between the possible association between HCV infection, the presence of HCV in OSCC biopsy specimens, and its oncogenic role in oral carcinogenesis [23,142].

### 4.3. Fungal Content of OSCC-Tissue Samples or Saliva in Adult Subjects with OSCC

The three studies examining fungal content in neoplastic tissue and saliva samples from adult OSCC patients had 667 cases. The fungal kingdom was the least studied regarding the number of systematic reviews, sample size of OSCC cases, and diversity of fungal genera reported.

The most commonly used sampling methods and techniques to identify microorganisms were biopsy and culture for OSCC tissue samples and saliva test and culture for saliva.

Previous studies [143] have shown a reduction in individual fungal species in patients with OSCC and head and neck cancer, such as yeast of the genus *Malessezia* and *Schizophyllum*, which has anticancer activity due to the production of the polysaccharide schizophyllan. Conversely, other fungal genera, such as *Candida*, *Giberella*, *Aspergillus*, and *Hannaella*, were found to be increased in OSCC tissue samples [143].

#### 4.3.1. Candida Genus in Tissue and Saliva Samples of Adult Subjects with OSCC

*Candida* was the most investigated genus of fungus in the systematic reviews included in the present study. In part, the reason for this may be related to the greater representation of *Candida* among fungi in the oral microbiome and, in part, to the fact that *Candida* is the most easily isolated and most studied genus of fungus [48].

Five studies reported an increase in the genus *Candida* in saliva and OSCC tissue samples in adult subjects with OSCC compared to healthy controls and OPMD. In no case was a decrease in *Candida* reported in the OSCC group.

One systematic review in the present study reported increased *Candida* in OSCC tissue samples from adults who underwent chemotherapy or radiotherapy. As reported in many previous studies [144,145,146], oral and oropharyngeal candidiasis is a complication after radiotherapy and chemotherapy, especially in head and neck cancers. Radiation or chemotherapy also increases oral fungal species’ pathogenicity and antifungal drug resistance, especially *Candida* [144].

Previous studies [27,55,147,148] have shown that elevated *Candida* levels in the oral cavity are associated with an increased risk of OPMD or OSCC.

In the present umbrella review, no study specified the Candida species. In contrast, several previous studies documented that the most common *Candida* species found in OSCC groups was *Candida albicans*, with percentages ranging from 68% to 86% [28,35,149,150,151]. Instead, non-*Candida albicans* subspecies, such as *Candida tropicalis, C. parapsilosis,* and *C. glabrata,* were classified as pathogenic fungi, and their dominance was considered a sign of oral dysbiosis [35].

*Candida albicans* is considered the most virulent *Candida* species and can trigger carcinogenesis by producing carcinogens and creating an inflammatory environment [19,47]. Several mechanisms have been proposed for the role of *Candida albicans* in OPMD and OSCC, including the production of nitrosamines, acid aspartyl proteinase, acetaldehyde, and candidalysin (a cytolytic toxin); the overexpression of Ki-67, p53, prostaglandin-endoperoxide synthase 2 (COX -2), and proinflammatory cytokines; the reduction of β-defensins [27].

Nitrosamines are carcinogens that, alone or in combination with other chemical compounds, can cause activation of protooncogenes and, consequently, dysplastic lesions [19]. The hyphal structure of *Candida albicans* could deliver the nitrosamine products from saliva to keratinocytes, triggering OSCC [8].

*Candida albicans* is capable of expressing alcohol dehydrogenase 1, the enzyme that converts ethanol to acetaldehyde, a known carcinogen with mutagenic activity [8,19,55], resulting in chromosomal aberrations, point mutations and interference with DNA repair enzymes and antioxidant glutathione, leading to an increase in oxygen radicals [19,35]. Among the fungi of the *Candida* genus, *Candida tropicalis*, *C. parapsilosis*, and *C. glabrata* can also express the enzyme alcohol dehydrogenase 1, although to a lesser extent than *Candida albicans* [19,27]. The conversion of ethanol to acetaldehyde by oral fungi is also influenced by alcohol consumption and smoking habits [8]. In fact, the International Agency for Research on Cancer has classified acetaldehyde associated with alcohol consumption as a Group I human carcinogen [8,17]. However, none of the systematic reviews included in the present study that examined the fungal composition of saliva or OSCC tissue samples in adult subjects mentioned the risk factors for OSCC in the study group.

In addition to the direct effects of *Candida albicans*, it is crucial to consider the cross-congregational interactions that the fungus maintains with various bacteria, such as *Porphyryromonas gingivalis*. *Candida albicans* can protect *Porphyromonas gingivalis* from the host immune system, thus supporting bacterial infection and the above-mentioned carcinogenic mechanisms of *Porphyromonas gingivalis* [19].

None of the studies included in the present umbrella review specified the *Candida* species found and, thus, the genotype of *Candida albicans*. From studies [27,35,152], genotype C of *Candida albicans* is more prevalent in OPMD, especially in the so-called Candida leukoplakia (CL), while genotype A is more common in individuals with OSCC, although no significant difference in the ability to produce carcinogens was found between the two genotypes. Nakazawa et al. [153] showed the presence of Candida in 62.5% of cases of oral leukoplakia and a higher rate of DNA alterations in CLs than in leukoplakia not superinfected with Candida. The study by Chiu et al. [154] recorded a higher percentage of Candida infections in subjects with multiple oral leukoplakias (47.9%) than in subjects with a single oral leukoplakia (19.0%). The studies by Bensal et al. [28] and Ayuningtyas et al. [35] also reported a higher risk of malignant transformation from CL than from non-Candida leukoplakias and other OPMD, respectively. The results of the present study showed a threefold increase in *Candida* in saliva or OSCC tissue samples from adult subjects with OSCC compared with subjects with OPMD. However, none of the included systematic reviews specified the type of OPMD. However, in no case was a lower amount of *Candida* found in the OSCC group than in the OPMD group, suggesting, in agreement with previous studies [27,28,35,153,154,155], that increased numbers of fungi in the oral microbiome are associated with OPMD, OSCC, and a significant risk of malignant transformation from OPMD to OSCC.

It should be noted that in the present study, no systematic review indicated whether the OSCC group in which *Candida* was found had oral candidiasis. *Candida albicans* is also commonly found in healthy individuals [26], so it would be important to determine in further studies whether the *Candida* found in saliva or OSCC tissue samples is already pathogenic or occurs as an opportunistic commensal.

#### 4.3.2. Aspergillus, Acremonium, and Morchella in Tissue and Saliva Samples of Adult Subjects with OSCC

A systematic review included in the present study recorded an increase in *Aspergillus* and *Acremonium* and a decrease in *Morchella* fungi in the OSCC group compared to the OPMD group.

Lin et al. [156] reported that *Aspergillus*, and in particular the species *Aspergillus rambellii*, is a significant promoter of colorectal cancer. In contrast, in the study by Ghfar et al. [157], the metabolites terretonin N and butyrolactone I produced by *Aspergillus terreus* were found to have anticancer activity against human prostate and ovarian adenocarcinoma cells.

Liu et al. [158] showed the significant antioxidant activity of polysaccharide FMP-1 produced by *Morchella esculenta*, indicating a probiotic and anticancer activity.

Although the study by Heng et al. [159] reported a significant increase in *Acremonium* and *Aspergillus* during the progression of OSCC, to our current knowledge, there are no studies demonstrating the pro or anti-carcinogenic activity of *Aspergillus*, *Morchella,* or *Acremonium* in relation to OSCC.

### 4.4. Limitations and Strengths

The heterogeneity of the extracted data and the numerous missing data are the main limitations of the present study, which precluded the possibility of meta-analysis. The variations among the extracted data relate to the different techniques and targets used to identify microorganisms, underscoring the need to validate a standardized protocol for analyzing the oral microbiome.

Another limitation of the present review is the repetition of some studies included in several systematic reviews that examined bacteria in both saliva and OSCC tissue samples. Although the most frequently replicated studies were small in number, this may represent a further bias in the analysis and interpretation of results. Overlapping data sets were not found for studies on viruses or fungi.

However, to the best of our knowledge, this umbrella review is the first to provide a comprehensive picture of the oral microbiome (bacteria, viruses, and fungi) in tissue and saliva samples from adult patients with OSCC, highlighting the reported variations, especially when compared to the healthy and OPMD groups.

Because some oral microorganisms have carcinogenic properties and an increase or decrease in these microorganisms may be involved in the processes of initiation, development, and metastasis of oral epithelial cells, the reversal of oral dysbiosis may be among the oral health measures to prevent OSCC onset and progression [160,161]. Further studies should investigate the possible carcinogenic mechanisms of the major oral microorganisms found in neoplastic tissue and saliva samples from adult patients with OSCC, reducing the confounding variables that are the main problem of the studies currently available in the literature. In addition, future studies would help provide a more comprehensive picture of the fungal composition in subjects with OSCC by examining variations in oral fungi outside the genus *Candida*.

## 5. Conclusions

The present umbrella review has shown that oral dysbiosis can occur in adults (>18 years) with OSCC.

Regarding the bacterial kingdom, the phyla of *Fusobacteria*, *Proteobacteria*, and *Bacteroidetes* were frequently increased compared to the healthy and OPMD subjects. A greater heterogeneity and abundance of genera and species was observed in the saliva than in OSCC tissue samples, especially for the phylum *Bacteroidetes*. The most abundant bacteria found were Gram-negative and periodontal pathogens, while phyla *Firmicutes* and *Actinobacteria* decreased, especially for the genera *Streptococcus* and *Rothia*.

HPV and EBV showed a significant increase in the oral cavity of individuals with OSCC. HPV was the most commonly studied virus and was found to increase primarily in the OSCC tissue rather than saliva samples. HPV-16 and -18 were the most frequently found genotypes (38.47% and 21.45%, respectively), followed by other HR-HPV, multiple HPV co-infections, and LR-HPV.

The fungal kingdom was the least studied in the 32 systematic reviews included in this study. Moreover, the studies examining variations in oral fungi focused mainly on the genus Candida, which was more prevalent in OSCC than non-OSCC patients in all studies.

Future perspectives in clinical exploration regarding the role of oral microorganisms in the development and progression of oral cancer entail several crucial points.

Firstly, understanding the specific mechanisms and interactions between oral microorganisms and cancer progression could pave the way for targeted preventive strategies. In this context, further investigations hold the promise of offering valuable insights into the development of vaccines or antimicrobial therapies aimed at preventing OSCC.

Secondly, the substantial association identified between bacteria and OSCC might hold clinical significance in the realm of cancer screening. Exploring the potential of using oral microorganisms as biomarkers for cancer could revolutionize early detection and screening protocols, offering a non-invasive and potentially efficient method for identifying individuals at risk.

Lastly, considering that certain oral microorganisms possess carcinogenic properties and alterations in their populations may play roles in the initiation, development, and metastasis of oral epithelial cells, restoring the balance of oral microbiota—reversing oral dysbiosis—could emerge as a critical component of oral health measures aimed at preventing the onset and progression of OSCC. This emphasizes the potential for interventions focusing on restoring a healthy oral microbial environment to mitigate the risk and impact of oral cancer development and spread.

## Figures and Tables

**Table 1:** Data primarily concerning bacteria extracted and collected from the included studies that had carried out a histopathologic analysis of OSCC-tissue lesions. Studies: first author, year and journal of publication, reference, number and design of included studies, meta-analysis, assessed quality, funding (if any). Population characteristics: sample size, mean age, gender ratio, country of origin of the sample, risk factor and history of OPMD, history of malignancies, other comorbidities, and ongoing treatments. OSCC characteristics: macroscopic features, location, staging and grading, microscopic features, first diagnosis (primary site/metastatic lesion), time to onset, chemotherapy (yes/no), radiotherapy (yes/no). Intervention: number of sample(s), method(s) of sample collection, microorganisms identification technique, target. Outcome(s): type(s) of phylum, genus and species of bacterium detected, number or percentage of OSCC-positive cases.

Studies	Population		Intervention	Outcome(s)
	Characteristics	OSCC		Bacteria
Bronzato J.D., 2020 Arch Oral Biol Studies: n.13 CCS n.13 Meta-analysis Moderate quality This study was supported by CAPES, FAPESP, and CNPq.	Sample size: n.294 of case Mean age: MD Gender ratio: MD Country: USA n.52 China n.40 India n.40 Germany n.33 Hungary n.31 Wales n.30 Sri Lanka n.27 Japan n.21 Yemen n.N/D Saudi Arabia n.N/D Risk factors for OSCC: MD History of OPMD: MD Time to OPMD onset: MD Previous history of malignancies: MD Other comorbidities: MD Other ongoing treatments: MD	Macroscopic features: MD Location: N/D oral cavity n.161; gingiva n. MD; tongue n. MD; cheek n. MD; oral floor n. MD; mandible n. MD; buccal mucosa n. MD. Staging: MD Grading: MD Microscopic features: MD First diagnosis: MD Time to onset: MD Chemotherapy: MD Radiotherapy: MD	Sample(s): n.294 Method(s) of sample collection: Biopsy n.125 Swab n.146 Biopsy and Swab n.20 Sterile paper point n.3 Microorganisms identification technique: Culture n.142 PCR n.152 Target: 16S rDNA n.42 16S rRNA n.107 MD n.145	Than the healthy control group Fusobacteria: ↑ Fusobacterium: n.132 of OSCC case Type(s) of Fusobacterium species detected: F. nucleatum; F. naviforme; F. periodonticum; F. canifelinum; F. oral taxon (A71, 203, 370); F. necrophorum; F. gonidiaformans; F. simiae. Type(s) of F. nucleatum subspecies: F. nucleatum ssp. nucleatum; F. nucleatum ssp. vicentii; F. nucleatum ssp. polymorphum; F. nucleatum ssp. animalis.
Gopinath, 2019 Crit Rev Oncol Hematol Studies: n.7 CCS n.7 No meta-analysis Low quality No funding	Sample size: n.199 of case/n.201 of the healthy control group Mean age: 60.85 y.o.; range MD Gender ratio: MD Country: United Kingdom n.10 USA n.64 Yemen n.20 Sri Lanka n.25 China n.80 Risk factors for OSCC: N/D History of OPMD: MD Time to OPMD onset: MD Previous history of malignancies: MD Other comorbidities: MD Other ongoing treatments: MD	Macroscopic features: MD Location: tongue n.39 MD n.160 Staging: MD Grading: MD Microscopic features: MD First diagnosis: MD Time to onset: MD Chemotherapy: MD Radiotherapy: MD	Sample(s): n.199 Method(s) of sample collection: Biopsy n.104 Swab n.95 Microorganisms identification technique: DNA isolation kit n.30 Incubation in Proteinase K and DNA purification kit n.10 Incubation in Proteinase K and DNA easy kit n.15 Gentra Puregene Tissue kit n.25 QIAamp DNA Stool mini kit n.39 QIAamp DNA Mini kit n.80 Target: 16s rRNA: n.10 V1-V3 region: n.45 V4-V5 region: n.90 V4 region: n.54	Than the healthy control group ↑ Fusobacteria: ↑ Fusobacterium Type(s) of Fusobacterium species detected: F. nucleatum ssp. polymorphum F. naviforme ↑ Spirochaetes ↑ Proteobacteria: ↑ Campylobacter Type(s) of Campylobacter species detected: C. Oral taxon 44 ↑ Pseudomonas Type(s) of Pseudomonas species detected: ↑ Pseudomonas aeruginosa ↑ Ralstonia Type(s) of Ralstonia species detected: Ralstonia insidosa ↑ Bacteroidetes: ↑ Capnocytophaga ↓ Actinobacteria: ↑ Corynebacterium ↑ Atopobium ↑ Actinomyces ↑ Rothia ↑ Micrococcus ↑ Clavibacter michiganensis Type(s) of Clavibacter michiganensis species detected: Clavibacter michiganensis tellarius ↓/↑ Firmicutes: ↑ Enterococcus ↑ Gemella Type(s) of Gemella species detected: Gemella haemolysans Gemella morbillorum ↑ Streptococcus Type(s) of Streptococcus species detected: S. salivarius S. oral taxon 058 S. gordonii S. parasanguinis ↑ Johnsonella Type(s) of Johnsonella species detected: Johnsonella ignava ↑ Peptostreptococcus: Type(s) of Peptostreptococcus species detected: Pepetosptreptoccus stomatis
Gupta, 2020 Clin Oral Investig Studies: n.7 CSS n.7 No meta-analysis Low quality No funding	Sample size: n.513 of case/n.354 of the healthy control group Mean age: MD Gender ratio: 39M/44F/430MD Country: Japan n.58 Sri Lanka n.53 India n.60 Iran n.83 Germany n.191 China n.68 Risk factors for OSCC: Betel chewers n.44 History of OPMD: MD Time to OPMD onset: MD Previous history of malignancies: MD Other comorbidities: MD Other ongoing treatments: MD	Macroscopic features: MD Location: MD Staging: MD Grading: MD Microscopic features: MD First diagnosis: MD Time to onset: MD Chemotherapy: MD Radiotherapy: MD	Sample(s): n.513 Method(s) of sample collection: Swab n.58 Biopsy n.455 Microorganisms identification technique: PCR: n.128 RT-PCR: n.58 Culture: n.131 Giemsa: n.68 IHC: n.274 ELISA: n.121 Target: MD	↑ Than the healthy control group (prevalence 31.92%) Proteobacteria: Helicobacter Type(s) of Helicobacter species detected: ↑ Helicobacter pylori: n.165 (prevalence 32.16%) of OSCC case
Huybrechts, 2020 Cancer Epidemiol Biomarkers Prev Studies: n.13 CS n.2 CCS n.11 No meta-analysis Low quality This study was supported by the Intramural Research Program of the National Cancer Institute at the National Institutes of Health and by the Research Foundation-Flanders 12h1519N.	Sample size: n.724 of case/n.1188 of the healthy control group Mean age: MD Gender ratio: MD Country: MD Risk factors for OSCC: MD History of OPMD: MD Time to OPMD onset: MD Previous history of malignancies: MD Other comorbidities: MD Other ongoing treatments: MD	Macroscopic features: MD Location: MD Staging: MD Grading: MD Microscopic features: MD First diagnosis: MD Time to onset: MD Chemotherapy: MD Radiotherapy: MD	Sample(s): n.724 Method(s) of sample collection: Biopsy n.724 Microorganisms identification technique: N/D Target: MD	↑ Bacteroidetes: ↑ Capnocytophaga ↑ Fusobacteria: ↑ Fusobacterium Firmicutes: ↑ Dialister ↑ Peptostreptococcus ↑ Parvimonas
Mallika, 2020 Trans Cancer Res Studies: n.8 CCS n.8 No meta-analysis Moderate quality No funding	Sample size: MD Mean age: MD Gender ratio: MD Country: MD Risk factors for OSCC: MD History of OPMD: MD Time to OPMD onset: MD Previous history of malignancies: MD Other comorbidities: MD Other ongoing treatments: MD	Macroscopic features: MD Location: MD Staging: MD Grading: MD Microscopic features: MD First diagnosis: MD Time to onset: MD Chemotherapy: yes (one study) Radiotherapy: yes (two studies)	Sample(s): MD Method(s) of sample collection: Biopsy n.MD Swab n.MD Brush n.MD Microorganisms identification technique: Culture n.MD IHC n.MD ISH n.MD PCR n.MD Target: MD	Proteobacteria: Helicobacter Type(s) of Helicobacter species detected: ↑ Helicobacter pylori
Muthusamy, 2023 Cureus Studies: n.6 CCS n.6 Meta-analysis Critically low quality This study was supported by the Indian Council of Medical Research (ICMR) under the Nurturing Clinical Scientist (NCS) scheme HRD/Head-NCS-2019-02.	Sample size: n.373 of case/n.326 of healthy control group/n.73 of OPMD group Mean age: MD Gender ratio: MD Country: MD Risk factors for OSCC: MD History of OPMD: MD Time to OPMD onset: MD Previous history of malignancies: MD Other comorbidities: MD Other ongoing treatments: MD	Macroscopic features: MD Location: MD Staging: MD Grading: MD Microscopic features: MD First diagnosis: MD Time to onset: MD Chemotherapy: MD Radiotherapy: MD	Sample(s): n.373 Method(s) of sample collection: Blood analysis n.50 Biopsy n.211 Biofilm sampling n.21 Brush n.91 Microorganisms identification technique: PAP technique n.50 Culture n.21 Nested PCR n.120 ELISA n.132 PCR n.50 Target: Herpes Select-1 n.132 16s RNA n.50	Fusobacteria: ↑ Fusobacterium *p* = 0.05 Firmicutes: ↑ Streptococcus *p* = NSS Bacteroidetes: ↑ Prevotella *p* = NSS ↑ Porphyromonas *p* = NSS Proteobacteria: ↑ Neisseria: *p* = NSS
Ramos, 2020 Oral Maxillofac Surg Studies: n.4 CCS n.1 MD n.3 No meta-analysis Critically low quality This study was supported by the National Council for Scientific and Technological Development (CNPq) (Project:211309/ 2013-3) and the Foundation for Research Financial Support in the State of Rio de Janeiro (FAPERJ) (Project: E26/ 1033.001/2012).	Sample size: n.124 of case/n.20 of the healthy control group/n.27 of the control group with oral fibroepithelial polyp Mean age: MD Gender ratio: N/D Country: MD Risk factors for OSCC: Tobacco: n.N/D Alcohol: n.N/D History of OPMD: MD Time to OPMD onset: MD Previous history of malignancies: MD Other comorbidities: MD Other ongoing treatments: MD	Macroscopic features: MD Location: tongue n.39 MD n.85 Staging: MD Grading: MD Microscopic features: MD First diagnosis: MD Time to onset: MD Chemotherapy: MD Radiotherapy: MD	Sample(s): n.124 Method(s) of sample collection: Biopsy n.64 Swab n.40 Swab and Biopsy: n.20 Microorganisms identification technique: MiSeq n.85 Ion torrent n.39 Target: V4 region n.39 V1-V3 region n.45 V4-V5 region n.40	↑ Firmicutes: ↑ Dialister ↑ Catonella ↑ Peptostreptococcus ↑ Peptococcus ↑ Filifactor ↑ Parvimonas ↓ Bacteroidetes: ↑ Capnocytophaga ↑/↓ Fusobacteria: ↑ Fusobacterium Type(s) of Fusobacterium species detected: ↑ F. nucleatum ssp. polymorphum Proteobacteria: ↑ Campylobacter Pseudomonas Type(s) of Pseudomonas species detected: ↑ Pseudomonas aeruginosa Actinobacteria: ↑ Atopobium
Shen, 2023 Arch Oral Biol Studies: n.2 CCS n.2 No meta-analysis High quality This study was supported by the China-Japan Friendship Hospital Research Project Foundation [grant number 2020-1-QN-2].	Sample size: n.25 of case/n.15 of the healthy control group/n.17 of the OPMD control group Mean age: 56.25 y.o.; range MD Gender ratio: MD Country: USA n.16 Malaysia n.9 Risk factors for OSCC: MD History of OPMD: MD Time to OPMD onset: MD Previous history of malignancies: MD Other comorbidities: MD Other ongoing treatments: MD	Macroscopic features: MD Location: MD Staging: MD Grading: MD Microscopic features: MD First diagnosis: MD Time to onset: MD Chemotherapy: MD Radiotherapy: MD	Sample(s): n.25 Method(s) of sample collection: Swab n.25 Microorganisms identification technique: Incubation Proteinase K & DNA easy kit n.16 DNA extraction kit n.9 Target: 16s rRNA n.25 V4 region n.16 RFLP gene n.9	Than the healthy and the OPMD control group Fusobacteria: ↑ Fusobacterium ↑ Proteobacteria: ↑ Neisseria ↓ Firmicutes: ↑ Gemella ↑ Granulicatella Actinobacteria: ↓ Rothia Than the healthy control group ↑ Bacteroidetes ↓ Firmicutes: ↓ Streptococcus ↓ Veillonella ↑/↓ Actinobacteria Than the OPMD control group ↓ Bacteroidetes ↓ Fusobacteria
Su Mun, 2021 Int J Environ Res Public Health Studies: n.7 CCS n.7 No meta-analysis High quality This study was supported by the International Medical University of Malaysia.	Sample size: n.280 of case/n.191 of the healthy control group Mean age: MD Gender ratio: MD Country: Yemen n.20 Sri Lanka n.25 China n.135 USA n.100 Risk factors for OSCC: Betel nut chewers n.N/D Tobacco n.N/D Shammah (smokeless tobacco) n.N/D Alcohol n.N/D History of OPMD: MD Time to OPMD onset: MD Previous history of malignancies: MD Other comorbidities: MD Other ongoing treatments: MD	Macroscopic features: MD Location: MD Staging: MD Grading: MD Microscopic features: MD First diagnosis: MD Time to onset: MD Chemotherapy: MD Radiotherapy: MD	Sample(s): n.280 Method(s) of sample collection: Biopsy n.280 Microorganisms identification technique: DNA isolation kit n.20 Gentra Puregene Tissue kit n.25 QIAampFast DNA Stool Mini kit n.61 TIANamp Swab DNA kit n.50 MD n.124 Target: V1-V3 region n.45 V3-V4 region n.135 MD n.100	Than the healthy control group Fusobacteria: ↑ Fusobacterium Type(s) of Fusobacterium species detected: ↑ F. oral taxon 204 ↑ F. parvimonas ↑ F. nucleatum ↑ F. nucleatum ssp. polymorphum Leptotrichia: Type(s) of Leptotrichia species detected: ↓ Leptotrichia oral taxon 225 Actinobacteria: ↑ Mobiluncus ↑/↓ Actinomyces ↓ Rothia Type(s) of Rothia species detected: ↓ Rothia dentocariosa ↓ Rothia mucilaginosa ↑ Atopobium ↓ Propionibacterium Corynebacterium Type(s) of Corynebacterium species detected: ↓ Corynebacterium matruchotii ↓ Arthrobacter ↓ Microbacterium Defferibacteraceae: ↓ Mucispirillum Proteobacteria: ↑ Brevundimonas ↑ Aeromonas ↑ Frateuria ↑ Caulobacter ↑ Pseudomonas Type(s) of Pseudomonas species detected: ↑ Pseudomonas aeruginosa Aggregatibacter Type(s) of Aggregatibacter species detected: ↑ Aggregatibacter segnis ↑ Campylobacter Type(s) of Campylobacter species detected: ↑ C. concisius ↑ C. rectus ↑ Citrobacter Type(s) of Citrobacter species detected: ↑ Citrobacter koseri ↓ Lautropia Type(s) of Lautropia species detected: ↓ Lautropia mirabilis ↓ Sphingomonas Firmicutes: ↓/↑ Streptococcus: Type(s) of Streptococcus species detected: ↑ S. dysgalactiae ↓ S. parasanguinis ↓ S. mitis ↓ S. oralis ↓ S. sp oral taxon 423 ↓ S. sp oral taxon 070 ↓ S. sp oral taxon 431 ↓ S. agalactiae ↓ Staphylococcus Type(s) of Staphylococcus species detected: ↓ Staphylococcus epidermidis ↑ Peptostreptococcus Type(s) of Peptostreptococcus species detected: ↑ Peptostreptococcus stomatis Granulicatella Type(s) of Granulicatella species detected: ↓ Granulicatella adicens ↓ Granulicatella elegans ↓ Paenibacillus ↑ Parvimonas Gemella: Type(s) of Gemella species detected: ↑ Gemella morbillorum Bacteroidetes: ↑ Capnocytophaga Type(s) of Capnocytophaga species detected: ↑ Capnocytophaga leadbetteri ↑ Prevotella Type(s) of Prevotella species detected: ↑ Prevotella salivae ↑ Prevotella loeschii ↑ Prevotella intermedia Porphyromonas Type(s) of Porphyromonas species detected: ↑ Porphyromonas cationiae ↓ Porphyromonas gingivalis
Yu, 2023 Heliyon Studies: n.2 CCS n.2 Meta-analysis Moderate quality This study was supported by a grant from the Qingdao Medical Talents Training Program [VYQ2020Y02].	Sample size: n.30 of case/n.26 of the healthy control group Mean age: 52.3 y.o.; range MD Gender ratio: MD Country: MD Risk factors for OSCC: MD History of OPMD: MD Time to OPMD onset: MD Previous history of malignancies: MD Other comorbidities: MD Other ongoing treatments: MD	Macroscopic features: MD Location: MD Staging: MD Grading: MD Microscopic features: MD First diagnosis: MD Time to onset: MD Chemotherapy: MD Radiotherapy: MD	Sample(s): n.30 Method(s) of sample collection: Biopsy n.30 Microorganisms identification technique: N/D next-generation sequencing technology n.30 Target: V4 region n.10 V1-V3 region n.20	Than the healthy control group Fusobacteria ↑ Fusobacterium: *p* = 0.000 ↑ Proteobacteria: ↓ Haemophilus *p* = 0.000 ↓ Actinobacteria: ↓ Rothia ↓ Firmicutes: ↓ Streptococcus *p* = 0.032 ↓ Bacteroidetes: ↑ Prevotella: NSS

Abbreviations: number, “n.”; years old, “y.o.”; percentage, “%”; greater than, “>”; missing data, “MD”; not defined, “N/D”; not statistically significant, “NSS”; not significant, “NS”; *p*-value, “*p*”; male, “M”; female, “F”; case-control study, “CCS”; cohort study, “CS”; cross-sectional study, “CSS”; retrospective cross-sectional study, “RCSS”; prospective study, “PS”; non-randomized study, “NRS”; oral squamous cell carcinoma, “OSCC”; oral potentially malignant disorder, “OPMD”; United States of America, “USA”; polymerase chain reaction, “PCR”; real time polymerase chain reaction, “RT-PCR”; quantitative polymerase chain reaction, “qPCR”; immunohistochemistry, “IHC”; in situ hybridization, “ISH”; enzyme-linked immunosorbent assay, “ELISA”; immunofluorescence, “IF”; immunoperoxidase, “IP”; nucleic acid sequence based amplification, “NASBA”; Papanicolaou technique, “PAP technique”; Messenger Ribonucleic Acid, “mRNA”; Ribosomal Ribonucleic Acid, “rRNA”; RiboNucleic Acid, “RNA”; DeoxyriboNucleic Acid, “DNA”; Ribosomal DeoxyriboNucleic Acid, “rDNA”; subspecies, “ssp.”; Fusobacterium, “F.”; Campylobacter, “C.”; Streptococcus, “S.”; restriction fragment length polymorphism, “RFLP”; Human Papilloma Virus, “HPV”; Low Risk Human Papilloma Virus, “LR-HPV”; High Risk Human Papilloma Virus, “HR-HPV”; Herpes Simplex Virus, “HSV”; Epstein Barr Virus, “EBV”; Epstein Barr Nuclear Antigen, “EBNA”; Epstein Barr Encoding Region, “EBER”; Latent Membrane Protein, “LMP”; human telomerase reverse transcriptase, “hTERT”; B-cell lymphoma 2, “BCL-2”; Bahm HI N fragment rightward open reading frame, “BNRF”; Bahm HI A fragment rightward open reading frame, “BARF”; Bahm HI H fragment rightward open reading frame, “BHRF”; Bahm HI N fragment leftward open reading frame, “BZLF”; BahmHI M fragment leftward open reading frame, “BMLF”; BahmHI Z fragment leftward open reading frame, “BZLF”; increased, “↑”; decreased, “↓”.

**Table 2:** Data primarily concerning viruses extracted and collected from the included studies that had carried out a histopathologic analysis of OSCC-tissue lesions. Studies: first author, year and journal of publication, reference, number and design of included studies, meta-analysis, assessed quality, funding (if any). Population characteristics: sample size, mean age, gender ratio, country of origin of the sample, risk factor and history of OPMD, history of malignancies, other comorbidities, and ongoing treatments. OSCC characteristics: macroscopic features, location, staging and grading, microscopic features, first diagnosis (primary site/metastatic lesion), time to onset, chemotherapy (yes/no), radiotherapy (yes/no). Intervention: number of sample(s), method(s) of sample collection, microorganisms identification technique, target. Outcome(s): type(s) and genotype of virus detected, number or percentage of OSCC-positive cases.

Studies	Population		Intervention	Outcome(s)
	Characteristics	OSCC		Viruses
Chaitanya N.C., 2016 J Cancer Res Ther Studies: n. 11 CCS n.11 Meta-analysis Critically low quality No Funding	Sample size: n. N/D of case/n. 3212 of the healthy control group Mean age: MD Gender ratio: MD Country: MD Risk factors for OSCC: MD History of OPMD: MD Time to OPMD onset: MD Previous history of malignancies: MD Other comorbidities: MD Other ongoing treatments: MD	Macroscopic features: MD Location: tongue n. MD buccal mucosa n. MD dentoalveolar complex n. MD oral floor n. MD Staging: MD Grading: MD Microscopic features: MD First diagnosis: MD Time to onset: MD Chemotherapy: MD Radiotherapy: MD	Sample(s): n. N/D Method(s) of sample collection: Serum analysis n. MD Biopsy n. MD Brush n. MD Microorganisms identification technique: N/D Target: HPV DNA n.MD	Than the healthy control group ↑ HPV: n.N/D of OSCC case 20.34% OSCC of the tongue 8.70% OSCC of the oral floor 8.00% OSCC of the dentoalveolar complex 5.00% OSCC of the buccal mucosa Genotype(s) of HPV detected: MD
Christianto S., 2022 Laryngoscope Studies: n. 22 CS n.22 Meta-analysis Critically low quality No funding	Sample size: n.3065 of case Mean age: MD Gender ratio: 2155M/720F/190 N/D Country: MD Risk factors for OSCC: MD History of OPMD: MD Time to OPMD onset: MD Previous history of malignancies: MD Other comorbidities: MD Other ongoing treatments: MD	Macroscopic features: MD Location: MD Staging: Stage I (n.134) Stage II (n.173) Stage III (n.172) Stage IV (n.551) Stage MD (n.2303) Grading: MD Microscopic features: MD First diagnosis: MD Time to onset: MD Chemotherapy: MD Radiotherapy: MD	Sample(s): n.3065 Method(s) of sample collection: MD Microorganisms identification technique: PCR n.1800 IHC n.779 ISH n.264 PCR/IHC/ISH n.N/D Target: MD	↑ HPV: n.672 (prevalence 21.92) of OSCC case Genotype(s) of HPV detected: HPV-16: n.82 HPV-6, -11, -16, -18, -26, -31, -32, -33, -34, -35, -37, -39, -40, -42, -43, -44, -45, -51, -52, -53, 54, -55, -56, -58, -59, -61, -62, -66, -67, -68, -69, -70, -71, -72, -74, -81, -82: n.N/D
de Carvalho Melo, 2021 Braz J Otorhinolaryngol Studies: n.5 CCS n.5 No meta-analysis Low quality No funding	Sample size: n.383 of case Mean age: N/D y.o.; range 19–92 y.o. Gender ratio: 218M/165F Country: USA n.113 Greece n.53 Chile n.80 China n.137 Risk factors for OSCC: Tobacco n.2 of OSCC HPV+ Alcohol n.2 of OSCC HPV+ History of OPMD: MD Time to OPMD onset: MD Previous history of malignancies: MD Other comorbidities: MD Other ongoing treatments: MD	Macroscopic features: MD Location: tongue n.9; dentoalveolar complex n.1; MD n.373. Staging: MD Grading: MD Microscopic features: MD First diagnosis: MD Time to onset: MD Chemotherapy: MD Radiotherapy: MD	Sample(s): n.383 Method(s) of sample collection: Biopsy n.225 MD n.158 Microorganisms identification technique: N/D Target: E6 mRNA n.MD E7 mRNA n.MD	HPV: n.16 of OSCC case Genotype(s) of HPV detected: HPV-16: n.14 HPV-18: n.2 NS
de Lima, 2014 J Bras Patol Med Lab Studies: n.37 MD n.37 No meta-analysis Critically low No funding	Sample size: n.N/D of case/n.N/D of the healthy control group Mean age: MD Gender ratio: MD Country: Japan n.N/D Taiwan n.N/D India n.N/D China n.N/D South Africa n.N/D Sudan n.N/D Finland n.N/D Italy n.N/D Spain n.N/D Germany n.N/D Hungary n.N/D Czech Republic n.N/D The Netherlands n.N/D Serbia n.N/D USA n.N/D Venezuela n.N/D Brazil n.N/D Argentina n.N/D Risk factors for OSCC: Tobacco and alcohol n.36 History of OPMD: MD Time to OPMD onset: MD Previous history of malignancies: MD Other comorbidities: MD Other ongoing treatments: MD	Macroscopic features: MD Location: tongue n.12 Staging: In situ OSCC (n.7) Stage II (n.12) Grading: MD Microscopic features: MD First diagnosis: MD Time to onset: MD Chemotherapy: MD Radiotherapy: MD	Sample(s): n.N/D Method(s) of sample collection: MD Microorganisms identification technique: PCR: n.940 In situ PCR: n.220 Nested PCR: n.290 RT-PCR: n.59 PCR dot blot: n.198 Differential PCR: n.60 IHC: n.635 ISH: n.864 Radioactive ISH: n.117 DNA sequencing: n.64 Single strand conformation polymorphism: n.60 Target: HPV DNA: n.640 p16: n.193 p53: n.496 p21: n.33 pRb: n.112 BCL-2: n.43 hTERT: n.35 C-MYC: n.60 EBV genome: n.601	↑ HPV genome: n.555 of OSCC case Genotype(s) of HPV detected: HPV-6, -11, -16, -18, -31, -33: n.N/D ↑ EBV: n.236 of OSCC case
de Lima, 2019 Crit Rev Oncog Studies: n.52 MD n.52 Meta-analysis Critically low quality No funding	Sample size: n.2665 of case/n. N/D of the healthy control group/n.N/D of the OPMD control group Mean age: MD Gender ratio: MD Country: N/D Risk factors for OSCC: MD History of OPMD: MD Time to OPMD onset: MD Previous history of malignancies: MD Other comorbidities: MD Other ongoing treatments: MD	Macroscopic features: MD Location: MD Staging: MD Grading: MD Microscopic features: MD First diagnosis: MD Time to onset: MD Chemotherapy: MD Radiotherapy: MD	Sample(s): n.2665 Method(s) of sample collection: Biopsy n.2530 Brush n.135 Microorganisms identification technique: PCR: n.1016 In situ PCR: n.102 PCR Southern blot: n.411 RT-PCR Southern blot: n.40 Real-time qPCR: n. 158 qPCR: n.128 Real-time RT-PCR: n.35 Nested PCR: n.708 PCR ELISA: n.79 IF: n.85 IHC: n.721 ISH: n.179 RNA ISH: n.668 Radioactive RNA ISH: n.4 DNA ISH: n.142 RNA microarray: n.151 NASBA: n.9 Target: EBV: n.471 EBNA1: n.458 EBNA2: n.444 LMP gene: n.33 LMP1: n.716 LMP2: n.4 EBER: n.535 EBER1: n.295 BZLF1: n.240 BNLF1: n.127 BNRF1: n.16 BNFR1 n.60 BMLF1: n.16 BHRF1: n.9 BARF0: n.10 IR-1 region: n.36 IR-3 region: n.1 71 EBV fragments: n.57 BamHI W: n.974 BamHI L: n.103 MD n.31	EBV: n.1207 (prevalence 45.29%) of OSCC case *p* = 0.000 Genotype(s) of EBV detected: MD
Guo, 2018 Front Oncol Studies: n.11 MD n.11 Meta-analysis Critically low This study was supported by the National Natural Science Foundation of China (No.81703298).	Sample size: n.1275 of case Mean age: MD Gender ratio: MD Country: China n.1275 Risk factors for OSCC: MD History of OPMD: MD Time to OPMD onset: MD Previous history of malignancies: MD Other comorbidities: MD Other ongoing treatments: MD	Macroscopic features: MD Location: MD Staging: MD Grading: MD Microscopic features: MD First diagnosis: MD Time to onset: MD Chemotherapy: MD Radiotherapy: MD	Sample(s): n.1275 Method(s) of sample collection: Biopsy n.1275 Microorganisms identification technique: PCR: n.921+N/D ISH: n.N/D Target: HPV-16: n.1275	↑ HPV: n.190 (prevalence 14.9%) of OSCC case *p* < 0.001 Genotype(s) of HPV detected: HPV-16: n.190
Haghshenas, 2022 Iran J Public Health Studies: n.2 CCS n.2 Meta-analysis Critically low This study was supported by the Mazandaran University of Medical Sciences (IR.MAZUMS. REC.1399.8657).	Sample size: n.69 of case/n.57 of the healthy control group Mean age: MD Gender ratio: MD Country: MD Risk factors for OSCC: MD History of OPMD: MD Time to OPMD onset: MD Previous history of malignancies: MD Other comorbidities: MD Other ongoing treatments: MD	Macroscopic features: MD Location: MD Staging: MD Grading: MD Microscopic features: MD First diagnosis: MD Time to onset: MD Chemotherapy: MD Radiotherapy: MD	Sample(s): n.69 Method(s) of sample collection: Biopsy n.69 Microorganisms identification technique: N/D Target: MD	Than the healthy control group (prevalence 0.00%) ↑ HPV: n.20 (prevalence 28.99%) of OSCC case Genotype(s) of HPV detected: HPV-16: n.5 HPV-18: n.2 MD n.13
Hobbs, 2006 Clin Otolaryngol Studies: n.15 MD n.15 Meta-analysis Critically low No funding	Sample size: n.1873 of case/n.2437 of the healthy control group Mean age: MD Gender ratio: MD Country: MD Risk factors for OSCC: MD History of OPMD: MD Time to OPMD onset: MD Previous history of malignancies: MD Other comorbidities: MD Other ongoing treatments: MD	Macroscopic features: MD Location: N/D oral cavity n.1656 Tonsil n.217 Staging: MD Grading: MD Microscopic features: MD First diagnosis: MD Time to onset: MD Chemotherapy: MD Radiotherapy: MD	Sample(s): n.1873 Method(s) of sample collection: Biopsy n.322 Serum analysis n.1551 Microorganisms identification technique: N/D Target: N/D	↑ HPV: n.314 (prevalence 16.76%) of OSCC case n.219 (13.22%): N/D oral cavity n.95 (43.78%): tonsil Genotype(s) of HPV detected: N/D
Kreimer, 2005 Cancer Epidemiol Biomarkers Prev Studies: n.38 MD n.38 Meta-analysis Critically low quality No funding	Sample size: n.2642 of case Mean age: MD Gender ratio: MD Country: United Kingdom n.39 Germany n.53 Switzerland n.15 Italy n.38+N/D Slovenia n.55 The Netherlands n.105 France n.12 Spain n.2+N/D Canada n.53 USA n.832 Finland n.28+N/D Norway n.N/D Sweden n.N/D North Ireland n.N/D Poland n.N/D India n.473 Taiwan n.103 Japan n.306 China n.85 Korea n.76 Venezuela n.50 Cuba n.N/D Australia n.N/D Sudan n.N/D Risk factors for OSCC: MD History of OPMD: MD Time to OPMD onset: MD Previous history of malignancies: MD Other comorbidities: MD Other ongoing treatments: MD	Macroscopic features: MD Location: MD Staging: MD Grading: MD Microscopic features: MD First diagnosis: MD Time to onset: MD Chemotherapy: MD Radiotherapy: MD	Sample(s): n.2642 Method(s) of sample collection: Biopsy n.2642 Microorganisms identification technique: PCR n.2642 Target: MD	HPV: n.796 (prevalence 23.5%) of OSCC case HPV prevalence in - Europe: 16.0% - North America: 16.1% - Asia: 33.0% - Other: 18.1% ↓ Than the oropharyngeal SCCs group (prevalence 35.6%) and the laryngeal SCCs group (prevalence 24.0%) Genotype(s) of HPV detected: HPV-6: n.59 (7.41%) HPV-11: n.31 (3.89%) HPV-16: n.423 (53.34%) HPV-18: n.212 (26.73%) HPV-16 and -18: n.44 (5.55%) HPV-31: n.3 (0.38%) HPV-32: n.1 (0.13%) HPV-33: n.14 (1.76%) HPV-35: n.1 (0.13%) HPV-39: n.0 (0.0%) HPV-44: n.1 (0.13%) HPV-45: n.0 (0.0%) HPV-51: n.0 (0.0%) HPV-52: n.0 (0.0%) HPV-53: n.1 (0.13%) HPV-56: n.2 (0.25%) HPV-57: n.1 (0.13%) HPV-58: n.1 (0.13%) HPV-59: n.0 (0.0%) HPV-68: n.1 (0.13%) HPV-73: n.0 (0.0%) HPV-81: n.1 (0.13%) HPV-82: n.0 (0.0%)
Mallika, 2020 Trans Cancer Res Studies: n.8 CCS n.8 No meta-analysis Moderate quality No funding	Sample size: MD Mean age: MD Gender ratio: MD Country: MD Risk factors for OSCC: MD History of OPMD: MD Time to OPMD onset: MD Previous history of malignancies: MD Other comorbidities: MD Other ongoing treatments: MD	Macroscopic features: MD Location: MD Staging: MD Grading: MD Microscopic features: MD First diagnosis: MD Time to onset: MD Chemotherapy: yes (one study) Radiotherapy: yes (two studies)	Sample(s): MD Method(s) of sample collection: Biopsy n.MD Swab n.MD Brush n.MD Microorganisms identification technique: Culture n.MD IHC n.MD ISH n.MD PCR n.MD Target: MD	↑ HPV: MD n./% of OSCC case Genotype(s) of HPV detected: HPV-16 n.MD HPV-18 n.MD HHV-6: NS EBV: NS
Miller, 2001 Oral Surg Oral Med Oral Pathol Oral Radiol Endod Studies: n.80 MD n.80 Meta-analysis Critically low quality No funding	Sample size: n.N/D of case Mean age: MD Gender ratio: MD Country: MD Risk factors for OSCC: MD History of OPMD: MD Time to OPMD onset: MD Previous history of malignancies: MD Other comorbidities: MD Other ongoing treatments: MD	Macroscopic features: MD Location: MD Staging: MD Grading: MD Microscopic features: MD First diagnosis: MD Time to onset: MD Chemotherapy: MD Radiotherapy: MD	Sample(s): n.N/D Method(s) of sample collection: Biopsy n.N/D Microorganisms identification technique: N/D ISH/IP/IF n.608 N/D Southern blot/dot blot/filter blot hybridization n.321 PCR n.1154 Target: HPV DNA: n.2083	↑ HPV: n.627 (prevalence 46.5%) of OSCC case *p* < 0.001 Genotype(s) of HPV detected: HPV-2: n.3 HPV-3: n.1 HPV-4: n.2 HPV-6: n.52 HPV-10: n.16 HPV-11: n.37 HPV-13: n.1 HPV-16: n.285 HPV-18: n.115 HPV-31: n.3 HPV-33: n.9 HPV-57: n.1 HPV-6 and -11: n.27 HPV-16 and -18: n.49 HPV-6, and 11 and 16 and 18: n.1 HPV-6 and -16 and -18: n.3 HPV-31 and -33 and -35: n.6 N/D: 16 LR-HPV: *p* < 0.001 HR-HPV: *p* < 0.001 (prevalence 23.71%)
Muthusamy, 2023 Cureus Studies: n.6 CCS n.6 Meta-analysis Critically low quality This study was supported by the Indian Council of Medical Research (ICMR) under the Nurturing Clinical Scientist (NCS) scheme HRD/Head-NCS-2019-02.	Sample size: n.373 of case/n.326 of healthy control group/n.73 of OPMD group Mean age: MD Gender ratio: MD Country: MD Risk factors for OSCC: MD History of OPMD: MD Time to OPMD onset: MD Previous history of malignancies: MD Other comorbidities: MD Other ongoing treatments: MD	Macroscopic features: MD Location: MD Staging: MD Grading: MD Microscopic features: MD First diagnosis: MD Time to onset: MD Chemotherapy: MD Radiotherapy: MD	Sample(s): n.373 Method(s) of sample collection: Blood analysis n.50 Biopsy n.211 Biofilm sampling n.21 Brush n.91 Microorganisms identification technique: PAP technique n.50 Culture n.21 Nested PCR n.120 ELISA n.132 PCR n.50 Target: Herpes Select-1 n.132 16s RNA n.50	↑ EBV: MD n./% of OSCC case *p* < 0.0001 HSV-1: MD n./% of OSCC case *p* = NSS
Nandi, 2021 *Cancer Treat Res Commun* Studies: n.19 CCS n.19 No meta-analysis Critically low quality No funding	Sample size: n.1639 of case/n.206 of the healthy control group Mean age: N/D y.o.; range MD Gender ratio: 955M/377F/307MD Country: India n.1639 Risk factors for OSCC: MD History of OPMD: MD Time to OPMD onset: MD Previous history of malignancies: MD Other comorbidities: MD Other ongoing treatments: MD	Macroscopic features: MD Location: tongue n.156 MD n.1483 Staging: MD Grading: MD Microscopic features: MD First diagnosis: MD Time to onset: MD Chemotherapy: MD Radiotherapy: MD	Sample(s): n.1639 Method(s) of sample collection: N/D Microorganisms identification technique: PCR/p16-IHC n.216 ISH n.31 IHC n.30 PCR/ISH n.45 PCR n.1317 Target: MD	↑ HPV: n.553 of OSCC case Genotype(s) of HPV detected: MD
Rahman, 2023 Mol Oral Microbiol Studies: n.15 CCS n.5 NRS n.10 Meta-analysis Critically low No funding	Sample size: n.1109 of case/n.211 of the healthy control group/n.150 of the OPMD group Mean age: MD Gender ratio: MD Country: USA n.61 Poland n.53 Sweden n.17 Norway n.20 United Kingdom n.20 Sudan n.20 India n.20 Sri Lanka n.20 Yemen n.18 Risk factors for OSCC: MD History of OPMD: MD Time to OPMD onset: MD Previous history of malignancies: MD Other comorbidities: MD Other ongoing treatments: MD	Macroscopic features: MD Location: tonsil n.16 oral floor n.30 Buccal mucosa n.27 tongue n.74 palate n.16 (soft n.10/MD n.6) lip n.11 MD n.935 Staging: MD Grading: MD Microscopic features: MD First diagnosis: MD Time to onset: MD Chemotherapy: MD Radiotherapy: MD	Sample(s): n.1109 Method(s) of sample collection: Biopsy n.1109 Microorganisms identification technique: PCR n.968 ISH n.141 Target: MD	HPV-EBV coinfection: n.95 of OSCC case *p* < 0.01 HPV: n.31 of OSCC case Genotype(s) of HPV detected: MD
Shaikh, 2015 Cancer Epidemiol Studies: n.45 CSS n.N/D CCS n.N/D Meta-analysis Low quality This study was supported by the Griffith University of Australia.	Sample size: n.4893 of case Mean age: 56.80 y.o.; range 22–94 y.o. Gender ratio: MD Country: India n.1293 Pakistan n.48 Bangladesh n.34 Sri Lanka n.96 Malaysia n.109 Thailand n.32 China n.183 Hong Kong n.31 Taiwan n.535 South Korea n.167 Japan n.843 Australia n.1522 Risk factors for OSCC: Tobacco n.171+N/D Tobacco not smoked n.N/D Alcohol n.N/D History of OPMD: MD Time to OPMD onset: MD Previous history of malignancies: MD Other comorbidities: MD Other ongoing treatments: MD	Macroscopic features: MD Location: buccal mucosa n.433 gingiva n.196 tongue n.679 oral floor n.96 palate n.64 (hard n.1/soft n.5/MD n.59) tonsil n.1587 lip n.114 N/D oral cavity n.2353 Staging: MD Grading: MD Microscopic features: MD First diagnosis: MD Time to onset: MD Chemotherapy: MD Radiotherapy: MD	Sample(s): n.4893 Method(s) of sample collection: Biopsy n.4893 Microorganisms identification technique: PCR n.4209 Southern blot PCR n.224 Slot blot PCR n.15 Nested PCR n.27 ISH n.102 PCR and ISH: n.244 RT-PCR and IHC: n.52 In situ PCR and ISH n.20 Target: HPV genome: n.N/D	↑ HPV: n.1627 of OSCC case n.146 (33.72%): buccal mucosa n.59 (30.10%): gingiva n.219 (32.25%): tongue n.29 (30.21%): oral floor n.20 (31.25%): palate n.489 (30.81%): tonsil n.19 (16.67%): lip n.646 (27.45%): N/D oral cavity Genotype(s) of virus detected: HPV-6, -8, -11, -16, -18, -22, -31, -32, -33, -35, -38, -39, -44, -45, -51, -52, -53, -54, -58, -59, -61, -66, -67, -68, -69, -70, -75, -76: n.N/D
She, 2017 PLoS One Studies: n.13 CCS n.13 Meta-analysis Moderate quality No funding	Sample size: n.686 of case/n. 412 of the healthy control group Mean age: MD Gender ratio: MD Country: South Africa n.138 Netherland n.36 China n.81 India n.103 Japan n.186 Sweden n.29 Egypt n.22 Spain n.5 Hungary n.65 USA n.21 Risk factors for OSCC: MD History of OPMD: MD Time to OPMD onset: MD Previous history of malignancies: MD Other comorbidities: MD Other ongoing treatments: MD	Macroscopic features: MD Location: MD Staging: MD Grading: MD Microscopic features: MD First diagnosis: MD Time to onset: MD Chemotherapy: MD Radiotherapy: MD	Sample(s): n.686 Method(s) of sample collection: Biopsy n.686 Microorganisms identification technique: PCR n.562 Nested PCR n.34 ISH n.36 IHC n.33 RT-qPCR n.21 Target: EBV DNA n.218 EBV DNA BamHIW n.275 EBV DNA EBNA2 n.150 EBV RNA EBER1 n.21 EBV protein n.22	↑ EBV: n.332 of OSCC case *p* = 0.002
Sivakumar, 2020 Transl Cancer Res Studies: n.7 CCS n.7 Meta-analysis Low quality No funding	Sample size: n.349 of case/n.205 of the healthy control group Mean age: MD Gender ratio: MD Country: MD Risk factors for OSCC: MD History of OPMD: MD Time to OPMD onset: MD Previous history of malignancies: MD Other comorbidities: MD Other ongoing treatments: MD	Macroscopic features: MD Location: MD Staging: MD Grading: MD Microscopic features: MD First diagnosis: MD Time to onset: MD Chemotherapy: MD Radiotherapy: MD	Sample(s): n.349 Method(s) of sample collection: Biopsy n.349 Microorganisms identification technique: IHC n.61 ISH n.20 PCR n.190 N/D n.78 Target: LMP-1 n.61 EBV DNA n.60 EBNA-2 n.150 N/D n.349	Than the healthy control group (prevalence 20.0%) ↑ EBV: n.161 (prevalence 46.13%) of OSCC case
Syrjänen, 2011 Oral Dis Studies: n.33 CCS n.33 Meta-analysis Critically low quality No funding	Sample size: n.1885 of case/n.2248 of the healthy control group Mean age: MD Gender ratio: MD Country: MD Risk factors for OSCC: MD History of OPMD: MD Time to OPMD onset: MD Previous history of malignancies: MD Other comorbidities: MD Other ongoing treatments: MD	Macroscopic features: MD Location: MD Staging: MD Grading: MD Microscopic features: MD First diagnosis: MD Time to onset: MD Chemotherapy: MD Radiotherapy: MD	Sample(s): n.1885 Method(s) of sample collection: Brush n.697 Biopsy n.1043 Biopsy and Brush n.145 Microorganisms identification technique: PCR n.1821 ISH n.64 Target: MD	HPV: n.634 of OSCC case *p* < 0.00001 Genotype(s) of virus species detected: HPV-16: n.222 *p* < 0.00001
Termine, 2008 Ann Oncol Studies: n.47 MD n.47 Meta-analysis Critically low quality No funding	Sample size: n.3238 of case Mean age: MD Gender ratio: MD Country: Finland n.91 USA n.1082 Spain n.42 Japan n.606 Taiwan n.29 Brazil n.69 Slovenia n.62 South Africa n.271 The Netherlands n.35 Sudan n.88 Hungary n.79 Korea n.42 Venezuela n.116 Greece n.100 China n.113 Sweden n.134 India n.213 Italy n.66 Risk factors for OSCC: MD History of OPMD: MD Time to OPMD onset: MD Previous history of malignancies: MD Other comorbidities: MD Other ongoing treatments: MD	Macroscopic features: MD Location: MD Staging: MD Grading: MD Microscopic features: MD First diagnosis: MD Time to onset: MD Chemotherapy: MD Radiotherapy: MD	Sample(s): n.3238 Method(s) of sample collection: Biopsy n.3238 Microorganisms identification technique: ISH: n.370 PCR: n.2681 N/D ISH or PCR: n.187 Target: MD	↑ HPV: n. 1090 of OSCC case *p* = 0.000 Genotype(s) of HPV detected: HPV-6, -11, -16, -18, -22, -31, -33, -35, -38, -58, -68, -70: n.MD
Yang, 2019 Medicine (Baltimore) Studies: n.6 CSS n.6 Meta-analysis Moderate quality No funding	Sample size: n.758 of case Mean age: MD Gender ratio: MD Country: China n.758 Risk factors for OSCC: MD History of OPMD: MD Time to OPMD onset: MD Previous history of malignancies: MD Other comorbidities: MD Other ongoing treatments: MD	Macroscopic features: MD Location: MD Staging: Stage I-IV (n.327) Stage III-IV (n.333) Stage N/D (n.98) Grading: MD Microscopic features: MD First diagnosis: MD Time to onset: MD Chemotherapy: MD Radiotherapy: MD	Sample(s): n.758 Method(s) of sample collection: Biopsy n.758 Microorganisms identification technique: PCR n.704 ISH n.54 Target: L1 region of HPV-18 gene n.533	HPV: n.55/7.2% of OSCC case Type(s) of HPV detected: HPV-18: n.55 *p* = 0.011
Zhu, 2012 PLoS One Studies: n.18 CCS n.18 Meta-analysis Critically low quality No funding	Sample size: n.610 of case/n.259 of the healthy control group Mean age: MD y.o.; range MD Gender ratio: MD Country: China n.610 Risk factors for OSCC: MD History of OPMD: MD Time to OPMD onset: MD Previous history of malignancies: MD Other comorbidities: MD Other ongoing treatments: MD	Macroscopic features: MD Location: MD Staging: MD Grading: MD Microscopic features: MD First diagnosis: MD Time to onset: MD Chemotherapy: MD Radiotherapy: MD	Sample(s): n.610 Method(s) of sample collection: MD Microorganisms identification technique: PCR n.544 Dot-blot hybridization n.66 Target: MD	Than the healthy control group ↑ HPV: n.354 of OSCC case *p* < 0.00001 Type(s) of HPV detected: HPV-16: n.169 *p* < 0.00001 MD: n.185

Abbreviations: number, “n.”; years old, “y.o.”; percentage, “%”; greater than, “>”; missing data, “MD”; not defined, “N/D”; not statistically significant, “NSS”; not significant, “NS”; *p*-value, “*p*”; male, “M”; female, “F”; case-control study, “CCS”; cohort study, “CS”; cross-sectional study, “CSS”; retrospective cross-sectional study, “RCSS”; prospective study, “PS”; non-randomized study, “NRS”; oral squamous cell carcinoma, “OSCC”; oral potentially malignant disorder, “OPMD”; United States of America, “USA”; polymerase chain reaction, “PCR”; real time polymerase chain reaction, “RT-PCR”; quantitative polymerase chain reaction, “qPCR”; immunohistochemistry, “IHC”; in situ hybridization, “ISH”; enzyme-linked immunosorbent assay, “ELISA”; immunofluorescence, “IF”; immunoperoxidase, “IP”; nucleic acid sequence based amplification, “NASBA”; Papanicolaou technique, “PAP technique”; Messenger Ribonucleic Acid, “mRNA”; Ribosomal Ribonucleic Acid, “rRNA”; RiboNucleic Acid, “RNA”; DeoxyriboNucleic Acid, “DNA”; Ribosomal DeoxyriboNucleic Acid, “rDNA”; subspecies, “ssp.”; Fusobacterium, “F.”; Campylobacter, “C.”; Streptococcus, “S.”; restriction fragment length polymorphism, “RFLP”; Human Papilloma Virus, “HPV”; Low Risk Human Papilloma Virus, “LR-HPV”; High Risk Human Papilloma Virus, “HR-HPV”; Herpes Simplex Virus, “HSV”; Epstein Barr Virus, “EBV”; Epstein Barr Nuclear Antigen, “EBNA”; Epstein Barr Encoding Region, “EBER”; Latent Membrane Protein, “LMP”; human telomerase reverse transcriptase, “hTERT”; B-cell lymphoma 2, “BCL-2”; Bahm HI N fragment rightward open reading frame, “BNRF”; Bahm HI A fragment rightward open reading frame, “BARF”; Bahm HI H fragment rightward open reading frame, “BHRF”; Bahm HI N fragment leftward open reading frame, “BZLF”; BahmHI M fragment leftward open reading frame, “BMLF”; BahmHI Z fragment leftward open reading frame, “BZLF”; increased, “↑”; decreased, “↓”.

**Table 3:** Data primarily concerning fungi extracted and collected from the included studies that had carried out a histopathologic analysis of OSCC-tissue lesions. Studies: first author, year and journal of publication, reference, number and design of included studies, meta-analysis, assessed quality, funding (if any). Population characteristics: sample size, mean age, gender ratio, country of origin of the sample, risk factor and history of OPMD, history of malignancies, other comorbidities, and ongoing treatments. OSCC characteristics: macroscopic features, location, staging and grading, microscopic features, first diagnosis (primary site/metastatic lesion), time to onset, chemotherapy (yes/no), radiotherapy (yes/no). Intervention: number of sample(s), method(s) of sample collection, microorganisms identification technique, target. Outcome(s): type(s) and species of fungus detected, number or percentage of OSCC-positive cases.

Studies	Population		Intervention	Outcome(s)
	Characteristics	OSCC		Fungi
Ayuningtyas, 2022 Pathophysiology Studies: n.5 RCSS n.2 CSS n.2 PS n.1 No Meta-analysis Critically low quality No funding	Sample size: n.136+MD of case/n.92+MD of the healthy control group/n.107+MD of OPMD control group Mean age: MD Gender ratio: MD Country: India n.80 Egypt n.31 Argentina n.25 Taiwan n.MD Risk factors for OSCC: MD History of OPMD: MD Time to OPMD onset: MD Previous history of malignancies: MD Other comorbidities: MD Other ongoing treatments: MD	Macroscopic features: MD Location: MD Staging: MD Grading: MD Microscopic features: MD First diagnosis: MD Time to onset: MD Chemotherapy: MD Radiotherapy: MD	Sample(s): n.136+MD Method(s) of sample collection: Biopsy n.61 Swab n.50 Biopsy and Swab n.25+MD Microorganisms identification technique: Culture n.136+MD IHC: n.MD Target: MD	Than the healthy control group ↑ Candida: n.MD of OSCC case Than the OPMD control group ↑ Candida: n.MD of OSCC case Type(s) of fungi species detected: MD
Mallika, 2020 Trans Cancer Res Studies: n.8 CCS n.8 No meta-analysis Moderate quality No funding	Sample size: MD Mean age: MD Gender ratio: MD Country: MD Risk factors for OSCC: MD History of OPMD: MD Time to OPMD onset: MD Previous history of malignancies: MD Other comorbidities: MD Other ongoing treatments: MD	Macroscopic features: MD Location: MD Staging: MD Grading: MD Microscopic features: MD First diagnosis: MD Time to onset: MD Chemotherapy: yes (one study) Radiotherapy: yes (two studies)	Sample(s): MD Method(s) of sample collection: Biopsy n.MD Swab n.MD Brush n.MD Microorganisms identification technique: Culture n.MD IHC n.MD ISH n.MD PCR n.MD Target: MD	↑ Oral yeast: MD n./% of OSCC case Candida: MD n./% of OSCC case (two studies of OSCC patients undergoing radiotherapy; one study of OSCC patients undergoing chemotherapy) Type(s) of fungi species detected: MD

Abbreviations: number, “n.”; years old, “y.o.”; percentage, “%”; greater than, “>”; missing data, “MD”; not defined, “N/D”; not statistically significant, “NSS”; not significant, “NS”; *p*-value, “*p*”; male, “M”; female, “F”; case-control study, “CCS”; cohort study, “CS”; cross-sectional study, “CSS”; retrospective cross-sectional study, “RCSS”; prospective study, “PS”; non-randomized study, “NRS”; oral squamous cell carcinoma, “OSCC”; oral potentially malignant disorder, “OPMD”; United States of America, “USA”; polymerase chain reaction, “PCR”; real time polymerase chain reaction, “RT-PCR”; quantitative polymerase chain reaction, “qPCR”; immunohistochemistry, “IHC”; in situ hybridization, “ISH”; enzyme-linked immunosorbent assay, “ELISA”; immunofluorescence, “IF”; immunoperoxidase, “IP”; nucleic acid sequence based amplification, “NASBA”; Papanicolaou technique, “PAP technique”; Messenger Ribonucleic Acid, “mRNA”; Ribosomal Ribonucleic Acid, “rRNA”; RiboNucleic Acid, “RNA”; DeoxyriboNucleic Acid, “DNA”; Ribosomal DeoxyriboNucleic Acid, “rDNA”; subspecies, “ssp.”; Fusobacterium, “F.”; Campylobacter, “C.”; Streptococcus, “S.”; restriction fragment length polymorphism, “RFLP”; Human Papilloma Virus, “HPV”; Low Risk Human Papilloma Virus, “LR-HPV”; High Risk Human Papilloma Virus, “HR-HPV”; Herpes Simplex Virus, “HSV”; Epstein Barr Virus, “EBV”; Epstein Barr Nuclear Antigen, “EBNA”; Epstein Barr Encoding Region, “EBER”; Latent Membrane Protein, “LMP”; human telomerase reverse transcriptase, “hTERT”; B-cell lymphoma 2, “BCL-2”; Bahm HI N fragment rightward open reading frame, “BNRF”; Bahm HI A fragment rightward open reading frame, “BARF”; Bahm HI H fragment rightward open reading frame, “BHRF”; Bahm HI N fragment leftward open reading frame, “BZLF”; BahmHI M fragment leftward open reading frame, “BMLF”; BahmHI Z fragment leftward open reading frame, “BZLF”; increased, “↑”.

**Table 4:** Data primarily concerning bacteria extracted and collected from the included studies that had carried out saliva testing in OSCC adult subjects. Studies: first author, year and journal of publication, reference, number and design of included studies, meta-analysis, assessed quality, funding (if any). Population characteristics: sample size, mean age, gender ratio, country of origin of the sample, risk factor and history of OPMD, history of malignancies, other comorbidities, and ongoing treatments. OSCC characteristics: macroscopic features, location, staging and grading, microscopic features, first diagnosis (primary site/metastatic lesion), time to onset, chemotherapy (yes/no), radiotherapy (yes/no). Intervention: number of sample(s), method(s) of sample collection, microorganisms identification technique, target. Outcome(s): type(s) of phylum, genus and species of bacterium detected, number or percentage of OSCC-positive cases.

Studies	Population		Intervention	Outcome(s)
	Characteristics	OSCC		Bacteria
Gopinath, 2019 Crit Rev Oncol Hematol Studies: n.2 CCS n.2 No meta-analysis Low quality No funding	Sample size: n.130 of case/n.125 of the healthy group control Mean age: >50 y.o.; range MD Gender ratio: MD Country: Taiwan n.127 USA n.3 Risk factors for OSCC: N/D History of OPMD: MD Time to OPMD onset: MD Previous history of malignancies: MD Other comorbidities: MD Other ongoing treatments: MD	Macroscopic features: MD Location: MD Staging: MD Grading: MD Microscopic features: MD First diagnosis: MD Time to onset: MD Chemotherapy: MD Radiotherapy: MD	Sample(s): n.130 Method(s) of sample collection: Saliva test n.130 Microorganisms identification technique: Incubation in Proteinase K and DNA purification kit n.3 QIAamp DNA Blood Kit n.127 Target: V4-V5 region n.3 V4 region n.127	Than the healthy control group Bacteroidetes: ↑ Bacteroides: >n.50 of OSCC case ↑ Porphyromonas Proteobacteria: ↑ Escherichia: >n.50 of OSCC case Ralstonia Type(s) of Ralstonia species detected: ↑ Ralstonia insidiosa Actinobacteria: ↑ Rothia Firmicutes: ↑ Bulleidia: >n.50 of OSCC case ↑ Gemella ↑ Peptostreptococcus ↑ Streptococcus ↑ Lactobacillus ↑ Gemmiger ↑ Oscillospira ↑ RoseburiaSynergistota: ↑ Cloacibacillus
Gupta, 2020 Clin Oral Investig Studies: n.1 CSS n.1 No meta-analysis Low quality No funding	Sample size: n.50 of case/n.50 of the healthy control group Mean age: MD Gender ratio: MD Country: India n.50 Risk factors for OSCC: MD History of OPMD: MD Time to OPMD onset: MD Previous history of malignancies: MD Other comorbidities: MD Other ongoing treatments: MD	Macroscopic features: MD Location: MD Staging: MD Grading: MD Microscopic features: MD First diagnosis: MD Time to onset: MD Chemotherapy: MD Radiotherapy: MD	Sample(s): n.50 Method(s) of sample collection: Saliva test n.50 Microorganisms identification technique: Culture n.50 Target: MD	Than the healthy control group (prevalence 8.0%) Proteobacteria: Helicobacter Type(s) of Helicobacter species detected: ↑ Helicobacter pylori: n.32 (prevalence 64.0%) of OSCC case
Huybrechts, 2020 Cancer Epidemiol Biomarkers Prev Studies: n.16 CS n.2 CCS n.14 No meta-analysis Low quality This study was supported by the Intramural Research Program of the National Cancer Institute at the National Institutes of Health and by the Research Foundation-Flanders 12h1519N.	Sample size: n.724 of case/n.1188 of the healthy control group Mean age: MD Gender ratio: MD Country: MD Risk factors for OSCC: MD History of OPMD: MD Time to OPMD onset: MD Previous history of malignancies: MD Other comorbidities: MD Other ongoing treatments: MD	Macroscopic features: MD Location: MD Staging: MD Grading: MD Microscopic features: MD First diagnosis: MD Time to onset: MD Chemotherapy: MD Radiotherapy: MD	Sample(s): n.724 Method(s) of sample collection: Saliva test n.724 Microorganisms identification technique: N/D Target: MD	↑ Bacteroidetes: ↑ Capnocytophaga ↑ Fusobacteria: ↑ Fusobacterium Actinobacteria: ↑ Actinomyces ↓ Firmicutes: ↑ Dialister ↑ Peptostreptococcus ↑ Parvimonas ↑ Streptococcus: NS
Mallika, 2020 Trans Cancer Res Studies: n.11 CCS n.11 No meta-analysis Moderate quality No funding	Sample size: MD Mean age: MD Gender ratio: MD Country: MD Risk factors for OSCC: MD History of OPMD: MD Time to OPMD onset: MD Previous history of malignancies: MD Other comorbidities: MD Other ongoing treatments: MD	Macroscopic features: MD Location: MD Staging: MD Grading: MD Microscopic features: MD First diagnosis: MD Time to onset: MD Chemotherapy: MD Radiotherapy: MD	Sample(s): MD Method(s) of sample collection: Saliva test n.MD Oral swab n.MD Microorganisms identification technique: Culture n.MD VIDAS EBV kit n.MD QIAamp Mini Elute Virus Spin kit Digene HPV genotyping RH test n.MD PCR n.MD Spectrophotometer n.MD Gas chromatography n.MD IHC n.MD Target: MD	Proteobacteria: Helicobacter Type(s) of Helicobacter species detected: ↑ Helicobacter pylori
Mauceri, 2022 Cancers (Basel) Studies: n.11 CCS n.8 CS n.2 CSCS n.1 No meta-analysis Critically low quality No funding	Sample size: n.679 of case/n.480 of the healthy control group/n.153 of the OPMD control group/n.15 of the periodontitis control group Mean age: 56.8 y.o.; range 49.7–63.9 y.o. Gender ratio: 179M/113F/387MD Country: Taiwan n.445 China n.97 USA n.18 Sudan n.59 Japan n.60 Risk factors for OSCC: MD History of OPMD: MD Time to OPMD onset: MD Previous history of malignancies: MD Other comorbidities: MD Other ongoing treatments: MD	Macroscopic features: MD Location: MD Staging: MD Grading: MD Microscopic features: MD First diagnosis: MD Time to onset: MD Chemotherapy: MD Radiotherapy: MD	Sample(s): n.687 Method(s) of sample collection: Sputum n.448 Oral swab n.156 Oral rinse n.18 Saliva, subgingival plaque, tumor and healthy surface n.57 Microorganisms identification technique: QIAamp DNA Blood Mini kit n.347 QIAamp MinElute Virus Spin kit n.138 QIAamp Fast DNA Stool Mini kit n.10 FastDNA kit n.59 Gene Prep Star PI-80X device n.60 Modified QIAGEN DNA extraction method n.18 E.Z.N.A. soil DNA kit n.47 Target: Fungal ITS2 region n.59 V4 region n.280 V3-V4 region n.115 V3-V5 region n.138 V4-V5 region n.87	↑ Spirochaetes: ↑ Treponema ↑ Proteobacteria: ↑ Campylobacter ↓ Lautropia ↓ Haemophilus ↑ Eikenella ↑ Bacteroidetes: ↑ Alloprevotella ↑ Capnocytophaga ↑ PrevotellaTenericutes: ↑ Mycoplasma ↓ Firmicutes: MD n./% of OSCC case ↑ Centipeda ↑ Selenomonas ↑ Dialister ↑ Peptostreptococcus ↑ Filifactor ↑ Peptococcus ↑ Catonella ↑ Parvimonas ↓ Megasphaera ↓ Stomatobaculum ↓ Granulicatella ↓ Veillonella ↓ Streptococcus Type(s) of Streptococcus species detected: ↓ S. pneumoniae ↑ Fusobacteria: ↑ Fusobacterium Type(s) of Fusobacterium species detected: F. nucleatum ↓ Actinobacteria: MD n./% of OSCC case ↓ Scardovia ↓ Rothia ↓ Actinomyces
Ramos, 2020 Oral Maxillofac Surg Studies: n.4 CS n.1 CSS n.1 CCS n.1 MD n.1 No meta-analysis Critically low quality This study was supported by the National Council for Scientific and Technological Development (CNPq) (Project:211309/ 2013-3) and the Foundation for Research Financial Support in the State of Rio de Janeiro (FAPERJ) (Project: E26/ 1033.001/2012).	Sample size: n.339 of case/n. 214 of the healthy control group/n.124 of the OPMD control group Mean age: MD Gender ratio: N/D Country: MD Risk factors for OSCC: Tobacco: n.N/D Alcohol: n.N/D Betel quid chewing: n.N/D History of OPMD: MD Time to OPMD onset: MD Previous history of malignancies: MD Other comorbidities: MD Other ongoing treatments: MD	Macroscopic features: MD Location: MD Staging: Stage I (n.41) Stage II (n.66) Stage IV (n.90) Grading: MD Microscopic features: MD First diagnosis: MD Time to onset: MD Chemotherapy: MD Radiotherapy: MD	Sample(s): n.339 Method(s) of sample collection: Oral rinse n.197 Saliva test n.142 Microorganisms identification technique: MiSeq n.333 454/GS Junior n.6 Target: V4 n.125 V3-V4 n.197 V3-V5 n.6 V4-V5 n.11	Than the healthy control group Firmicutes: ↑ Streptococcus Type(s) of Streptococcus species detected: ↑ S. mitis (in smokers patients) ↑ Peptostreptococcus ↑ Bacillus ↑ Parvimonas ↑ Enterococcus ↑ Veillonella ↑ Stomatobaculum ↑ Lactobacillus (abundance with advanced TNM stage) Bacteroidetes: ↑ Prevotella ↑ Proteobacteria: ↑ Haemophilus Actinobacteria: ↑ Slackia ↑ RothiaTenericutes: ↑ Mollicutes Spirochaetes: ↑ Spirochaetales
Shen, 2023 Arch Oral Biol Studies: n.5 CCS n.5 No meta-analysis High quality This study was supported by the China-Japan Friendship Hospital Research Project Foundation [grant number 2020-1-QN-2].	Sample size: n.230 of case/n.219 of the healthy control group/n.205 of the OPMD control group Mean age: N/D Gender ratio: 27M/203MD Country: USA n.18 India n.31 Taiwan n.N/D China n.29+N/D Risk factors for OSCC: MD History of OPMD: MD Time to OPMD onset: MD Previous history of malignancies: MD Other comorbidities: MD Other ongoing treatments: MD	Macroscopic features: MD Location: MD Staging: MD Grading: MD Microscopic features: MD First diagnosis: MD Time to onset: MD Chemotherapy: MD Radiotherapy: MD	Sample(s): n.230 Method(s) of sample collection: Saliva test n.152 Oral rinse n.49 Oral swab, plaque swab, and saliva test n.29 Microorganisms identification technique: Modified QIAGEN DNA n.18 Gene Fix Saliva Prep 2 Isolation kit n.31 QIAamp DNA Blood mini kit n.124 QIAamp DNA Mini kit n.21 HiPure tissue and blood DNA kit n.32 Target: 16s rRNA: n.177 V4 region n.124 V3-V4 region n.103 ITS1 n.32	Than the healthy and the OPMD control group ↑ Bacteroidetes: ↑ Prevotella ↑ Alloprevotella ↑ Porphyromonas ↑ Capnocytophaga Type(s) of Capnocytophaga species detected: ↑ Capnocytophaga sputigena Fusobacteria: ↑/↓ Fusobacterium ↑/↓ Firmicutes: ↓ Streptococcus ↑/↓ Veillonella Catonella Type(s) of Catonella species detected: ↑ Catonella morbi Proteobacteria: ↑ Aggregatibacter ↑ Neisseria Than the OPMD control group Bacteroidetes: ↓ Prevotella Type(s) of Prevotella species detected: ↓ Prevotella oulorum Tannerella Type(s) of Tannerella species detected: ↓ Tannerella forsythia Porphyromonas Type(s) of Porphyromonas species detected: ↓ Porphyromonas gingivalis Firmicutes: ↓ Enterococcus ↓ Megasphaera Anaeroglobus Type(s) of Anaeroglobus species detected: ↓ Anaeroglobus geminatus Proteobacteria: ↓ Salmonella ↓ Saccharibacteria Than the healthy control group Proteobacteria: ↑ Escherichia Firmicutes: ↑ Gemmiger ↑ Oscillospira ↑ Roseburia ↑ Dialister Synergistota: ↑ Claocibacillus

Abbreviations: number, “n.”; years old, “y.o.”; percentage, “%”; greater than, “>”; less than, “<“; missing data, “MD”; not defined, “N/D”; not statistically significant, “NSS”; not significant, “NS”; *p*-value, “*p*”; male, “M”; female, “F”; case-control study, “CCS”; cohort study, “CS”; cross-sectional study, “CSS”; cross-sectional cohort study, “CSCS”; oral squamous cell carcinoma, “OSCC”; oral potentially malignant disorder, “OPMD”; United States of America, “USA”; polymerase chain reaction, “PCR”; real time polymerase chain reaction, “RT-PCR”; quantitative polymerase chain reaction, “qPCR”; immunohistochemistry, “IHC”; Ribosomal Ribonucleic Acid, “rRNA”; RiboNucleic Acid, “RNA”; DeoxyriboNucleic Acid, “DNA”; subspecies, “ssp”; Fusobacterium, “F.”; Campylobacter, “C.”; Streptococcus, “S.”; Human Papilloma Virus, “HPV”; Low Risk Human Papilloma Virus, “LR-HPV”; High Risk Human Papilloma Virus, “HR-HPV”; Epstein Barr Virus, “EBV”; increased, “↑”; decreased, “↓”.

**Table 5:** Data primarily concerning viruses extracted and collected from the included studies that had carried out saliva testing in OSCC adult subjects. Studies: first author, year and journal of publication, reference, number and design of included studies, meta-analysis, assessed quality, funding (if any). Population characteristics: sample size, mean age, gender ratio, country of origin of the sample, risk factor and history of OPMD, history of malignancies, other comorbidities, and ongoing treatments. OSCC characteristics: macroscopic features, location, staging and grading, microscopic features, first diagnosis (primary site/metastatic lesion), time to onset, chemotherapy (yes/no), radiotherapy (yes/no). Intervention: number of sample(s), method(s) of sample collection, microorganisms identification technique, target. Outcome(s): type(s) and genotype of virus detected, number or percentage of OSCC-positive cases.

Studies	Population		Intervention	Outcome(s)
	Characteristics	OSCC		Viruses
de Lima, 2019 Crit Rev Oncog Studies: n.1 MD n.1 Meta-analysis Critically low quality No funding	Sample size: n.12 of case/n.47 of the healthy control group/n.12 of the OPMD control group Mean age: MD Gender ratio: MD Country: MD Risk factors for OSCC: MD History of OPMD: MD Time to OPMD onset: MD Previous history of malignancies: MD Other comorbidities: MD Other ongoing treatments: MD	Macroscopic features: MD Location: MD Staging: MD Grading: MD Microscopic features: MD First diagnosis: MD Time to onset: MD Chemotherapy: MD Radiotherapy: MD	Sample(s): n.12 Method(s) of sample collection: Saliva test n.12 Microorganisms identification technique: Nested PCR n.12 Target: EBV n.12	EBV: n.7 (prevalence 58.3%) of OSCC case ↑ Than the healthy control group (prevalence 40.4%) and the OPMD control group (prevalence 41.7%) Genotype(s) of virus detected: MD
Mallika, 2020 Trans Cancer Res Studies: n.11 CCS n.11 No meta-analysis Moderate quality No funding	Sample size: MD Mean age: MD Gender ratio: MD Country: MD Risk factors for OSCC: MD History of OPMD: MD Time to OPMD onset: MD Previous history of malignancies: MD Other comorbidities: MD Other ongoing treatments: MD	Macroscopic features: MD Location: MD Staging: MD Grading: MD Microscopic features: MD First diagnosis: MD Time to onset: MD Chemotherapy: MD Radiotherapy: MD	Sample(s): MD Method(s) of sample collection: Saliva test n.MD Oral swab n.MD Microorganisms identification technique: Culture n.MD VIDAS EBV kit n.MD QIAamp Mini Elute Virus Spin kit Digene HPV genotyping RH test n.MD PCR n.MD Spectrophotometer n.MD Gas chromatography n.MD IHC n.MD Target: MD	HPV: MD n./% of OSCC case Genotype(s) of HPV detected: ↓ HPV-16 n.MD ↓ HPV-18: NS n.MD EBV: NS Than the healthy control group ↑ EBV
Rapado-González, 2020 J Clin Med Studies: n.12 CCS n.12 Meta-analysis High quality No funding	Sample size: n.658 of case/n.2210 of the healthy control group Mean age: MD Gender ratio: MD Country: Sweden n.85 Iran n.22 India n.313 USA n.109 France n.22 Canada n.72 Pakistan n.35 Risk factors for OSCC: MD History of OPMD: MD Time to OPMD onset: MD Previous history of malignancies: MD Other comorbidities: MD Other ongoing treatments: MD	Macroscopic features: MD Location: MD Staging: MD Grading: MD Microscopic features: MD First diagnosis: MD Time to onset: MD Chemotherapy: MD Radiotherapy: MD	Sample(s): n.658 Method(s) of sample collection: Oral rinse n.286 Saliva test n.372 Microorganisms identification technique: PCR: n.385 qPCR: n.144 Nested PCR: n.44 Nested PCR and DNA sequencing: n.85 Target: HPV-6, -10, -11, -13, -26, -31, -32, -33, -34, -35, -39, -40, -42, -44, -45, -51, -52, -53, -54, -56, -58, -59, -61, -62, -66, -67, -68, -69, -70, -71, -72, -73, -76, -81, -82, -83, -84, -89 genome: n.538 of HR-HPV/ n.107 of LR-HPV HPV-16 genome: n.507 HPV-18 genome: n.254	↑ HPV: n.271 of OSCC case *p* < 0.01 Genotype(s) of virus detected: HPV-16: n.57 *p* < 0.02 HPV-18: n.24 NSS N/D HR-HPV: n.164 *p* < 0.01 N/D LR-HPV: n.8 NSS
Shaikh, 2015 Cancer Epidemiol Studies: n.1 N/D n.1 Meta-analysis Low quality This study was supported by the Griffith University of Australia.	Sample size: n.34 of case/n.396 of control Mean age: MD Gender ratio: MD Country: India n.34 Risk factors for OSCC: History of OPMD: MD Time to OPMD onset: MD Previous history of malignancies: MD Other comorbidities: MD Other ongoing treatments: MD	Macroscopic features: MD Location: MD Staging: MD Grading: MD Microscopic features: MD First diagnosis: MD Time to onset: MD Chemotherapy: MD Radiotherapy: MD	Sample(s): n.34 Method(s) of sample collection: Saliva test Microorganisms identification technique: PCR n.34 Target: HPV genome: n.34	↑ HPV: n.24 (prevalence 70.60%) of OSCC case Genotype(s) of HPV detected: HPV-16: n.MD HPV-18: n.MD

Abbreviations: number, “n.”; years old, “y.o.”; percentage, “%”; greater than, “>”; less than, “<“; missing data, “MD”; not defined, “N/D”; not statistically significant, “NSS”; not significant, “NS”; *p*-value, “*p*”; male, “M”; female, “F”; case-control study, “CCS”; cohort study, “CS”; cross-sectional study, “CSS”; cross-sectional cohort study, “CSCS”; oral squamous cell carcinoma, “OSCC”; oral potentially malignant disorder, “OPMD”; United States of America, “USA”; polymerase chain reaction, “PCR”; real time polymerase chain reaction, “RT-PCR”; quantitative polymerase chain reaction, “qPCR”; immunohistochemistry, “IHC”; Ribosomal Ribonucleic Acid, “rRNA”; RiboNucleic Acid, “RNA”; DeoxyriboNucleic Acid, “DNA”; subspecies, “ssp”; Fusobacterium, “F.”; Campylobacter, “C.”; Streptococcus, “S.”; Human Papilloma Virus, “HPV”; Low Risk Human Papilloma Virus, “LR-HPV”; High Risk Human Papilloma Virus, “HR-HPV”; Epstein Barr Virus, “EBV”; increased, “↑”; decreased, “↓”.

**Table 6:** Data primarily concerning fungi extracted and collected from the included studies that had carried out saliva testing in OSCC adult subjects. Studies: first author, year and journal of publication, reference, number and design of included studies, meta-analysis, assessed quality, funding (if any). Population characteristics: sample size, mean age, gender ratio, country of origin of the sample, risk factor and history of OPMD, history of malignancies, other comorbidities, and ongoing treatments. OSCC characteristics: macroscopic features, location, staging and grading, microscopic features, first diagnosis (primary site/metastatic lesion), time to onset, chemotherapy (yes/no), radiotherapy (yes/no). Intervention: number of sample(s), method(s) of sample collection, microorganisms identification technique, target. Outcome(s): type(s) and species of fungus detected, number or percentage of OSCC-positive cases.

Studies	Population		Intervention	Outcome(s)
	Characteristics	OSCC		Fungi
Ayuningtyas, 2022 Pathophysiology Studies: n.4 CCS n.1 CSS n.3 No meta-analysis Critically low quality No funding	Sample size: n.301 of case/n.408 of the healthy control group/n.200 of the OPMD control group/n.6 of other malignancy control group Mean age: MD Gender ratio: MD Country: India n.97 Australia n.104 Finland n.100 Risk factors for OSCC: MD History of OPMD: MD Time to OPMD onset: MD Previous history of malignancies: MD Other comorbidities: MD Other ongoing treatments: MD	Macroscopic features: MD Location: MD Staging: MD Grading: MD Microscopic features: MD First diagnosis: MD Time to onset: MD Chemotherapy: MD Radiotherapy: MD	Sample(s): n.301 Method(s) of sample collection: Oral rinse n.104 Saliva test n.197 Microorganisms identification technique: Culture n.301 RT-PCR n.52 Target: MD	Than the healthy control group ↑ Candida: n.MD of OSCC case Than the OPMD control group ↑ Candida: n.MD of OSCC case Type(s) of fungi species detected: MD
Mallika, 2020 Trans Cancer Res Studies: n.11 CCS n.11 No meta-analysis Moderate quality No funding	Sample size: MD Mean age: MD Gender ratio: MD Country: MD Risk factors for OSCC: MD History of OPMD: MD Time to OPMD onset: MD Previous history of malignancies: MD Other comorbidities: MD Other ongoing treatments: MD	Macroscopic features: MD Location: MD Staging: MD Grading: MD Microscopic features: MD First diagnosis: MD Time to onset: MD Chemotherapy: MD Radiotherapy: MD	Sample(s): MD Method(s) of sample collection: Saliva test n.MD Oral swab n.MD Microorganisms identification technique: Culture n.MD VIDAS EBV kit n.MD QIAamp Mini Elute Virus Spin kit Digene HPV genotyping RH test n.MD PCR n.MD Spectrophotometer n.MD Gas chromatography n.MD IHC n.MD Target: MD	Than the healthy control group ↑ Candida: MD n./% of OSCC case Type(s) of fungi species detected: MD
Shen, 2023 Arch Oral Biol Studies: n.5 CCS n.5 No meta-analysis High quality This study was supported by the China-Japan Friendship Hospital Research Project Foundation [grant number 2020-1-QN-2].	Sample size: n.230 of case/n.219 of the healthy control group/n.205 of the OPMD control group Mean age: N/D Gender ratio: 27M/203MD Country: USA n.18 India n.31 Taiwan n.N/D China n.29+N/D Risk factors for OSCC: MD History of OPMD: MD Time to OPMD onset: MD Previous history of malignancies: MD Other comorbidities: MD Other ongoing treatments: MD	Macroscopic features: MD Location: MD Staging: MD Grading: MD Microscopic features: MD First diagnosis: MD Time to onset: MD Chemotherapy: MD Radiotherapy: MD	Sample(s): n.230 Method(s) of sample collection: Saliva test n.152 Oral rinse n.49 Oral swab, plaque swab, and saliva test n.29 Microorganisms identification technique: Modified QIAGEN DNA n.18 Gene Fix Saliva Prep 2 Isolation kit n.31 QIAamp DNA Blood mini kit n.124 QIAamp DNA Mini kit n.21 HiPure tissue and blood DNA kit n.32 Target: 16s rRNA: n.177 V4 region n.124 V3-V4 region n.103 ITS1 n.32	Than the healthy and the OPMD control group ↑ Candida: n.29 of OSCC case ↑ Aspergillus: MD n./% of OSCC case ↑ Acremonium: MD n./% of OSCC case ↓ Morchella: MD n./% of OSCC case

Abbreviations: number, “n.”; years old, “y.o.”; percentage, “%”; greater than, “>”; less than, “<”; missing data, “MD”; not defined, “N/D”; not statistically significant, “NSS”; not significant, “NS”; *p*-value, “*p*”; male, “M”; female, “F”; case-control study, “CCS”; cohort study, “CS”; cross-sectional study, “CSS”; cross-sectional cohort study, “CSCS”; oral squamous cell carcinoma, “OSCC”; oral potentially malignant disorder, “OPMD”; United States of America, “USA”; polymerase chain reaction, “PCR”; real time polymerase chain reaction, “RT-PCR”; quantitative polymerase chain reaction, “qPCR”; immunohistochemistry, “IHC”; Ribosomal Ribonucleic Acid, “rRNA”; RiboNucleic Acid, “RNA”; DeoxyriboNucleic Acid, “DNA”; subspecies, “ssp”; Fusobacterium, “F.”; Campylobacter, “C.”; Streptococcus, “S.”; Human Papilloma Virus, “HPV”; Low Risk Human Papilloma Virus, “LR-HPV”; High Risk Human Papilloma Virus, “HR-HPV”; Epstein Barr Virus, “EBV”; increased, “↑”; decreased, “↓”.

## Data Availability

Data are available in the MEDLINE/PubMed, Scopus, and BioMed Central databases.

## Supplementary Materials

The following supporting information can be downloaded at: https://www.mdpi.com/article/10.3390/cancers15235540/s1. File S1: Oral Bacteria, Virus and Fungi in Saliva from Adult Subjects with Oral Squamous Cell Carcinoma. File S2: Oral Bacteria, Virus and Fungi in Saliva from Adult Subjects with Oral Squamous Cell Carcinoma. File S3: Quality assessment of included studies.
