# Supplementary material for: Oral Bacteria, Virus and Fungi in Saliva and Tissue Samples from Adult Subjects with Oral Squamous Cell Carcinoma: An Umbrella Review
Source: Cancers (Basel). 2023 Nov 22;15(23):5540. doi: 10.3390/cancers15235540 (PMC10705713; doi:10.3390/cancers15235540)
Supplement: Supplementary file 1 [file cancers-15-05540-s001.zip › Supplementary file S1.pdf]

Supplementary file 1 (S1) - Oral Bacteria, Virus and Fungi in Saliva from Adult Subjects with Oral Squamous Cell Carcinoma

Table S1. Data extracted and collected from the included studies that had carried out a histopathologic analysis of OSCC-tissue lesions. Studies: first Author, year and journal of publication, reference, number and design of included studies, meta-analysis, assessed quality, funding (if any). Population characteristics: sample size, mean age, gender ratio, country of origin of the sample, risk factor and history of OPMD, history of malignancies, other comorbidities and ongoing treatments. OSCC characteristics: macroscopic features, location, staging and grading, microscopic features, first diagnosis (primary site/metastatic lesion), time to onset, chemotherapy (yes/no), radiotherapy (yes/no). Intervention: number of sample(s), method(s) of sample collection, microorganisms identification technique, target. Outcome(s): type(s) of phylum, genus and species of bacterium, type(s) and genotype of virus, type(s) and species of fungus detected, number or percentage of OSCC positive case.

STUDIES	POPULATION		INTERVENTION	OUTCOME(S)		
	Characteristics	OSCC		Bacteria	Viruses	Fungi
First Author, year<br>*Journal of publication*<br>Reference<br>Studies: n.<br>Type n.<br>Meta-analysis<br>Assessed quality<br>Funding (if any)	Sample size: n. …of case/n. … of control<br>Mean age: ... y.o.; range …-…<br>Gender ratio: M/F<br>Country: (origin of the sampling)<br>Risk factors for OSCC:<br>History of OPMD:<br>Time to OPMD onset:<br>Previous history of malignancies:<br>Other comorbidities:<br>Other ongoing treatments:	Macroscopic features:<br>Location:<br>Staging:<br>Grading:<br>Microscopic features:<br>First Diagnosis (metastatic lesion/primary site):<br>Time to onset:<br>Chemotherapy:<br>Radiotherapy:	Sample(s):<br>Method(s) of sample collection:<br>Microorganisms identification technique:<br>Target:	Type(s) of bacterium: n. …/ % of OSCC case<br>Type(s) of bacterium species detected:	Type(s) of virus: n. …/ % of OSCC case<br>Genotype(s) of virus detected:	Type(s) of fungus: n. …/ % of OSCC case<br>Type(s) of fungi species detected:
Ayuningtyas, 2022<br>*Pathophysiology*<br>Studies: n.5<br>RCSS n.2<br>CSS n.2<br>PS n.1	Sample size: n.136+MD of case/n.92+MD of the healthy control group/n.107+MD of OPMD control group<br>Mean age: MD<br>Gender ratio: MD<br>Country:<br>India n.80<br>Egypt n.31	Macroscopic features: MD<br>Location: MD<br>Staging: MD<br>Grading: MD<br>Microscopic features: MD<br>First Diagnosis: MD<br>Time to onset: MD	Sample(s): n.136+MD<br>Method(s) of sample collection:<br>Biopsy n.61<br>Swab n.50<br>Biopsy and Swab n.25+MD	-	-	Than the healthy control group<br>↑ Candida: n.MD of OSCC case<br>Than the OPMD control group

No Meta-analysis<br><br>Critically Low quality<br><br>No Funding	Argentina n.25<br>Taiwan n.MD<br><br>Risk factors for OSCC: MD<br>History of OPMD: MD<br>Time to OPMD onset: MD<br>Previous history of malignancies: MD<br>Other comorbidities: MD<br>Other ongoing treatments: MD	Chemotherapy: MD<br>Radiotherapy: MD	Microorganisms identification technique:<br>Culture n.136+MD<br>IHC: n.MD<br><br>Target: MD			↑ Candida: n.MD of OSCC case<br><br>Type(s) of fungi species detected: MD
Bronzato D.J., 2020<br><br>*Arch Oral Biol*<br><br>Studies: n. 13<br>CCS n.13<br><br>Meta-analysis<br><br>Moderate quality<br><br>This study was supported by CAPES, FAPESP and CNPq.	Sample size: n.294 of case<br>Mean age: MD<br>Gender ratio: MD<br>Country:<br>USA n.52<br>China n.40<br>India n.40<br>Germany n.33<br>Hungary n.31<br>Wales n.30<br>Sri Lanka n.27<br>Japan n.21<br>Yemen n.N/D<br>Saudi Arabia n.N/D<br><br>Risk factors for OSCC: MD<br>History of OPMD: MD<br>Time to OPMD onset: MD<br>Previous history of malignancies: MD<br>Other comorbidities: MD<br>Other ongoing treatments: MD	Macroscopic features: MD<br>Location:<br>N/D oral cavity n.161;<br>gingiva n. MD;<br>tongue n. MD;<br>cheek n. MD;<br>oral floor n. MD;<br>mandible n. MD;<br>buccal mucosa n. MD.<br><br>Staging: MD<br>Grading: MD<br>Microscopic features: MD<br><br>First Diagnosis: MD<br>Time to onset: MD<br>Chemotherapy: MD<br>Radiotherapy: MD	Sample(s): n.294<br><br>Method(s) of sample collection:<br>Biopsy n.125<br>Swab n.146<br>Biopsy and Swab n.20<br>Sterile paper point n.3<br><br>Microorganisms identification technique:<br>Culture n.142<br>PCR n.152<br><br>Target:<br>16S rDNA n.42<br>16S rRNA n.107<br>MD n.145	Than the healthy control group<br><br>Fusobacteria:<br>↑ Fusobacterium: n.132 of OSCC case<br>Type(s) of Fusobacterium species detected:<br>F. nucleatum;<br>F. naviforme;<br>F. periodonticum;<br>F. canifelinum;<br>F. oral taxon (A71, 203, 370);<br>F. necrophorum;<br>F. gonidiaformans;<br>F. simiae.<br>Type(s) of Fusobacterium nucleatum subspecies:<br>F. nucleatum ssp. nucleatum;<br>F. nucleatum ssp. vicentii;<br>F. nucleatum ssp. polymorphum;<br>F. nucleatum ssp. animalis.	–	–
Chaitanya N.C., 2016<br><br>*J Cancer Res Ther*<br><br>Studies: n. 11	Sample size: n. N/D of case/n. 3212 of the healthy control group<br>Mean age: MD<br>Gender ratio: MD<br>Country: MD	Macroscopic features: MD<br>Location:<br>tongue n. MD<br>buccal mucosa n. MD<br>dentoalveolar complex n. MD	Sample(s): n. N/D<br><br>Method(s) of sample collection:<br>Serum analysis n. MD<br>Biopsy n. MD	–	Than the healthy control group<br><br>↑ HPV: n.N/D of OSCC case	–

CCS n.11<br><br>Meta-analysis<br><br>Critically Low quality<br><br>No Funding	Risk factors for OSCC: MD<br>History of OPMD: MD<br>Time to OPMD onset: MD<br>Previous history of malignancies: MD<br>Other comorbidities: MD<br>Other ongoing treatments: MD	oral floor n. MD<br><br>Staging: MD<br>Grading: MD<br>Microscopic features: MD<br><br>First Diagnosis: MD<br>Time to onset: MD<br>Chemotherapy: MD<br>Radiotherapy: MD	Brush n. MD<br><br>Microorganisms identification technique: N/D<br><br>Target:<br>HPV DNA n.MD		20.34% OSCC of the tongue<br>8.70% OSCC of the oral floor<br>8.00% OSCC of the dentoalveolar complex<br>5.00% OSCC of the buccal mucosa<br><br>Genotype(s) of HPV detected: MD	
Christianto S., 2022<br><br>*Laryngoscope*<br><br>Studies: n. 22<br>CS n.22<br><br>Meta-analysis<br><br>Critically Low quality<br><br>No Funding	Sample size: n.3065 of case<br>Mean age: MD<br>Gender ratio: 2155M/720F/190 N/D<br>Country: MD<br><br>Risk factors for OSCC: MD<br>History of OPMD: MD<br>Time to OPMD onset: MD<br>Previous history of malignancies: MD<br>Other comorbidities: MD<br>Other ongoing treatments: MD	Macroscopic features: MD<br>Location: MD<br><br>Staging:<br>Stage I (n.134)<br>Stage II (n.173)<br>Stage III (n.172)<br>Stage IV (n.551)<br>Stage MD (n.2303)<br>Grading: MD<br>Microscopic features: MD<br><br>First Diagnosis: MD<br>Time to onset: MD<br>Chemotherapy: MD<br>Radiotherapy: MD	Sample(s): n.3065<br><br>Method(s) of sample collection: MD<br><br>Microorganisms identification technique:<br>PCR n.1800<br>IHC n.779<br>ISH n.264<br>PCR/IHC/ISH n.N/D<br><br>Target: MD	–	↑ HPV: n.672 (prevalence 21.92) of OSCC case<br><br>Genotype(s) of HPV detected:<br>HPV-16: n.82<br>HPV-6, -11, -16, -18, -26, -31, -32, -33, -34, -35, -37, -39, -40, -42, -43, -44, -45, -51, -52, -53, 54, -55, -56, -58, -59, -61, -62, -66, -67, -68, -69, -70, -71, -72, -74, -81, -82: n.N/D	–
de Carvalho Melo, 2021<br><br>*Braz J Otorhinolaryngol*<br><br>Studies: n.5<br>CCS n.5<br><br>No Meta-analysis<br><br>Low quality	Sample size: n.383 of case<br>Mean age: N/D y.o.; range 19-92 y.o.<br>Gender ratio: 218M/165F<br>Country:<br>USA n.113<br>Greece n.53<br>Chile n.80<br>China n.137<br><br>Risk factors for OSCC:<br>Tobacco n.2 of OSCC HPV+	Macroscopic features: MD<br>Location:<br>tongue n.9;<br>dentoalveolar complex n.1;<br>MD n.373.<br><br>Staging: MD<br>Grading: MD<br>Microscopic features: MD<br><br>First Diagnosis: MD<br>Time to onset: MD	Sample(s): n.383<br><br>Method(s) of sample collection:<br>Biopsy n.225<br>MD n.158<br><br>Microorganisms identification technique: N/D<br><br>Target:	–	HPV: n.16 of OSCC case<br><br>Genotype(s) of HPV detected:<br>HPV-16: n.14<br>HPV-18: n.2<br>NS	–

No Funding	Alcohol n.2 of OSCC HPV+<br>History of OPMD: MD<br>Time to OPMD onset: MD<br>Previous history of malignancies: MD<br>Other comorbidities: MD<br>Other ongoing treatments: MD	Chemotherapy: MD<br>Radiotherapy: MD	E6 mRNA n.MD<br>E7 mRNA n.MD			
de Lima, 2014<br><br>*J Bras Patol Med Lab*<br><br>Studies: n.37<br>MD n.37<br><br>No Meta-analysis<br><br>Critically Low<br><br>No Funding	Sample size: n.N/D of case/n.N/D of the healthy control group<br>Mean age: MD<br>Gender ratio: MD<br>Country:<br>Japan n.N/D<br>Taiwan n.N/D<br>India n.N/D<br>China n.N/D<br>South Africa n.N/D<br>Sudan n.N/D<br>Finland n.N/D<br>Italy n.N/D<br>Spain n.N/D<br>Germany n.N/D<br>Hungary n.N/D<br>Czech Republic n.N/D<br>The Netherlands n.N/D<br>Serbia n.N/D<br>USA n.N/D<br>Venezuela n.N/D<br>Brazil n.N/D<br>Argentina n.N/D<br><br>Risk factors for OSCC:<br>Tobacco and alcohol n.36<br>History of OPMD: MD<br>Time to OPMD onset: MD<br>Previous history of malignancies: MD<br>Other comorbidities: MD	Macroscopic features: MD<br>Location:<br>tongue n.12<br><br>Staging:<br>In situ OSCC (n.7)<br>Stage II (n.12)<br>Grading: MD<br>Microscopic features: MD<br><br>First Diagnosis: MD<br>Time to onset: MD<br>Chemotherapy: MD<br>Radiotherapy: MD	Sample(s): n.N/D<br><br>Method(s) of sample collection: MD<br><br>Microorganisms identification technique:<br>PCR: n.940<br>In situ PCR: n.220<br>Nested PCR: n.290<br>RT-PCR: n.59<br>PCR dot blot: n.198<br>Differential PCR: n.60<br>IHC: n.635<br>ISH: n.864<br>Radioactive ISH: n.117<br>DNA sequencing: n.64<br>Single strand conformation polymorphism: n.60<br><br>Target:<br>HPV DNA: n.640<br>p16: n.193<br>p53: n.496<br>p21: n.33<br>pRb: n.112<br>BCL-2: n.43<br>hTERT: n.35<br>C-MYC: n.60<br>EBV genome: n.601	-	↑ HPV genome: n.555 of OSCC case<br><br>Genotype(s) of HPV detected:<br>HPV-6, -11, -16, -18, -31, -33: n.N/D<br><br>↑ EBV: n.236 of OSCC case	-

	Other ongoing treatments: MD					
de Lima, 2019<br><br>*Crit Rev Oncog*<br><br>Studies: n.52<br>MD n.52<br><br>Meta-analysis<br><br>Critically Low quality<br><br>No Funding	Sample size: n.2665 of case/n. N/D of the healthy control group/n.N/D of the OPMD control group<br>Mean age: MD<br>Gender ratio: MD<br>Country: N/D<br><br>Risk factors for OSCC: MD<br>History of OPMD: MD<br>Time to OPMD onset: MD<br>Previous history of malignancies: MD<br>Other comorbidities: MD<br>Other ongoing treatments: MD	Macroscopic features: MD<br>Location: MD<br><br>Staging: MD<br>Grading: MD<br>Microscopic features: MD<br><br>First Diagnosis: MD<br>Time to onset: MD<br>Chemotherapy: MD<br>Radiotherapy: MD	Sample(s): n.2665<br><br>Method(s) of sample collection:<br>Biopsy n.2530<br>Brush n.135<br><br>Microorganisms identification technique:<br>PCR: n.1016<br>In situ PCR: n.102<br>PCR Southern blot: n.411<br>RT-PCR Southern blot: n.40<br>Real time qPCR: n. 158<br>qPCR: n.128<br>Real time RT-PCR: n.35<br>Nested PCR: n.708<br>PCR ELISA: n.79<br>IF: n.85<br>IHC: n.721<br>ISH: n.179<br>RNA ISH: n.668<br>Radioactive RNA ISH: n.4<br>DNA ISH: n.142<br>RNA microarray: n.151<br>NASBA: n.9<br><br>Target:<br>EBV: n.471<br>EBNA1: n.458<br>EBNA2: n.444<br>LMP gene: n.33<br>LMP1: n.716<br>LMP2: n.4<br>EBER: n.535<br>EBER1: n.295<br>BZLF1: n.240<br>BNLF1: n.127	-	EBV: n.1207 (prevalence 45.29%) of OSCC case<br>p = 0.000<br><br>Genotype(s) of EBV detected: MD	-

			BNRF1: n.16<br>BNFR1 n.60<br>BMLF1: n.16<br>BHRF1: n.9<br>BARF0: n.10<br>IR-1 region: n.36<br>IR-3 region: n.1<br>71 EBV fragments: n.57<br>BamHI W: n.974<br>BamHI L: n.103<br>MD n.31			
Gopinath, 2019<br><br>*Crit Rev Oncol Hematol*<br><br>Studies: n.7<br>CCS n.7<br><br>No Meta-analysis<br><br>Low quality<br><br>No Funding	Sample size: n.199 of case/n.201 of the healthy control group<br>Mean age: 60,85 y.o.; range MD<br>Gender ratio: MD<br>Country:<br>United Kingdom n.10<br>USA n.64<br>Yemen n.20<br>Sri Lanka n.25<br>China n.80<br><br>Risk factors for OSCC: N/D<br>History of OPMD: MD<br>Time to OPMD onset: MD<br>Previous history of malignancies: MD<br>Other comorbidities: MD<br>Other ongoing treatments: MD	Macroscopic features: MD<br>Location:<br>tongue n.39<br>MD n.160<br><br>Staging: MD<br>Grading: MD<br>Microscopic features: MD<br><br>First Diagnosis: MD<br>Time to onset: MD<br>Chemotherapy: MD<br>Radiotherapy: MD	Sample(s): n.199<br><br>Method(s) of sample collection:<br>Biopsy n.104<br>Swab n.95<br><br>Microorganisms identification technique:<br>DNA isolation kit n.30<br>Incubation in Proteinase K and DNA purification kit n.10<br>Incubation in Proteinase K and DNA easy kit n.15<br>Gentra Puregene Tissue kit n.25<br>QIAamp DNA Stool mini kit n.39<br>QIAamp DNA Mini kit n.80<br><br>Target:<br>16s rRNA: n.10<br>V1-V3 region: n.45<br>V4-V5 region: n.90<br>V4 region: n.54	Than the healthy control group<br><br>↑ Fusobacteria:<br>↑ Fusobacterium<br>Type(s) of Fusobacterium species detected:<br>F. nucleatum ssp. polymorphum<br>F. naviforme<br><br>↑ Spirochaetes<br><br>↑ Proteobacteria:<br>↑ Campylobacter<br>Type(s) of Campylobacter species detected:<br>C. Oral taxon 44<br>↑ Pseudomonas<br>Type(s) of Pseudomonas species detected:<br>↑ Pseudomonas aeruginosa<br>↑ Ralstonia<br>Type(s) of Ralstonia species detected:<br>Ralstonia insidosa<br><br>↑ Bacteroidetes:	–	-

				↑ Capnocytophaga<br><br>↓ Actinobacteria:<br>↑ Corynebacterium<br>↑ Atopobium<br>↑ Actinomyces<br>↑ Rothia<br>↑ Micrococcus<br>↑ Clavibacter michiganensis<br>Type(s) of Clavibacter michiganensis species detected:<br>Clavibacter michiganensis tellarius<br><br>↓/↑ Firmicutes:<br>↑ Enterococcus<br>↑ Gemella<br>Type(s) of Gemella species detected:<br>Gemella haemolysans<br>Gemella morbillorum<br>↑ Streptococcus<br>Type(s) of Streptococcus species detected:<br>S. salivarius<br>S. oral taxon 058<br>S. gordonii<br>S. parasanguinis<br>↑ Johnsonella<br>Type(s) of Johnsonella species detected:<br>Johnsonella ignava<br>↑ Peptostreptococcus:<br>Type(s) of Peptostreptococcus species detected:<br>Pepetosptreptoccus stomatis		
Guo, 2018	Sample size: n.1275 of case<br>Mean age: MD	Macroscopic features: MD<br>Location: MD	Sample(s): n.1275	-	↑ HPV: n.190 (prevalence 14.9%) of OSCC case	-

Front Oncol<br><br>Studies: n.11<br>MD n.11<br><br>Meta-analysis<br><br>Critically Low<br><br>This study was supported by the National Natural Science Foundation of China (No.81703298).	Gender ratio: MD<br>Country:<br>China n.1275<br><br>Risk factors for OSCC: MD<br>History of OPMD: MD<br>Time to OPMD onset: MD<br>Previous history of malignancies: MD<br>Other comorbidities: MD<br>Other ongoing treatments: MD	Staging: MD<br>Grading: MD<br>Microscopic features: MD<br><br>First Diagnosis: MD<br>Time to onset: MD<br>Chemotherapy: MD<br>Radiotherapy: MD	Method(s) of sample collection:<br>Biopsy n.1275<br><br>Microorganisms identification technique:<br>PCR: n.921+N/D<br>ISH: n.N/D<br><br>Target:<br>HPV-16: n.1275		$p < 0.001$<br><br>Genotype(s) of HPV detected:<br>HPV-16: n.190	
Gupta, 2020<br><br>*Clin Oral Investig*<br><br>Studies: n.7<br>CSS n.7<br><br>No Meta-analysis<br><br>Low quality<br><br>No Funding	Sample size: n.513 of case/n.354 of the healthy control group<br>Mean age: MD<br>Gender ratio: 39M/44F/430MD<br>Country:<br>Japan n.58<br>Sri Lanka n.53<br>India n.60<br>Iran n.83<br>Germany n.191<br>China n.68<br><br>Risk factors for OSCC:<br>Betel chewers n.44<br>History of OPMD: MD<br>Time to OPMD onset: MD<br>Previous history of malignancies: MD<br>Other comorbidities: MD<br>Other ongoing treatments: MD	Macroscopic features: MD<br>Location: MD<br><br>Staging: MD<br>Grading: MD<br>Microscopic features: MD<br><br>First Diagnosis: MD<br>Time to onset: MD<br>Chemotherapy: MD<br>Radiotherapy: MD	Sample(s): n.513<br><br>Method(s) of sample collection:<br>Swab n.58<br>Biopsy n.455<br><br>Microorganisms identification technique:<br>PCR: n.128<br>RT-PCR: n.58<br>Culture: n.131<br>Giemsa: n.68<br>IHC: n.274<br>ELISA: n.121<br><br>Target: MD	↑ Than the healthy control group (prevalence 31.92%)<br><br>Proteobacteria:<br>Helicobacter<br>Type(s) of Helicobacter species detected:<br>↑ Helicobacter pylori: n.165 (prevalence 32.16%) of OSCC case	-	-
Haghshenas, 2022	Sample size: n.69 of case/n.57 of the healthy control group<br>Mean age: MD	Macroscopic features: MD<br>Location: MD	Sample(s): n.69	-	Than the healthy control group (prevalence 0.00%)	-

Iran J Public Health<br><br>Studies: n.2<br>CCS n.2<br><br>Meta-analysis<br><br>Critically Low<br><br>This study was supported by the Mazandaran University of Medical Sciences (IR.MAZUMS. REC.1399.8657).	Gender ratio: MD<br>Country: MD<br><br>Risk factors for OSCC: MD<br>History of OPMD: MD<br>Time to OPMD onset: MD<br>Previous history of malignancies: MD<br>Other comorbidities: MD<br>Other ongoing treatments: MD	Staging: MD<br>Grading: MD<br>Microscopic features: MD<br><br>First Diagnosis: MD<br>Time to onset: MD<br>Chemotherapy: MD<br>Radiotherapy: MD	Method(s) of sample collection:<br>Biopsy n.69<br><br>Microorganisms identification technique: N/D<br><br>Target: MD		↑ HPV: n.20 (prevalence 28.99%) of OSCC case<br><br>Genotype(s) of HPV detected:<br>HPV-16: n.5<br>HPV-18: n.2<br>MD n.13	
Hobbs, 2006<br><br>*Clin Otolaryngol*<br><br>Studies: n.15<br>MD n.15<br><br>Meta-analysis<br><br>Critically Low<br><br>No Funding	Sample size: n.1873 of case/n.2437 of the healthy control group<br>Mean age: MD<br>Gender ratio: MD<br>Country: MD<br><br>Risk factors for OSCC: MD<br>History of OPMD: MD<br>Time to OPMD onset: MD<br>Previous history of malignancies: MD<br>Other comorbidities: MD<br>Other ongoing treatments: MD	Macroscopic features: MD<br>Location:<br>N/D oral cavity n.1656<br>Tonsil n.217<br><br>Staging: MD<br>Grading: MD<br>Microscopic features: MD<br><br>First Diagnosis: MD<br>Time to onset: MD<br>Chemotherapy: MD<br>Radiotherapy: MD	Sample(s): n.1873<br><br>Method(s) of sample collection:<br>Biopsy n.322<br>Serum anaylsis n.1551<br><br>Microorganisms identification technique: N/D<br><br>Target: N/D	-	↑ HPV: n.314 (prevalence 16.76%) of OSCC case<br><br>n.219 (13.22%): N/D oral cavity<br>n.95 (43.78%): tonsil<br><br>Genotype(s) of HPV detected: N/D	-
Huybrechts, 2020<br><br>*Cancer Epidemiol Biomarkers Prev*<br><br>Studies: n.13<br>CS n.2<br>CCS n.11	Sample size: n.724 of case/n.1188 of the healthy control group<br>Mean age: MD<br>Gender ratio: MD<br>Country: MD<br><br>Risk factors for OSCC: MD	Macroscopic features: MD<br>Location: MD<br><br>Staging: MD<br>Grading: MD<br>Microscopic features: MD<br><br>First Diagnosis: MD	Sample(s): n.724<br><br>Method(s) of sample collection:<br>Biopsy n.724	↑ Bacteroidetes:<br>↑ Capnocytophaga<br><br>↑ Fusobacteria:<br>↑ Fusobacterium<br><br>Firmicutes:<br>↑ Dialister	-	-

No Meta-analysis<br><br>Low quality<br><br>This study was supported by the Intramural Research Program of the National Cancer Institute at the National Institutes of Health and by the Research Foundation-Flanders 12h1519N.	History of OPMD: MD<br>Time to OPMD onset: MD<br>Previous history of malignancies: MD<br>Other comorbidities: MD<br>Other ongoing treatments: MD	Time to onset: MD<br>Chemotherapy: MD<br>Radiotherapy: MD	Microorganisms identification technique: N/D<br><br>Target: MD	↑ Peptostreptococcus<br>↑ Parvimonas		
Kreimer, 2005<br><br>*Cancer Epidemiol Biomarkers Prev*<br><br>Studies: n.38<br>MD n.38<br><br>Meta-analysis<br><br>Critically Low quality<br><br>No Funding	Sample size: n.2642 of case<br>Mean age: MD<br>Gender ratio: MD<br>Country:<br>United Kingdom n.39<br>Germany n.53<br>Switzerland n.15<br>Italy n.38+N/D<br>Slovenia n.55<br>The Netherlands n.105<br>France n.12<br>Spain n.2+N/D<br>Canada n.53<br>USA n.832<br>Finland n.28+N/D<br>Norway n.N/D<br>Sweden n.N/D<br>North Ireland n.N/D<br>Poland n.N/D<br>India n.473<br>Taiwan n.103	Macroscopic features: MD<br>Location: MD<br><br>Staging: MD<br>Grading: MD<br>Microscopic features: MD<br>First Diagnosis: MD<br>Time to onset: MD<br>Chemotherapy: MD<br>Radiotherapy: MD	Sample(s): n.2642<br><br>Method(s) of sample collection:<br>Biopsy n.2642<br><br>Microorganisms identification technique:<br>PCR n.2642<br><br>Target: MD	-	HPV: n.796 (prevalence 23.5%) of OSCC case<br>HPV prevalence in<br>- Europe: 16.0%<br>- North America: 16.1%<br>- Asia: 33.0%<br>- Other: 18.1%<br><br>↓ Than the oropharyngeal SCCs group (prevalence 35.6%) and the laryngeal SCCs group (prevalence 24.0%)<br><br>Genotype(s) of HPV detected:<br>HPV-6: n.59 (7.41%)<br>HPV-11: n.31 (3.89%)<br>HPV-16: n.423 (53.34%)<br>HPV-18: n.212 (26.73%)	-

	Japan n.306<br>China n.85<br>Korea n.76<br>Venezuela n.50<br>Cuba n.N/D<br>Australia n.N/D<br>Sudan n.N/D<br><br>Risk factors for OSCC: MD<br>History of OPMD: MD<br>Time to OPMD onset: MD<br>Previous history of malignancies: MD<br>Other comorbidities: MD<br>Other ongoing treatments: MD				HPV-16 and -18: n.44 (5.55%)<br>HPV-31: n.3 (0.38%)<br>HPV-32: n.1 (0.13%)<br>HPV-33: n.14 (1.76%)<br>HPV-35: n.1 (0.13%)<br>HPV-39: n.0 (0.0%)<br>HPV-44: n.1 (0.13%)<br>HPV-45: n.0 (0.0%)<br>HPV-51: n.0 (0.0%)<br>HPV-52: n.0 (0.0%)<br>HPV-53: n.1 (0.13%)<br>HPV-56: n.2 (0.25%)<br>HPV-57: n.1 (0.13%)<br>HPV-58: n.1 (0.13%)<br>HPV-59: n.0 (0.0%)<br>HPV-68: n.1 (0.13%)<br>HPV-73: n.0 (0.0%)<br>HPV-81: n.1 (0.13%)<br>HPV-82: n.0 (0.0%)	
Mallika, 2020<br><br>*Trans Cancer Res*<br><br>Studies: n.8<br>CCS n.8<br><br>No Meta-analysis<br><br>Moderate quality<br><br>No Funding	Sample size: MD<br>Mean age: MD<br>Gender ratio: MD<br>Country: MD<br><br>Risk factors for OSCC: MD<br>History of OPMD: MD<br>Time to OPMD onset: MD<br>Previous history of malignancies: MD<br>Other comorbidities: MD<br>Other ongoing treatments: MD	Macroscopic features: MD<br>Location: MD<br><br>Staging: MD<br>Grading: MD<br>Microscopic features: MD<br><br>First Diagnosis: MD<br>Time to onset: MD<br>Chemotherapy: yes (one study)<br>Radiotherapy: yes (two studies)	Sample(s): MD<br><br>Method(s) of sample collection:<br>Biopsy n.MD<br>Swab n.MD<br>Brush n.MD<br><br>Microorganisms identification technique:<br>Culture n.MD<br>IHC n.MD<br>ISH n.MD<br>PCR n.MD<br><br>Target: MD	Proteobacteria: Helicobacter<br>Type(s) of Helicobacter species detected:<br>↑ Helicobacter pylori	↑ HPV: MD n./% of OSCC case<br><br>Genotype(s) of HPV detected:<br>HPV-16 n.MD<br>HPV-18 n.MD<br><br>HHV-6: NS<br><br>EBV: NS	↑ Oral yeast: MD n./% of OSCC case<br><br>Candida: MD n./% of OSCC case (two studies of OSCC patients undergoing radiotherapy; one study of OSCC patients undergoing chemotherapy)<br><br>Type(s) of fungi species detected: MD

Miller, 2001<br><br>*Oral Surg Oral Med Oral Pathol Oral Radiol Endod*<br><br>Studies: n.80<br>MD n.80<br><br>Meta-analysis<br><br>Critically Low quality<br><br>No Funding	Sample size: n.N/D of case<br>Mean age: MD<br>Gender ratio: MD<br>Country: MD<br><br>Risk factors for OSCC: MD<br>History of OPMD: MD<br>Time to OPMD onset: MD<br>Previous history of malignancies: MD<br>Other comorbidities: MD<br>Other ongoing treatments: MD	Macroscopic features: MD<br>Location: MD<br><br>Staging: MD<br>Grading: MD<br>Microscopic features: MD<br><br>First Diagnosis: MD<br>Time to onset: MD<br>Chemotherapy: MD<br>Radiotherapy: MD	Sample(s): n.N/D<br><br>Method(s) of sample collection:<br>Biopsy n.N/D<br><br>Microorganisms identification technique:<br>N/D ISH/IP/IF n.608<br>N/D Southern blot/dot blot/filter blot hybridization n.321<br>PCR n.1154<br><br>Target:<br>HPV DNA: n.2083	-	↑ HPV: n.627 (prevalence 46.5%) of OSCC case<br>$p < 0.001$<br><br>Genotype(s) of HPV detected:<br>HPV-2: n.3<br>HPV-3: n.1<br>HPV-4: n.2<br>HPV-6: n.52<br>HPV-10: n.16<br>HPV-11: n.37<br>HPV-13: n.1<br>HPV-16: n.285<br>HPV-18: n.115<br>HPV-31: n.3<br>HPV-33: n.9<br>HPV-57: n.1<br>HPV-6 and -11: n.27<br>HPV-16 and -18: n.49<br>HPV-6, and 11 and 16 and 18: n.1<br>HPV-6 and -16 and -18: n.3<br>HPV-31 and -33 and -35: n.6<br>N/D: 16<br><br>LR-HPV: $p < 0.001$<br>HR-HPV: $P < 0.001$ (prevalence 23.71%)	-
Muthusamy, 2023<br><br>*Cureus*<br><br>Studies: n.6<br>CCS n.6<br><br>Meta-analysis	Sample size: n.373 of case/n.326 of healthy control group/n.73 of OPMD group<br>Mean age: MD<br>Gender ratio: MD<br>Country: MD<br><br>Risk factors for OSCC: MD	Macroscopic features: MD<br>Location: MD<br><br>Staging: MD<br>Grading: MD<br>Microscopic features: MD<br><br>First Diagnosis: MD	Sample(s): n.373<br><br>Method(s) of sample collection:<br>Blood analysis n.50<br>Biopsy n.211<br>Biofilm sampling n.21<br>Brush n.91	Fusobacteria:<br>↑ Fusobacterium<br>$p = 0.05$<br><br>Firmicutes:<br>↑ Streptococcus<br>p = NSS	↑ EBV: MD n./% of OSCC case<br>$p < 0.0001$<br><br>HSV-1: MD n./% of OSCC case<br>p = NSS	-

Critically Low quality<br><br>This study was supported by Indian Council of Medical Research (ICMR) under Nurturing Clinical Scientist (NCS) scheme HRD/Head-NCS-2019-02.	History of OPMD: MD<br>Time to OPMD onset: MD<br>Previous history of malignancies: MD<br>Other comorbidities: MD<br>Other ongoing treatments: MD	Time to onset: MD<br>Chemotherapy: MD<br>Radiotherapy: MD	Microorganisms identification technique:<br>PAP technique n.50<br>Culture n.21<br>Nested PCR n.120<br>ELISA n.132<br>PCR n.50<br><br>Target:<br>Herpes Select-1 n.132<br>16s RNA n.50	Bacteroidetes:<br>↑ Prevotella<br>p = NSS<br>↑ Porphyromonas<br>p = NSS<br><br>Proteobacteria:<br>↑ Neisseria: p= NSS		
Nandi, 2021<br><br>*Cancer Treat Res Commun*<br><br>Studies: n.19<br>CCS n.19<br><br>No Meta-analysis<br><br>Critically Low quality<br><br>No Funding	Sample size: n.1639 of case/n.206 of the healthy control group<br>Mean age: N/D y.o.; range MD<br>Gender ratio: 955M/377F/307MD<br>Country:<br>India n.1639<br><br>Risk factors for OSCC: MD<br>History of OPMD: MD<br>Time to OPMD onset: MD<br>Previous history of malignancies: MD<br>Other comorbidities: MD<br>Other ongoing treatments: MD	Macroscopic features: MD<br>Location:<br>tongue n.156<br>MD n.1483<br><br>Staging: MD<br>Grading: MD<br>Microscopic features: MD<br><br>First Diagnosis: MD<br>Time to onset: MD<br>Chemotherapy: MD<br>Radiotherapy: MD	Sample(s): n.1639<br><br>Method(s) of sample collection: N/D<br><br>Microorganisms identification technique:<br>PCR/p16-IHC n.216<br>ISH n.31<br>IHC n.30<br>PCR/ISH n.45<br>PCR n.1317<br><br>Target:<br>MD	-	↑ HPV: n.553 of OSCC case<br><br>Genotype(s) of HPV detected: MD	-
Rahman, 2023<br><br>*Mol Oral Microbiol*<br><br>Studies: n.15<br>CCS n.5<br>NRS n.10<br><br>Meta-analysis	Sample size: n.1109 of case/n.211 of the healthy control group/n.150 of the OPMD group<br>Mean age: MD<br>Gender ratio: MD<br>Country:<br>USA n.61<br>Poland n.53	Macroscopic features: MD<br>Location:<br>tonsil n.16<br>oral floor n.30<br>Buccal mucosa n.27<br>tongue n.74<br>palate n.16 (soft n.10/MD n.6)<br>lip n.11	Sample(s): n.1109<br><br>Method(s) of sample collection:<br>Biopsy n.1109<br><br>Microorganisms identification technique:<br>PCR n.968	-	HPV-EBV coinfection: n.95 of OSCC case<br>$p < 0.01$<br><br>HPV: n.31 of OSCC case<br><br>Genotype(s) of HPV detected: MD	-

Critically Low<br><br>No Funding	Sweden n.17<br>Norway n.20<br>United Kingdom n.20<br>Sudan n.20<br>India n.20<br>Sri Lanka n.20<br>Yemen n.18<br><br>Risk factors for OSCC: MD<br>History of OPMD: MD<br>Time to OPMD onset: MD<br>Previous history of malignancies: MD<br>Other comorbidities: MD<br>Other ongoing treatments: MD	MD n.935<br><br>Staging: MD<br>Grading: MD<br>Microscopic features: MD<br><br>First Diagnosis: MD<br>Time to onset: MD<br>Chemotherapy: MD<br>Radiotherapy: MD	ISH n.141<br><br>Target: MD			
Ramos, 2020<br><br>*Oral Maxillofac Surg*<br><br>Studies: n.4<br>CCS n.1<br>MD n.3<br><br>No Meta-analysis<br><br>Critically Low quality<br><br>This study was supported by the National Council for Scientific and Technological Development (CNPq) (Project:211309/ 2013-3) and the Foundation for	Sample size: n.124 of case/n.20 of the healthy control group/n.27 of the control group with oral fibroepithelial polyp<br>Mean age: MD<br>Gender ratio: N/D<br>Country: MD<br><br>Risk factors for OSCC:<br>Tobacco: n.N/D<br>Alcohol: n.N/D<br>History of OPMD: MD<br>Time to OPMD onset: MD<br>Previous history of malignancies: MD<br>Other comorbidities: MD<br>Other ongoing treatments: MD	Macroscopic features: MD<br>Location:<br>tongue n.39<br>MD n.85<br><br>Staging: MD<br>Grading: MD<br>Microscopic features: MD<br><br>First Diagnosis: MD<br>Time to onset: MD<br>Chemotherapy: MD<br>Radiotherapy: MD	Sample(s): n.124<br><br>Method(s) of sample collection:<br>Biopsy n.64<br>Swab n.40<br>Swab and Biopsy: n.20<br><br>Microorganisms identification technique:<br>MiSeq n.85<br>Ion torrent n.39<br><br>Target:<br>V4 region n.39<br>V1-V3 region n.45<br>V4-V5 region n.40	↑ Firmicutes:<br>↑ Dialister<br>↑ Catonella<br>↑ Peptostreptococcus<br>↑ Peptococcus<br>↑ Filifactor<br>↑ Parvimonas<br><br>↓ Bacteroidetes:<br>↑ Capnocytophaga<br><br>↑/↓ Fusobacteria:<br>↑ Fusobacterium<br>Type(s) of Fusobacterium species detected:<br>↑ F. nucleatum ssp. polymorphum<br><br>Proteobacteria:<br>↑ Campylobacter<br>Pseudomonas<br>Type(s) of Pseudomonas species detected:	-	-

Research Financial Support in the State of Rio de Janeiro (FAPERJ) (Project: E26/ 1033.001/2012).				↑ Pseudomonas aeruginosa<br><br>Actinobacteria:<br>↑ Atopobium		
Shaikh, 2015<br><br>*Cancer Epidemiol*<br><br>Studies: n.45<br>CSS n.N/D<br>CCS n.N/D<br><br>Meta-analysis<br><br>Low quality<br><br>This study was supported by the Griffith University of Australia.	Sample size: n.4893 of case<br>Mean age: 56.80 y.o.; range 22-94 y.o.<br>Gender ratio: MD<br>Country:<br>India n.1293<br>Pakistan n.48<br>Bangladesh n.34<br>Sri Lanka n.96<br>Malaysia n.109<br>Thailand n.32<br>China n.183<br>Hong Kong n.31<br>Taiwan n.535<br>South Korea n.167<br>Japan n.843<br>Australia n.1522<br><br>Risk factors for OSCC:<br>Tobacco n.171+N/D<br>Tobacco not smoked n.N/D<br>Alcohol n.N/D<br>History of OPMD: MD<br>Time to OPMD onset: MD<br>Previous history of malignancies: MD<br>Other comorbidities: MD<br>Other ongoing treatments: MD	Macroscopic features: MD<br>Location:<br>buccal mucosa n.433<br>gingiva n.196<br>tongue n.679<br>oral floor n.96<br>palate n.64 (hard n.1/soft n.5/MD n.59)<br>tonsil n.1587<br>lip n.114<br>N/D oral cavity n.2353<br><br>Staging: MD<br>Grading: MD<br>Microscopic features: MD<br><br>First Diagnosis: MD<br>Time to onset: MD<br>Chemotherapy: MD<br>Radiotherapy: MD	Sample(s): n.4893<br><br>Method(s) of sample collection:<br>Biopsy n.4893<br><br>Microorganisms identification technique:<br>PCR n.4209<br>Southern blot PCR n.224<br>Slot blot PCR n.15<br>Nested PCR n.27<br>ISH n.102<br>PCR and ISH: n.244<br>RT-PCR and IHC: n.52<br>In situ PCR and ISH n.20<br><br>Target:<br>HPV genome: n.N/D	-	↑ HPV: n.1627 of OSCC case<br><br>n.146 (33.72%): buccal mucosa<br>n.59 (30.10%): gingiva<br>n.219 (32.25%): tongue<br>n.29 (30.21%): oral floor<br>n.20 (31.25%): palate<br>n.489 (30.81%): tonsil<br>n.19 (16.67%): lip<br>n.646 (27.45%): N/D oral cavity<br><br>Genotype(s) of virus detected:<br>HPV-6, -8, -11, -16, -18, -22, -31, -32, -33, -35, -38, -39, -44, -45, -51, -52, -53, -54, -58, -59, -61, -66, -67, -68, -69, -70, -75, -76: n.N/D	-
She, 2017<br><br>*PLoS One*	Sample size: n.686 of case/n. 412 of the healthy control group<br>Mean age: MD	Macroscopic features: MD<br>Location: MD<br><br>Staging: MD	Sample(s): n.686<br><br>Method(s) of sample collection:	-	↑ EBV: n.332 of OSCC case<br>p = 0.002	-

Studies: n.13<br>CCS n.13<br><br>Meta-analysis<br><br>Moderate quality<br><br>No Funding	Gender ratio: MD<br>Country:<br>South Africa n.138<br>Netherland n.36<br>China n.81<br>India n.103<br>Japan n.186<br>Sweden n.29<br>Egypt n.22<br>Spain n.5<br>Hungary n.65<br>USA n.21<br><br>Risk factors for OSCC: MD<br>History of OPMD: MD<br>Time to OPMD onset: MD<br>Previous history of malignancies: MD<br>Other comorbidities: MD<br>Other ongoing treatments: MD	Grading: MD<br>Microscopic features: MD<br><br>First Diagnosis: MD<br>Time to onset: MD<br>Chemotherapy: MD<br>Radiotherapy: MD	Biopsy n.686<br><br>Microorganisms identification technique:<br>PCR n.562<br>Nested PCR n.34<br>ISH n.36<br>IHC n.33<br>RT-qPCR n.21<br><br>Target:<br>EBV DNA n.218<br>EBV DNA BamHIW n.275<br>EBV DNA EBNA2 n.150<br>EBV RNA EBER1 n.21<br>EBV protein n.22			
Shen, 2023<br><br>*Arch Oral Biol*<br><br>Studies: n.2<br>CCS n.2<br><br>No Meta-analysis<br><br>High quality<br><br>This study was supported by the China-Japan Friendship Hospital Research Project Foundation [grant	Sample size: n.25 of case/n.15 of the healthy control group/n.17 of the OPMD control group<br>Mean age: 56.25 y.o.; range MD<br>Gender ratio: MD<br>Country:<br>USA n.16<br>Malaysia n.9<br><br>Risk factors for OSCC: MD<br>History of OPMD: MD<br>Time to OPMD onset: MD<br>Previous history of malignancies: MD<br>Other comorbidities: MD<br>Other ongoing treatments: MD	Macroscopic features: MD<br>Location: MD<br><br>Staging: MD<br>Grading: MD<br>Microscopic features: MD<br><br>First Diagnosis: MD<br>Time to onset: MD<br>Chemotherapy: MD<br>Radiotherapy: MD	Sample(s): n.25<br><br>Method(s) of sample collection:<br>Swab n.25<br><br>Microorganisms identification technique:<br>Incubation Proteinaise K & DNA easy kit n.16<br>DNA extraction kit n.9<br><br>Target:<br>16s rRNA n.25<br>V4 region n.16<br>RFLP gene n.9	Than the healthy and the OPMD control group<br><br>Fusobacteria:<br>↑ Fusobacterium<br><br>↑ Proteobacteria:<br>↑ Neisseria<br><br>↓ Firmicutes:<br>↑ Gemella<br>↑ Granulicatella<br><br>Actinobacteria:<br>↓ Rothia<br><br>Than the healthy control group	-	-

number 2020-1-QN-2].				↑ Bacteroidetes<br><br>↓ Firmicutes:<br>↓ Streptococcus<br>↓ Veillonella<br><br>↑/↓ Actinobacteria<br><br>Than the OPMD control group<br><br>↓ Bacteroidetes<br><br>↓ Fusobacteria		
Sivakumar, 2020<br><br>*Transl Cancer Res*<br><br>Studies: n.7<br>CCS n.7<br><br>Meta-analysis<br><br>Low quality<br><br>No Funding	Sample size: n.349 of case/n.205 of the healthy control group<br>Mean age: MD<br>Gender ratio: MD<br>Country: MD<br><br>Risk factors for OSCC: MD<br>History of OPMD: MD<br>Time to OPMD onset: MD<br>Previous history of malignancies: MD<br>Other comorbidities: MD<br>Other ongoing treatments: MD	Macroscopic features: MD<br>Location: MD<br><br>Staging: MD<br>Grading: MD<br>Microscopic features: MD<br><br>First Diagnosis: MD<br>Time to onset: MD<br>Chemotherapy: MD<br>Radiotherapy: MD	Sample(s): n.349<br><br>Method(s) of sample collection:<br>Biopsy n.349<br><br>Microorganisms identification technique:<br>IHC n.61<br>ISH n.20<br>PCR n.190<br>N/D n.78<br><br>Target:<br>LMP-1 n.61<br>EBV DNA n.60<br>EBNA-2 n.150<br>N/D n.349	–	Than the healthy control group (prevalence 20.0%)<br><br>↑ EBV: n.161 (prevalence 46.13%) of OSCC case	–
Su Mun, 2021<br><br>*Int J Environ Res Public Health*<br><br>Studies: n.7<br>CCS n.7	Sample size: n.280 of case/n.191 of the healthy control group<br>Mean age: MD<br>Gender ratio: MD<br>Country:<br>Yemen n.20<br>Sri Lanka n.25	Macroscopic features: MD<br>Location: MD<br>Staging: MD<br>Grading: MD<br>Microscopic features: MD<br>First Diagnosis: MD<br>Time to onset: MD	Sample(s): n.280<br><br>Method(s) of sample collection:<br>Biopsy n.280	Than the healthy control group<br><br>Fusobacteria:<br>↑ Fusobacterium<br>Type(s) of Fusobacterium species detected:	-	-

No Meta-analysis<br><br>High quality<br><br>This study was supported by the International Medical University of Malaysia.	China n.135<br>USA n.100<br><br>Risk factors for OSCC:<br>Betel nut chewers n.N/D<br>Tobacco n.N/D<br>Shammah (smokeless tobacco) n.N/D<br>Alcohol n.N/D<br>History of OPMD: MD<br>Time to OPMD onset: MD<br>Previous history of malignancies: MD<br>Other comorbidities: MD<br>Other ongoing treatments: MD	Chemotherapy: MD<br>Radiotherapy: MD	Microorganisms identification technique:<br>DNA isolation kit n.20<br>Gentra Puregene Tissue kit n.25<br>QIAampFast DNA Stool Mini kit n.61<br>TIANamp Swab DNA kit n.50<br>MD n.124<br><br>Target:<br>V1-V3 region n.45<br>V3-V4 region n.135<br>MD n.100	↑ F. oral taxon 204<br>↑ F. parvimonas<br>↑ F. nucleatum<br>↑ F. nucleatum ssp. polymorphum<br>Leptotrichia:<br>Type(s) of Leptotrichia species detected:<br>↓ Leptotrichia oral taxon 225<br><br>Actinobacteria:<br>↑ Mobiluncus<br>↑/↓ Actinomyces<br>↓ Rothia<br>Type(s) of Rothia species detected:<br>↓ Rothia dentocariosa<br>↓ Rothia mucilaginosa<br>↑ Atopobium<br>↓ Propionibacterium<br>Corynebacterium<br>Type(s) of Corynebacterium species detected:<br>↓ Corynebacterium matruchotii<br>↓ Arthrobacter<br>↓ Microbacterium<br><br>Defferibacteraceae:<br>↓ Mucispirillum<br><br>Proteobacteria:<br>↑ Brevundimonas<br>↑ Aeromonas<br>↑ Frateuria<br>↑ Caulobacter<br>↑ Pseudomonas<br>Type(s) of Pseudomonas species detected:

		↑ Pseudomonas aeruginosa<br>Aggregatibacter<br>Type(s) of Aggregatibacter species detected:<br>↑ Aggregatibacter segnis<br>↑ Campylobacter<br>Type(s) of Campylobacter species detected:<br>↑ C. concisius<br>↑ C. rectus<br>↑ Citrobacter<br>Type(s) of Citrobacter species detected:<br>↑ Citrobacter koseri<br>↓ Lautropia<br>Type(s) of Lautropia species detected:<br>↓ Lautropia mirabilis<br>↓ Sphingomonas<br><br>Firmicutes:<br>↓/↑ Streptococcus:<br>Type(s) of Streptococcus species detected:<br>↑ S. dysgalactiae<br>↓ S. parasanguinis<br>↓ S. mitis<br>↓ S. oralis<br>↓ S. sp oral taxon 423<br>↓ S. sp oral taxon 070<br>↓ S. sp oral taxon 431<br>↓ S. agalactiae<br>↓ Staphylococcus<br>Type(s) of Staphylococcus species detected:<br>↓ Staphylococcus epidermidis<br>↑ Peptostreptococcus<br>Type(s) of Peptostreptococcus species detected:	

				↑ Peptostreptococcus stomatis<br>Granulicatella<br>Type(s) of Granulicatella species detected:<br>↓ Granulicatella adicens<br>↓ Granulicatella elegans<br>↓ Paenibacillus<br>↑ Parvimonas<br>Gemella:<br>Type(s) of Gemella species detected:<br>↑ Gemella morbillorum<br><br>Bacteroidetes:<br>↑ Capnocytophaga<br>Type(s) of Capnocytophaga species detected:<br>↑ Capnocytophaga leadbetteri<br>↑ Prevotella<br>Type(s) of Prevotella species detected:<br>↑ Prevotella salivae<br>↑ Prevotella loeschii<br>↑ Prevotella intermedia<br>Porphyromonas<br>Type(s) of Porphyromonas species detected:<br>↑ Porphyromonas cationiae<br>↓ Porphyromonas gingivalis		
Syrjänen, 2011<br><br>*Oral Dis*<br><br>Studies: n.33<br>CCS n.33<br><br>Meta-analysis	Sample size: n.1885 of case/n.2248 of the healthy control group<br>Mean age: MD<br>Gender ratio: MD<br>Country: MD<br><br>Risk factors for OSCC: MD<br>History of OPMD: MD<br>Time to OPMD onset: MD	Macroscopic features: MD<br>Location: MD<br><br>Staging: MD<br>Grading: MD<br>Microscopic features: MD<br><br>First Diagnosis: MD<br>Time to onset: MD<br>Chemotherapy: MD	Sample(s): n.1885<br><br>Method(s) of sample collection:<br>Brush n.697<br>Biopsy n.1043<br>Biopsy and Brush n.145<br><br>Microorganisms identification technique:	-	HPV: n.634 of OSCC case<br>$p < 0.00001$<br><br>Genotype(s) of virus species detected:<br>HPV-16: n.222<br>$p < 0.00001$	-

Critically Low quality<br><br>No Funding	Previous history of malignancies: MD<br>Other comorbidities: MD<br>Other ongoing treatments: MD	Radiotherapy: MD	PCR n.1821<br>ISH n.64<br><br>Target: MD			
Termine, 2008<br><br>*Ann Oncol*<br><br>Studies: n.47<br>MD n.47<br><br>Meta-analysis<br><br>Critically Low quality<br><br>No funding	Sample size: n.3238 of case<br>Mean age: MD<br>Gender ratio: MD<br>Country:<br>Finland n.91<br>USA n.1082<br>Spain n.42<br>Japan n.606<br>Taiwan n.29<br>Brazil n.69<br>Slovenia n.62<br>South Africa n.271<br>The Netherlands n.35<br>Sudan n.88<br>Hungary n.79<br>Korea n.42<br>Venezuela n.116<br>Greece n.100<br>China n.113<br>Sweden n.134<br>India n.213<br>Italy n.66<br><br>Risk factors for OSCC: MD<br>History of OPMD: MD<br>Time to OPMD onset: MD<br>Previous history of malignancies: MD<br>Other comorbidities: MD<br>Other ongoing treatments: MD	Macroscopic features: MD<br>Location: MD<br><br>Staging: MD<br>Grading: MD<br>Microscopic features: MD<br>First Diagnosis: MD<br>Time to onset: MD<br>Chemotherapy: MD<br>Radiotherapy: MD	Sample(s): n.3238<br><br>Method(s) of sample collection:<br>Biopsy n.3238<br><br>Microorganisms identification technique:<br>ISH: n.370<br>PCR: n.2681<br>N/D ISH or PCR: n.187<br><br>Target: MD	-	↑ HPV: n. 1090 of OSCC case<br>p = 0.000<br><br>Genotype(s) of HPV detected:<br>HPV-6, -11, -16, -18, -22, -31, -33, -35, -38, -58, -68, -70: n.MD	-
Yang, 2019<br><br>*Medicine (Baltimore)*	Sample size: n.758 of case<br>Mean age: MD<br>Gender ratio: MD<br>Country: China n.758	Macroscopic features: MD<br>Location: MD<br><br>Staging:<br>Stage I-IV (n.327)	Sample(s): n.758<br><br>Method(s) of sample collection:<br>Biopsy n.758	-	HPV: n.55/7.2% of OSCC case<br><br>Type(s) of HPV detected:<br>HPV-18: n.55	–

Studies: n.6<br>CSS n.6<br><br>Meta-analysis<br><br>Moderate quality<br><br>No Funding	Risk factors for OSCC: MD<br>History of OPMD: MD<br>Time to OPMD onset: MD<br><br>Previous history of malignancies: MD<br>Other comorbidities: MD<br>Other ongoing treatments: MD	Stage III-IV (n.333)<br>Stage N/D (n.98)<br>Grading: MD<br>Microscopic features: MD<br><br>First Diagnosis: MD<br>Time to onset: MD<br>Chemotherapy: MD<br>Radiotherapy: MD	Microorganisms identification technique:<br>PCR n.704<br>ISH n.54<br><br>Target:<br>L1 region of HPV-18 gene n.533		p = 0.011	
Yu, 2023<br><br>*Heliyon*<br><br>Studies: n.2<br>CCS n.2<br><br>Meta-Analysis<br><br>Moderate quality<br><br>This study was supported by Grant from Qingdao Medical Talents Training Program [VYQ2020Y02].	Sample size: n.30 of case/n.26 of the healthy control group<br>Mean age: 52.3 y.o.; range MD<br>Gender ratio: MD<br>Country: MD<br><br>Risk factors for OSCC: MD<br>History of OPMD: MD<br>Time to OPMD onset: MD<br>Previous history of malignancies: MD<br>Other comorbidities: MD<br>Other ongoing treatments: MD	Macroscopic features: MD<br>Location: MD<br><br>Staging: MD<br>Grading: MD<br>Microscopic features: MD<br><br>First Diagnosis: MD<br>Time to onset: MD<br>Chemotherapy: MD<br>Radiotherapy: MD	Sample(s): n.30<br><br>Method(s) of sample collection:<br>Biopsy n.30<br><br>Microorganisms identification technique:<br>N/D next-generation sequencing technology n.30<br><br>Target:<br>V4 region n.10<br>V1-V3 region n.20	Than the healthy control group<br><br>Fusobacteria<br>↑ Fusobacterium:<br>p=0.000<br><br>↑ Proteobacteria:<br>↓ Haemophilus<br>p= 0.000<br><br>↓ Actinobacteria:<br>↓ Rothia<br><br>↓ Firmicutes:<br>↓ Streptococcus<br>p= 0.032<br><br>↓ Bacteroidetes:<br>↑ Prevotella:<br>NSS	–	–
Zhu, 2012<br><br>*PLoS One*<br><br>Studies: n.18<br>CCS n.18<br><br>Meta-analysis	Sample size: n.610 of case/n.259 of healthy control group<br>Mean age: MD y.o.; range MD<br>Gender ratio: MD<br>Country:<br>China n.610<br><br>Risk factors for OSCC: MD<br>History of OPMD: MD	Macroscopic features: MD<br>Location: MD<br><br>Staging: MD<br>Grading: MD<br>Microscopic features: MD<br><br>First Diagnosis: MD<br>Time to onset: MD	Sample(s): n.610<br><br>Method(s) of sample collection: MD<br><br>Microorganisms identification technique:<br>PCR n.544<br>Dot-blot hybridization n.66	–	Than the healthy control group<br><br>↑ HPV: n.354 of OSCC case<br>$p < 0.00001$<br><br>Type(s) of HPV detected:<br>HPV-16: n.169	–

Critically Low quality<br><br>No Funding	Time to OPMD onset: MD<br>Previous history of malignancies: MD<br>Other comorbidities: MD<br>Other ongoing treatments: MD	Chemotherapy: MD<br>Radiotherapy: MD	Target: MD	$p < 0.00001$<br>MD: n.185

Abbreviations: number, "n."; years old, "y.o."; percentage, "%"; greater than, ">"; missing data, "MD"; not defined, "N/D"; not statistically significant, "NSS"; not significant, "NS"; p-value, "p"; male, "M"; female, "F"; case-control study, "CCS"; cohort study, "CS"; cross-sectional study, "CSS"; retrospective cross-sectional study, "RCSS"; prospective study, "PS"; non-randomized study, "NRS"; Oral Squamous Cell Carcinoma, "OSCC"; Oral Potentially Malignant Disorder, "OPMD"; United States of America, "USA"; polymerase chain reaction, "PCR"; real time polymerase chain reaction, "RT-PCR"; quantitative polymerase chain reaction, "qPCR"; immunohistochemistry, "IHC"; in situ hybridization, "ISH"; enzyme-linked immunosorbent assay, "ELISA"; immunofluorescence, "IF"; immunoperoxidase, "IP"; nucleic acid sequence based amplification, "NASBA"; Papanicolau technique, "PAP technique"; Messenger Ribonucleic Acid, "mRNA"; Ribosomal Ribonucleic Acid, "rRNA"; RiboNucleic Acid, "RNA"; DeoxyriboNucleic Acid, "DNA"; Ribosomal DeoxyriboNucleic Acid, "rDNA"; subspecies, "ssp."; Fusobacterium, "F."; Campylobacter, "C."; Streptococcus, "S."; restriction fragment length polymorphism, "RFLP"; Human Papilloma Virus, "HPV"; Low Risk Human Papilloma Virus, "LR-HPV"; High Risk Human Papilloma Virus, "HR-HPV"; Herpes Simplex Virus, "HSV"; Epstein Barr Virus, "EBV"; Epstein Barr Nuclear Antigen, "EBNA"; Epstein Barr Encoding Region, "EBER"; Latent Membrane Protein, "LMP"; human telomerase reverse transcriptase, "hTERT"; B-cell lymphoma 2, "BCL-2"; Bahm HI N fragment rightward open reading frame, "BNRF"; Bahm HI A fragment rightward open reading frame, "BARF"; Bahm HI H fragment rightward open reading frame, "BHRF"; Bahm HI N fragment leftward open reading frame, "BZLF"; BahmHI M fragment leftward open reading frame, "BMLF"; BahmHI Z fragment leftward open reading frame, "BZLF"; positive, "+"; increased, "↑"; decreased, "↓"; not investigated, "−".
