# Supplementary material for: Oral Bacteria, Virus and Fungi in Saliva and Tissue Samples from Adult Subjects with Oral Squamous Cell Carcinoma: An Umbrella Review
Source: Cancers (Basel). 2023 Nov 22;15(23):5540. doi: 10.3390/cancers15235540 (PMC10705713; doi:10.3390/cancers15235540)
Supplement: Supplementary file 1 [file cancers-15-05540-s001.zip › Supplementary file S2.pdf]

Supplementary file 2 (S2) - Oral Bacteria, Virus and Fungi in Saliva from Adult Subjects with Oral Squamous Cell Carcinoma

Table S2. Data extracted and collected from the included studies that had carried out saliva testing in OSCC adult subjects. Studies: first Author, year and journal of publication, reference, number and design of included studies, meta-analysis, assessed quality, funding (if any). Population characteristics: sample size, mean age, gender ratio, country of origin of the sample, risk factor and history of OPMD, history of malignancies, other comorbidities and ongoing treatments. OSCC characteristics: macroscopic features, location, staging and grading, microscopic features, first diagnosis (primary site/metastatic lesion), time to onset, chemotherapy (yes/no), radiotherapy (yes/no). Intervention: number of sample(s), method(s) of sample collection, microorganisms identification technique, target. Outcome(s): type(s) of phylum, genus and species of bacterium, type(s) and genotype of virus, type(s) and species of fungus detected, number or percentage of OSCC positive case.

STUDIES	POPULATION		INTERVENTION	OUTCOME(S)		
	Characteristics	OSCC		Bacteria	Viruses	Fungi
Ayuningtyas, 2022<br><br>*Pathophysiology*<br><br>Studies: n.4<br>CCS n.1<br>CSS n.3<br><br>No Meta-analysis<br><br>Critically Low quality<br><br>No Funding	Sample size: n.301 of case/n.408 of the healthy control group/n.200 of OPMD control group/n.6 of other malignancy control group<br>Mean age: MD<br>Gender ratio: MD<br>Country:<br>India n.97<br>Australia n.104<br>Finland n.100<br><br>Risk factors for OSCC: MD<br>History of OPMD: MD<br>Time to OPMD onset: MD<br>Previous history of malignancies: MD<br>Other comorbidities: MD<br>Other ongoing treatments: MD	Macroscopic features: MD<br>Location: MD<br><br>Staging: MD<br>Grading: MD<br>Microscopic features: MD<br><br>First Diagnosis: MD<br>Time to onset: MD<br>Chemotherapy: MD<br>Radiotherapy: MD	Sample(s): n.301<br><br>Method(s) of sample collection:<br>Oral rinse n.104<br>Saliva test n.197<br><br>Microorganisms identification technique:<br>Culture n.301<br>RT-PCR n.52<br><br>Target: MD	-	-	Than the healthy control group<br><br>↑ Candida: n.MD of OSCC case<br><br>Than the OPMD control group<br><br>↑ Candida: n.MD of OSCC case<br><br>Type(s) of fungi species detected: MD
de Lima, 2019<br><br>*Crit Rev Oncog*<br><br>Studies: n.1<br>MD n.1	Sample size: n.12 of case/n.47 of the healthy control group/n.12 of the OPMD control group<br>Mean age: MD<br>Gender ratio: MD	Macroscopic features: MD<br>Location: MD<br><br>Staging: MD<br>Grading: MD	Sample(s): n.12<br><br>Method(s) of sample collection:<br>Saliva test n.12	-	EBV: n.7 (prevalence 58.3%) of OSCC case<br><br>↑ Than the healthy control group (prevalence 40.4%)	-

Meta-analysis<br><br>Critically Low quality<br><br>No Funding	Country: MD<br><br>Risk factors for OSCC: MD<br>History of OPMD: MD<br>Time to OPMD onset: MD<br>Previous history of malignancies: MD<br>Other comorbidities: MD<br>Other ongoing treatments: MD	Microscopic features: MD<br><br>First Diagnosis: MD<br>Time to onset: MD<br>Chemotherapy: MD<br>Radiotherapy: MD	Microorganisms identification technique:<br>Nested PCR n.12<br><br>Target:<br>EBV n.12		and the OPMD control group (prevalence 41.7%)<br><br>Genotype(s) of virus detected: MD	
Gopinath, 2019<br><br>*Crit Rev Oncol Hematol*<br><br>Studies: n.2<br>CCS n.2<br><br>No Meta-analysis<br><br>Low quality<br><br>No Funding	Sample size: n.130 of case/n.125 of the healthy group control<br>Mean age: > 50 y.o.; range MD<br>Gender ratio: MD<br>Country:<br>Taiwan n.127<br>USA n.3<br><br>Risk factors for OSCC: N/D<br>History of OPMD: MD<br>Time to OPMD onset: MD<br>Previous history of malignancies: MD<br>Other comorbidities: MD<br>Other ongoing treatments: MD	Macroscopic features: MD<br>Location: MD<br><br>Staging: MD<br>Grading: MD<br>Microscopic features: MD<br><br>First Diagnosis: MD<br>Time to onset: MD<br>Chemotherapy: MD<br>Radiotherapy: MD	Sample(s): n.130<br><br>Method(s) of sample collection:<br>Saliva test n.130<br><br>Microorganisms identification technique:<br>Incubation in Proteinase K and DNA purification kit n.3<br>QIAamp DNA Blood Kit n.127<br><br>Target:<br>V4-V5 region n.3<br>V4 region n.127	Than the healthy control group<br><br>Bacteroidetes:<br>↑ Bacteroides: > n.50 of OSCC case<br>↑ Porphyromonas<br><br>Proteobacteria:<br>↑ Escherichia: > n.50 of OSCC case<br>Ralstonia<br>Type(s) of Ralstonia species detected :<br>↑ Ralstonia insidiosa<br><br>Actinobacteria:<br>↑ Rothia<br><br>Firmicutes:<br>↑ Bulleidia: > n.50 of OSCC case<br>↑ Gemella<br>↑ Peptostreptococcus<br>↑ Streptococcus<br>↑ Lactobacillus<br>↑ Gemmiger<br>↑ Oscillospira<br>↑ Roseburia	-	-

				Synergistota:<br>↑ Cloacibacillus		
Gupta, 2020<br><br>*Clin Oral Investig*<br><br>Studies: n.1<br>CSS n.1<br><br>No Meta-analysis<br><br>Low quality<br><br>No Funding	Sample size: n.50 of case/n.50 of the healthy control group<br>Mean age: MD<br>Gender ratio: MD<br>Country:<br>India n.50<br><br>Risk factors for OSCC: MD<br>History of OPMD: MD<br>Time to OPMD onset: MD<br>Previous history of malignancies: MD<br>Other comorbidities: MD<br>Other ongoing treatments: MD	Macroscopic features: MD<br>Location: MD<br><br>Staging: MD<br>Grading: MD<br>Microscopic features: MD<br><br>First Diagnosis: MD<br>Time to onset: MD<br>Chemotherapy: MD<br>Radiotherapy: MD	Sample(s): n.50<br><br>Method(s) of sample collection:<br>Saliva test n.50<br><br>Microorganisms identification technique:<br>Culture n.50<br><br>Target: MD	Than the healthy control group (prevalence 8.0%)<br><br>Proteobacteria:<br>Helicobacter<br>Type(s) of Helicobacter species detected:<br>↑ Helicobacter pylori: n.32 (prevalence 64.0%) of OSCC case	-	-
Huybrechts, 2020	Sample size: n.724 of case/n.1188 of the healthy control group	Macroscopic features: MD<br>Location: MD	Sample(s): n.724	↑ Bacteroidetes:<br>↑ Capnocytophaga	-	-

Cancer Epidemiol Biomarkers Prev<br><br>Studies: n.16<br>CS n.2<br>CCS n.14<br><br>No Meta-analysis<br><br>Low quality<br><br>This study was supported by the Intramural Research Program of the National Cancer Institute at the National Institutes of Health and by the Research Foundation-Flanders 12h1519N.	Mean age: MD<br>Gender ratio: MD<br>Country: MD<br><br>Risk factors for OSCC: MD<br>History of OPMD: MD<br>Time to OPMD onset: MD<br>Previous history of malignancies: MD<br>Other comorbidities: MD<br>Other ongoing treatments: MD	Staging: MD<br>Grading: MD<br>Microscopic features: MD<br><br>First Diagnosis: MD<br>Time to onset: MD<br>Chemotherapy: MD<br>Radiotherapy: MD	Method(s) of sample collection:<br>Saliva test n.724<br><br>Microorganisms identification technique: N/D<br><br>Target: MD	↑ Fusobacteria:<br>↑ Fusobacterium<br><br>Actinobacteria:<br>↑ Actinomyces<br><br>↓ Firmicutes:<br>↑ Dialister<br>↑ Peptostreptococcus<br>↑ Parvimonas<br>↑ Streptococcus: NS		

Mallika, 2020<br><br>*Trans Cancer Res*<br><br>Studies: n.11<br>CCS n.11<br><br>No Meta-analysis<br><br>Moderate quality<br><br>No Funding	Sample size: MD<br>Mean age: MD<br>Gender ratio: MD<br>Country: MD<br><br>Risk factors for OSCC: MD<br>History of OPMD: MD<br>Time to OPMD onset: MD<br>Previous history of malignancies: MD<br>Other comorbidities: MD<br>Other ongoing treatments: MD	Macroscopic features: MD<br>Location: MD<br><br>Staging: MD<br>Grading: MD<br>Microscopic features: MD<br><br>First Diagnosis: MD<br>Time to onset: MD<br>Chemotherapy: MD<br>Radiotherapy: MD	Sample(s): MD<br><br>Method(s) of sample collection:<br>Saliva test n.MD<br>Oral swab n.MD<br><br>Microorganisms identification technique:<br>Culture n.MD<br>VIDAS EBV kit n.MD<br>QIAamp Mini Elute Virus Spin kit Digene HPV genotyping RH test n.MD<br>PCR n.MD<br>Spectrophotometer n.MD<br>Gas chromatography n.MD<br>IHC n.MD<br><br>Target: MD	Proteobacteria:<br>Helicobacter<br>Type(s) of Helicobacter species detected:<br>↑ Helicobacter pylori	HPV: MD n./% of OSCC case<br><br>Genotype(s) of HPV detected:<br>↓ HPV-16 n.MD<br>↓ HPV-18: NS n.MD<br><br>EBV: NS<br><br>Than the healthy control group<br>↑ EBV	Than the healthy control group<br>↑ Candida: MD n./% of OSCC case<br><br>Type(s) of fungi species detected: MD
Mauceri, 2022<br><br>*Cancers (Basel)*<br><br>Studies: n.11<br>CCS n.8<br>CS n.2<br>CSCS n.1<br><br>No Meta-analysis<br><br>Critically Low quality<br><br>No Funding	Sample size: n.679 of case/n.480 of the healthy control group/n.153 of the OPMD control group/n.15 of the periodontitis control group<br>Mean age: 56,8 y.o.; range 49,7-63,9 y.o.<br>Gender ratio: 179M/113F/387MD<br>Country:<br>Taiwan n.445<br>China n.97<br>USA n.18<br>Sudan n.59<br>Japan n.60<br><br>Risk factors for OSCC: MD	Macroscopic features: MD<br>Location: MD<br><br>Staging: MD<br>Grading: MD<br>Microscopic features: MD<br><br>First Diagnosis: MD<br>Time to onset: MD<br>Chemotherapy: MD<br>Radiotherapy: MD	Sample(s): n.687<br><br>Method(s) of sample collection:<br>Sputum n.448<br>Oral swab n.156<br>Oral rinse n.18<br>Saliva, subgingival plaque, tumor and healthy surface n.57<br><br>Microorganisms identification technique:<br>QIAamp DNA Blood Mini kit n.347<br>QIAamp MinElute Virus Spin kit n.138	↑ Spirochaetes:<br>↑ Treponema<br><br>↑ Proteobacteria:<br>↑ Campylobacter<br>↓ Lautropia<br>↓ Haemophilus<br>↑ Eikenella<br><br>↑ Bacteroidetes:<br>↑ Alloprevotella<br>↑ Capnocytophaga<br>↑ Prevotella<br><br>Tenericutes:<br>↑ Mycoplasma	-	-

	History of OPMD: MD<br>Time to OPMD onset: MD<br>Previous history of malignancies: MD<br>Other comorbidities: MD<br>Other ongoing treatments: MD		QIAamp Fast DNA Stool Mini kit n.10<br>FastDNA kit n.59<br>Gene Prep Star PI-80X device n.60<br>Modified QIAGEN DNA extraction method n.18<br>E.Z.N.A. soil DNA kit n.47<br><br>Target:<br>Fungal ITS2 region n.59<br>V4 region n.280<br>V3-V4 region n.115<br>V3-V5 region n.138<br>V4-V5 region n.87	↓ Firmicutes: MD n./% of OSCC case<br>↑ Centipeda<br>↑ Selenomonas<br>↑ Dialister<br>↑ Peptostreptococcus<br>↑ Filifactor<br>↑ Peptococcus<br>↑ Catonella<br>↑ Parvimonas<br>↓ Megasphaera<br>↓ Stomatobaculum<br>↓ Granulicatella<br>↓ Veillonella<br>↓ Streptococcus<br>Type(s) of Streptococcus species detected:<br>↓ Streptococcus pneumoniae<br><br>↑ Fusobacteria:<br>↑ Fusobacterium<br>Type(s) of Fusobacterium species detected:<br>F. nucleatum<br><br>↓ Actinobacteria: : MD n./% of OSCC case<br>↓ Scardovia<br>↓ Rothia<br>↓ Actinomyces		
Ramos, 2020<br><br>*Oral Maxillofac Surg*<br><br>Studies: n.4<br>CS n.1<br>CSS n.1<br>CCS n.1<br>MD n.1	Sample size: n.339 of case/n. 214 of the healthy control group/n.124 of the OPMD control group<br>Mean age: MD<br>Gender ratio: N/D<br>Country: MD<br><br>Risk factors for OSCC:<br>Tobacco: n.N/D	Macroscopic features: MD<br>Location: MD<br><br>Staging:<br>Stage I (n.41)<br>Stage II (n.66)<br>Stage IV (n.90)<br>Grading: MD	Sample(s): n.339<br><br>Method(s) of sample collection:<br>Oral rinse n.197<br>Saliva test n.142<br><br>Microorganisms identification technique:<br>MiSeq n.333	Than the healthy control group<br><br>Firmicutes:<br>↑ Streptococcus<br>Type(s) of Streptococcus species detected:<br>↑ S. mitis (in smokers patients)<br>↑ Peptostreptococcus<br>↑ Bacillus	-	-

No Meta-analysis<br><br>Critically Low quality<br><br>This study was supported by the National Council for Scientific and Technological Development (CNPq) (Project:211309/2013-3) and the Foundation for Research Financial Support in the State of Rio de Janeiro (FAPERJ) (Project: E26/1033.001/2012).	Alcohol: n.N/D<br>Betel quid chewing: n.N/D<br>History of OPMD: MD<br>Time to OPMD onset: MD<br>Previous history of malignancies: MD<br>Other comorbidities: MD<br>Other ongoing treatments: MD	Microscopic features: MD<br><br>First Diagnosis: MD<br>Time to onset: MD<br>Chemotherapy: MD<br>Radiotherapy: MD	454/GS Junior n.6<br><br>Target:<br>V4 n.125<br>V3-V4 n.197<br>V3-V5 n.6<br>V4-V5 n.11	↑ Parvimonas<br>↑ Enterococcus<br>↑ Veillonella<br>↑ Stomatobaculum<br>↑ Lactobacillus (abundance with advanced TNM stage)<br><br>Bacteroidetes:<br>↑ Prevotella<br><br>↑ Proteobacteria:<br>↑ Haemophilus<br><br>Actinobacteria:<br>↑ Slackia<br>↑ Rothia<br><br>Tenericutes:<br>↑ Mollicutes<br><br>Spirochaetes:<br>↑ Spirochaetales		
Rapado-González, 2020<br><br>*J Clin Med*<br><br>Studies: n.12<br>CCS n.12<br><br>Meta-analysis<br><br>High quality<br><br>No Funding	Sample size: n.658 of case/n.2210 of the healthy control group<br>Mean age: MD<br>Gender ratio: MD<br>Country:<br>Sweden n.85<br>Iran n.22<br>India n.313<br>USA n.109<br>France n.22<br>Canada n.72<br>Pakistan n.35	Macroscopic features: MD<br>Location: MD<br><br>Staging: MD<br>Grading: MD<br>Microscopic features: MD<br><br>First Diagnosis: MD<br>Time to onset: MD<br>Chemotherapy: MD<br>Radiotherapy: MD	Sample(s): n.658<br><br>Method(s) of sample collection:<br>Oral rinse n.286<br>Saliva test n.372<br><br>Microorganisms identification technique:<br>PCR: n.385<br>qPCR: n.144<br>Nested PCR: n.44<br>Nested PCR and DNA sequencing: n.85	-	↑ HPV: n.271 of OSCC case<br>$p < 0.01$<br><br>Genotype(s) of virus detected:<br>HPV-16: n.57<br>$p < 0.02$<br>HPV-18: n.24<br>NSS<br>N/D HR-HPV: n.164<br>$p < 0.01$<br>N/D LR-HPV: n.8<br>NSS	-

	Risk factors for OSCC: MD<br>History of OPMD: MD<br>Time to OPMD onset: MD<br>Previous history of malignancies: MD<br>Other comorbidities: MD<br>Other ongoing treatments: MD		Target:<br>HPV-6, -10, -11, -13, -26, -31, -32, -33, -34, -35, -39, -40, -42, -44, -45, -51, -52, -53, -54, -56, -58, -59, -61, -62, -66, -67, -68, -69, -70, -71, -72, -73, -76, -81, -82, -83, -84, -89 genome: n.538 of HR-HPV/ n.107 of LR-HPV<br>HPV-16 genome: n.507<br>HPV-18 genome: n.254			
Shaikh, 2015<br><br>*Cancer Epidemiol*<br><br>Studies: n.1<br>N/D n.1<br><br>Meta-analysis<br><br>Low quality<br><br>This study was supported by the Griffith University of Australia.	Sample size: n.34 of case/n.396 of control<br>Mean age: MD<br>Gender ratio: MD<br>Country:<br>India n.34<br><br>Risk factors for OSCC:<br>History of OPMD: MD<br>Time to OPMD onset: MD<br>Previous history of malignancies: MD<br>Other comorbidities: MD<br>Other ongoing treatments: MD	Macroscopic features: MD<br>Location: MD<br><br>Staging: MD<br>Grading: MD<br>Microscopic features: MD<br><br>First Diagnosis: MD<br>Time to onset: MD<br>Chemotherapy: MD<br>Radiotherapy: MD	Sample(s): n.34<br><br>Method(s) of sample collection:<br>Saliva test<br><br>Microorganisms identification technique:<br>PCR n.34<br><br>Target:<br>HPV genome: n.34	-	↑ HPV: n.24 (prevalence 70.60%) of OSCC case<br><br>Genotype(s) of HPV detected:<br>HPV-16: n.MD<br>HPV-18: n.MD	-
Shen, 2023<br><br>*Arch Oral Biol*<br><br>Studies: n.5<br>CCS n.5<br><br>No Meta-analysis	Sample size: n.230 of case/n.219 of the healthy control group/n.205 of the OPMD control group<br>Mean age: N/D<br>Gender ratio: 27M/203MD<br>Country:<br>USA n.18<br>India n.31	Macroscopic features: MD<br>Location: MD<br><br>Staging: MD<br>Grading: MD<br>Microscopic features: MD	Sample(s): n.230<br><br>Method(s) of sample collection:<br>Saliva test n.152<br>Oral rinse n.49<br>Oral swab, plaque swab and saliva test n.29	Than the healthy and the OPMD control group<br><br>↑ Bacteroidetes:<br>↑ Prevotella<br>↑ Alloprevotella<br>↑ Porphyromonas<br>↑ Capnocytophaga	-	Than the healthy and the OPMD control group<br><br>↑ Candida: n.29 of OSCC case

High quality<br><br>This study was supported by the China-Japan Friendship Hospital Research Project Foundation [grant number 2020-1-QN-2].	Taiwan n.N/D<br>China n.29+N/D<br><br>Risk factors for OSCC: MD<br>History of OPMD: MD<br>Time to OPMD onset: MD<br>Previous history of malignancies: MD<br>Other comorbidities: MD<br>Other ongoing treatments: MD	First Diagnosis: MD<br>Time to onset: MD<br>Chemotherapy: MD<br>Radiotherapy: MD	Microorganisms identification technique:<br>Modified QIAGEN DNA n.18<br>Gene Fix Saliva Prep 2 Isolation kit n.31<br>QIAamp DNA Blood mini kit n.124<br>QIAamp DNA Mini kit n.21<br>HiPure tissue and blood DNA kit n.32<br><br>Target:<br>16s rRNA: n.177<br>V4 region n.124<br>V3-V4 region n.103<br>ITS1 n.32	Type(s) of Capnocytophaga species detected:<br>↑ Capnocytophaga sputigena<br><br>Fusobacteria:<br>↑/↓ Fusobacterium<br><br>↑/↓ Firmicutes:<br>↓ Streptococcus<br>↑/↓ Veillonella<br>Catonella<br>Type(s) of Catonella species detected:<br>↑ Catonella morbi<br><br>Proteobacteria:<br>↑ Aggregatibacter<br>↑ Neisseria<br><br><br>Than the OPMD control group<br><br>Bacteroidetes:<br>↓ Prevotella<br>Type(s) of Prevotella species detected:<br>↓ Prevotella oulorum<br>Tannerella<br>Type(s) of Tannerella species detected:<br>↓ Tannerella forsythia<br>Porphyromonas<br>Type(s) of Porphyromonas species detected:<br>↓ Porphyromonas gingivalis<br><br>Firmicutes:<br>↓ Enterococcus<br>↓ Megasphaera<br>Anaeroglobus		↑ Aspergillus: MD n./% of OSCC case<br><br>↑ Acremonium: MD n./% of OSCC case<br><br>↓ Morchella: MD n./% of OSCC case

				Type(s) of Anaeroglobus species detected:<br>↓ Anaeroglobus geminatus<br><br>Proteobacteria:<br>↓ Salmonella<br><br>↓ Saccharibacteria<br><br>Than the healthy control group<br><br>Proteobacteria:<br>↑ Escherichia<br><br>Firmicutes:<br>↑ Gemmiger<br>↑ Oscillospira<br>↑ Roseburia<br>↑ Dialister<br><br>Synergistota:<br>↑ Claocibacillus		
Su Mun, 2021<br><br>*Int J Environ Res Public Health*<br><br>Studies: n.17<br>CCS n.17<br><br>No Meta-analysis<br><br>High quality<br><br>This study was supported by the International	Sample size: n.1089 of case/n.1091 of the healthy control group/n.12 of the control group with benign or malignant thyroid nodules<br>Mean age: MD<br>Gender ratio: MD<br>Country:<br>USA n.356<br>Japan n.72<br>India n.26<br>Taiwan n.460<br>Malaysia n.9<br>China n.80<br>Australia n.63	Macroscopic features: MD<br>Location: MD<br><br>Staging: MD<br>Grading: MD<br>Microscopic features: MD<br>First Diagnosis: MD<br>Time to onset: MD<br>Chemotherapy: MD<br>Radiotherapy: MD	Sample(s): n.1089<br><br>Method(s) of sample collection:<br>Saliva test n.387<br>Oral swab n.139<br>Oral rinse n.536<br>Oral brush n.27<br><br>Microorganisms identification technique:<br>DNA Purification kit n.3<br>DNA easy Blood and Tissue kit n.50	Than the healthy control group<br><br>↑ Firmicutes:<br>↑ Gemella<br>↑/↓ Streptococcus<br>Type(s) of Streptococcus species detected:<br>↑ S. costellatus<br>↑ S. anginosus<br>↑ Peptostreptococcus<br>Type(s) of Peptostreptococcus species detected:<br>↑ Peptostreptococcus anaerobius<br>↑ Gemmiger	-	-

Medical University of Malaysia.	New Zeland n.23<br><br>Risk factors for OSCC: MD<br>History of OPMD: MD<br>Time to OPMD onset: MD<br>Previous history of malignancies: MD<br>Other comorbidities: MD<br>Other ongoing treatments: MD		QIAsymphony virus/Bacteria Midi kit n.121<br>QIAamp DNA Blood Mini kit n.125<br>EURx commercial kit with modifications n.9<br>QIAamp DNA mini kit n.80<br>PowerSoil DNA Isolation kit n.129<br>QIAamp MinElute Virus Spin kit n.138<br>Maxwell 16 LEV Blood DNA kit n.63<br>Phenol-chloroform based DNA extraction n.23<br>QIAamp DNA Microbiome kit n.197<br>Modified QIAGEN DNA extraction n.26<br>Gene Prep Star PI-80X device n.60<br>QIAGEN DNeasy Blood and Tissue kit DNA n.26<br>Purification kit n.27<br>MD n.12<br><br>Target:<br>V4 region n.335<br>V3-V4 region n.461<br>V3-V5 region n.138<br>V4-V5 region n.83<br>V6-V8 region n.63<br>V6-V9 region n.9	↑ Oscillospira<br>↑ Roseburia<br>↑ Granulicatella<br>↑ Dialister<br>↑/↓ Selenomonas<br>Type(s) of Selenomonas species detected:<br>↓ Selenomonas noxia<br>↑/↓ Filifactor<br>Type(s) of Filifactor species detected:<br>↑ Filifactor alocis<br>↑ Peptococcus<br>↑ Catonella<br>↑/↓ Oribacterium<br>↑/↓ Parvimonas<br>Type(s) of Parvimonas species detected:<br>↑/↓ Parvimonas micra<br>↑ Centipeda<br>Ruminococcus<br>Type(s) of Ruminococcus species detected:<br>↓ Ruminococcus gnavus<br>↑/↓ Lactobacillus<br>Type(s) of Lactobacillus species detected:<br>↓ Lactobacillus plantarum<br>↑/↓ Veillonella<br>Type(s) of Veillonella species detected:<br>↑ Veillonella parvula<br>↑/↓ Veillonella dispar<br>↓ Stomatobaculum<br>↓ Megasphaera<br>↓ Dorea<br><br>↑ Bacteroidetes:<br>↑/↓ Bacteroides		

				Type(s) of Bacteroides species detected:<br>↓ Bacteroides ovatus<br>↑/↓ Porphyromonas<br>Type(s) of Porphyromonas species detected:<br>↑ Porphyromonas pasteri<br>↑ Porphyromonas gingivalis<br>↑ Porphyromonas endodontalis<br>↑ Alloprevotella<br>↑/↓ Prevotella<br>Type(s) of Prevotella species detected:<br>↑ Prevotella tannerae<br>↑ Prevotella intermedia<br>↑ Prevotella melaninogenica<br>↑ Prevotella pallens<br>↑ Prevotella copri<br>↑ / ↓ Prevotella nigrescens<br>↑ / ↓ Prevotella nanceiensis<br>↓ Tannerella<br>↑ / ↓ Capnocytophaga<br>Type(s) of Capnoccytophaga species detected:<br>↓ Capnocytophaga ochracea<br>↓ Paludibacter<br>Parabacteroides<br>Type(s) of Parabacteroides species detected:<br>↓ Parabacteroides distasonis<br><br>Proteobacteria:<br>↑ Escherichia<br>↑ Campylobacter<br>Types(s) of Campylobascter species detected:<br>↑ Campylobacter ureolyticus<br>↑ Eikenella<br>↓ Lautropia		

				↓ Actinobacillus<br>↑/↓ Neisseria<br>Type(s) of Neisseria species detected:<br>↓ Neisseria subflava<br>↑/↓ Neisseria bacilliformis<br>↑/↓ Haemophilus<br>Type(s) of Haemophilus species detected:<br>↑ Haemophilus influenzae<br>↑ Haemophilus parainfluenzae<br>↓ Kingella<br>Stenotrophomonas<br>Types(s) of Stenotrophomonas species detected:<br>↓ Stenotrophomonas ruminococcus<br>Aggregatibacter<br>Type(s) of Aggregatibacter species detected:<br>↓ Aggregatibacter segnis<br><br>Synergistota:<br>↑ Cloacibacillus<br><br>↓ Actinobacteria:<br>↑/↓ Rothia<br>Type(s) of Rothia species detected:<br>↓ Rothia mucilaginosa<br>↓ Rothia aeria<br>↓ Rothia dentocariosa<br>↓ Scardovia<br>↑/↓ Actinomyces<br>Type(s) of Actinomyces species detected:<br>↑ Actinomyces odontolyticus<br>↓ Actinomyces oral taxon_170<br><br>↑ Fusobacteria:		

				↑/↓ Fusobacterium<br>Type(s) of Fusobacterium species detected:<br>↑ F. nucleatum<br>↑ F. periodonticum<br>↓ Leptotrichia<br><br>↑ Spirochaetes:<br>↑ Treponema<br><br>Tenericutes:<br>↑ Mycoplasma		
Yu, 2023<br><br>*Heliyon*<br><br>Studies: n.7<br>CCS n.7<br><br>Meta-Analysis<br><br>Moderate quality<br><br>This study was supported by Grant from Qingdao Medical Talents Training Program [VYQ2020Y02].	Sample size: n.413 of case/n.300 of the healthy control group<br>Mean age: 54.5 y.o.; range 32-87 y.o.<br>Gender ratio: MD<br>Country: MD<br><br>Risk factors for OSCC: MD<br>History of OPMD: MD<br>Time to OPMD onset: MD<br>Previous history of malignancies: MD<br>Other comorbidities: MD<br>Other ongoing treatments: MD	Macroscopic features: MD<br>Location: MD<br><br>Staging: MD<br>Grading: MD<br>Microscopic features: MD<br><br>First Diagnosis: MD<br>Time to onset: MD<br>Chemotherapy: MD<br>Radiotherapy: MD	Sample(s): n.413<br><br>Method(s) of sample collection:<br>Saliva test n.188<br>Oral rinse n.225<br><br>Microorganisms identification technique: N/D next-generation sequencing technology n.413<br><br>Target:<br>V4 region n.131<br>V3-V4 region n.215<br>V3-V5 region n.10<br>V4-V5 region n.47<br>V6-V8 region n.10	Than the healthy control group<br><br>↑ Fusobacteria:<br>↑ Fusobacterium<br>p=0.000<br><br>↑/↓ Proteobacteria:<br>↑/↓ Haemophilus<br>p= 0.000<br>↑ Escherichia<br>↓ Neisseria<br><br>↑/↓ Actinobacteria:<br>↓ Rothia<br><br>↑/↓ Firmicutes:<br>↑/↓ Streptococcus<br>p= 0.032<br>↑/↓ Veillonella<br>↑ Lactobacillus<br>↑ Blautia<br>↑ Clostridium<br>↑ Dorea<br>↑ Faecalibacterium<br>↑ Megamonas<br>↑ Phascolarctobacterium	–	–

				↑/↓ Bacteroidetes: 50.0% of OSCC case<br>↑ Prevotella: NSS<br>↑ Alloprevotella<br>↑ Alistipes<br>↑ Bacteroides		

Abbrevations: number, "n."; years old, "y.o."; percentage, "%"; greater than, ">"; less than, "<"; missing data, "MD"; not defined, "N/D"; not statistically significant, "NSS"; not significant, "NS"; p-value, "p"; male, "M"; female, "F"; case-control study, "CCS"; cohort study, "CS"; cross-sectional study, "CSS"; cross-sectional cohort study, "CSCS"; Oral Squamous Cell Carcinoma, "OSCC"; Oral Potentially Malignant Disorder, "OPMD"; United States of America, "USA"; polymerase chain reaction, "PCR"; real time polymerase chain reaction, "RT-PCR"; quantitative polymerase chain reaction, "qPCR"; immunohistochemistry, "IHC"; Ribosomal Ribonucleic Acid, "rRNA"; RiboNucleic Acid, "RNA"; DeoxyriboNucleic Acid, "DNA"; subspecies, "ssp"; Fusobacterium, "F."; Campylobacter, "C."; Streptococcus, "S."; Human Papilloma Virus, "HPV"; Low Risk Human Papilloma Virus, "LR-HPV"; High Risk Human Papilloma Virus, "HR-HPV"; Epstein Barr Virus, "EBV"; increased, "↑"; decreased, "↓"; not investigated, "−".
