# Supplementary material for: Oral Bacteria, Virus and Fungi in Saliva and Tissue Samples from Adult Subjects with Oral Squamous Cell Carcinoma: An Umbrella Review
Source: Cancers (Basel). 2023 Nov 22;15(23):5540. doi: 10.3390/cancers15235540 (PMC10705713; doi:10.3390/cancers15235540)
Supplement: Supplementary file 1 [file cancers-15-05540-s001.zip › Supplementary file S3.pdf]

Supplementary Table n.S3 – Quality assessment of included studies

Table S3. Quality assignment of included studies according to AMSTAR-2. First author, year, reference, sixteen AMSTAR-2 items to be evaluated, quality of the study.

Studies	Item 1	Item 2	Item 3	Item 4	Item 5	Item 6	Item 7	Item 8	Item 9	Item 10	Item 11	Item 12	Item 13	Item 14	Item 15	Item 16	Quality
Ayuningtyas, 2022	Y	N	Y	Y	Y	N	N	PY	N	N	-	-	N	N	-	Y	Critically Low
Bronzato, 2020	Y	Y	Y	PY	Y	Y	Y	PY	PY	N	Y	N	Y	Y	Y	Y	Moderate
Chaitanya, 2016	Y	N	Y	PY	Y	Y	N	N	N	N	Y	N	N	N	N	Y	Critically Low
Christianto, 2022	Y	Y	Y	PY	N	N	N	PY	PY	N	Y	N	N	N	N	Y	Critically Low
de Carvalho Melo, 2021	Y	Y	Y	PY	Y	Y	Y	PY	PY	N	-	-	N	Y	-	Y	Low
de Lima, 2014	Y	N	Y	PY	N	N	N	PY	N	N	-	-	N	N	-	N	Critically Low
de Lima, 2019	Y	N	N	PY	Y	N	Y	Y	N	N	Y	Y	N	Y	N	N	Critically Low
Gopinath, 2019	N	Y	N	Y	Y	Y	Y	Y	PY	N	-	-	N	Y	-	Y	Low
Guo, 2018	Y	N	N	Y	Y	N	Y	N	N	N	Y	Y	N	Y	N	Y	Critically Low
Gupta, 2020	Y	Y	Y	Y	Y	Y	N	Y	Y	N	-	-	N	Y	-	Y	Low
Haghshenas, 2022	N	Y	Y	Y	Y	N	N	PY	Y	N	N	Y	Y	Y	Y	Y	Critically Low
Hobbs, 2006	N	N	Y	PY	Y	N	PY	PY	N	N	Y	N	N	N	N	Y	Critically Low
Huybrechts, 2020	Y	Y	Y	N	Y	Y	Y	N	Y	N	-	-	Y	Y	-	Y	Low
Kreimer, 2005	Y	PY	Y	PY	N	Y	N	Y	N	N	Y	N	N	Y	N	N	Critically Low

Mallika, 2020	Y	Y	Y	Y	N	N	Y	Y	Y	N	-	-	Y	N	-	Y	Moderate
Mauceri, 2022	Y	PY	Y	Y	N	Y	Y	N	Y	N	Y	N	N	Y	N	Y	Critically Low
Miller, 2001	Y	N	N	Y	N	N	N	N	N	N	Y	N	N	Y	N	N	Critically Low
Muthusamy, 2023	N	PY	Y	PY	N	N	PY	N	N	N	Y	N	N	Y	N	Y	Critically Low
Nandi, 2021	Y	PY	Y	N	N	N	N	PY	N	N	-	-	N	Y	-	Y	Critically Low
Rahman, 2023	Y	Y	Y	PY	N	Y	Y	Y	N	N	Y	N	N	Y	N	Y	Critically Low
Ramos, 2020	Y	Y	Y	Y	N	N	N	Y	N	N	-	-	N	N	-	Y	Critically Low
Rapado-González, 2020	Y	Y	Y	Y	Y	Y	Y	Y	Y	N	Y	Y	Y	Y	Y	Y	High
Shaikh, 2015	Y	Y	Y	PY	N	Y	N	PY	Y	N	Y	N	Y	Y	N	Y	Low
She, 2017	Y	Y	Y	PY	Y	Y	Y	PY	Y	N	Y	N	Y	Y	Y	Y	Moderate
Shen, 2023	Y	Y	Y	Y	Y	Y	Y	Y	PY	N	-	-	Y	Y	-	Y	High
Sivakumar, 2020	Y	Y	Y	Y	Y	N	N	Y	Y	N	Y	Y	Y	Y	N	Y	Low
Su Mun, 2021	Y	Y	Y	Y	Y	Y	Y	Y	Y	N	-	-	Y	Y	-	Y	High
Syrjänen, 2011	Y	N	Y	Y	N	Y	N	Y	N	N	Y	N	N	Y	N	Y	Critically Low
Termine, 2008	PY	N	N	Y	Y	Y	N	N	N	N	Y	N	N	Y	N	N	Critically Low
Yang, 2019	Y	Y	Y	Y	N	Y	Y	Y	Y	N	Y	Y	Y	Y	Y	Y	Moderate
Yu, 2023	Y	Y	Y	PY	N	Y	Y	PY	Y	N	Y	N	Y	Y	Y	Y	Moderate

Zhu, 2012	Y	N	Y	Y	N	Y	Y	PY	N	N	Y	N	N	Y	N	Y	Critically Low

Abbreviations: Yes, "Y"; No, "N"; Partial Yes, "PY"; Not valuable, "-"; underlined items are those considered critical by AMSTAR-2.
